# Electrochemical Impedance Spectroscopy of (Photo)electrochemical Systems: A Practical Guide

**DOI:** 10.1021/acsmeasuresciau.6c00109

**Published:** 2026-08-07

**Authors:** Thomas Moehl, Erin Service, Wooseok Yang, S. David Tilley

**Affiliations:** † Department of Chemistry, 27217 University of Zurich, Winterthurerstrasse 190, Zurich 8057, Switzerland; ‡ School of Chemical Engineering, Sungkyunkwan University (SKKU), Suwon 16419, Republic of Korea; § SKKU Institute of Energy Science and Technology (SIEST), Sungkyunkwan University, Suwon 16419, Republic of Korea

**Keywords:** electrochemical impedance spectroscopy, photoelectrochemical water splitting, hydraulic analogy of electricity, metal−electrolyte interface, p−n junction, semiconductor–electrolyte interface, (photo)cathode, impedance fitting, equivalent circuit, Mott–Schottky analysis

## Abstract

Electrochemical impedance spectroscopy (EIS) is a powerful technique for investigating (photo)­electrochemical systems. Although several comprehensive introductions to EIS are available, the selection of experimental parameters and the subsequent data processing, fitting, and interpretation remain challenging, particularly for newcomers. Here, we address these challenges by providing a practical, beginner-oriented introduction to EIS that emphasizes both conceptual understanding and in-depth analysis. We discuss measurements under direct current and alternating current conditions, the use of equivalent circuits (ECs), and best practices for acquiring reliable and physically meaningful EIS data. To develop understanding in a modular and systematic way, we start from two well-defined benchmark systems—a metal–electrolyte interface and a silicon p–n junction diode—and then extend the analysis to a buried-junction photoelectrochemical device. This stepwise approach enables the construction and justification of a realistic EC model for the complete photoelectrochemical system and demonstrates how robust parameters can be extracted from EIS measurements.

## Graphical abstract

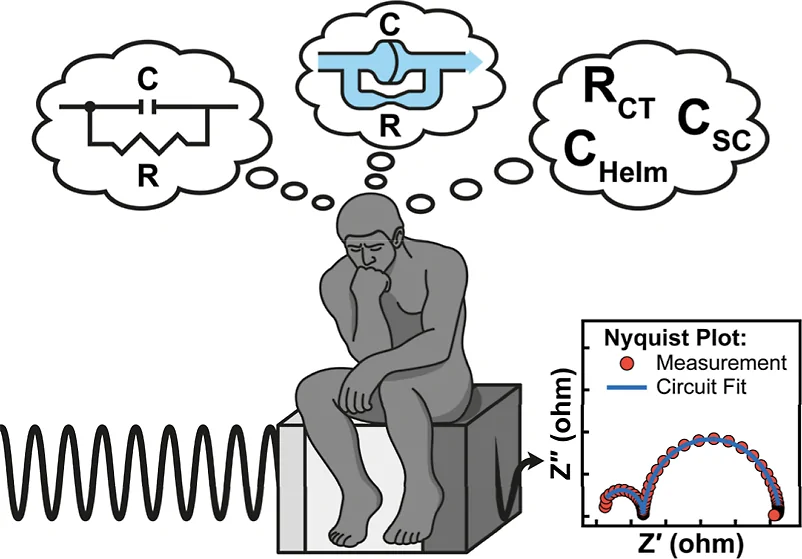

## Introduction

Electrochemical impedance spectroscopy (EIS) is a powerful and versatile analytical technique widely used in various fields such as catalysis,^1^ fuel cells,^2^ sensors,^3^ photovoltaics (PV),^4,5^ photoelectrochemical (PEC) water splitting^6–9^ and batteries.^10–12^ EIS excels in extracting the behavior of individual components within multicomponent systems and offering insights into their potential-dependent behavior and/or corrosion properties. It is a nondestructive analytical technique essential for studying material properties as well as electrical and electrochemical interfaces.

Despite its advantages, EIS can often appear mysterious and complex. While direct current (DC) measurements are generally more intuitive, transitioning from DC to alternating current (AC) can present significant challenges. Incorrect experimental design, improper execution, and false fitting and misinterpretation of data are common pitfalls for those unfamiliar with EIS. Although comprehensive introductions to EIS exist,^13–18^ we observe persistent difficulties among students in our laboratory when performing and interpreting EIS measurements, especially for complex systems such as multijunction PEC electrodes.

This work aims to clarify common misunderstandings and misinterpretations, though it does not claim to be comprehensive. It should help researchers to begin EIS measurements confidently, to extract meaningful information, and to encourage refined interpretations of existing equivalent circuit (EC) models and the development of completely new EC models. The manuscript provides a bridge between electrochemical systems and semiconductor devices, such as those found in PEC electrodes. We discuss the methodology of EIS, the proper execution of measurements, the fitting of the data to an EC, and the implications of the observed impedance. Using a hydraulic analogy, we illustrate resistive and capacitive elements in both DC and AC measurements. Two model systems—a metal–electrolyte interface and a p–n junction solar cell—are then treated in detail. The insights gained are finally transferred to the analysis of a buried-junction p-Si/TiO_2_/Pt PEC photocathode.^19^ We believe that the approach presented will help beginners in EIS to begin the investigation of new (photo)­electrochemical systems and also establish and verify their ECs.

To further support reproducibility and good practice, the first section of the Supporting Information provides an EIS analysis scheme and a practical checklist for reliable EIS measurements. Subsequent sections in the Supporting Information offer practical tips on the systems investigated and their fitting procedures as well as a glossary of common EIS terms.

## Conceptual Foundations and EIS Analysis

### Visualizing Voltage, Current, and Impedance through a Hydraulic Analogy

Impedance, in its simplest definition, is the resistance and phase-shifted reactance of a system to an AC perturbation. To build an intuitive understanding of impedance, we begin with a conceptual hydraulic analogy for DC and AC conditions. Figures 1 and 2 illustrate the distinct properties of DC and AC currents. Under steady-state DC conditions, time-dependent responses are not accessible. In contrast, AC measurements inherently involve time-dependent (or phase-shifted) responses of the system to an external periodic electrical stimulus.

**Figure 1:**
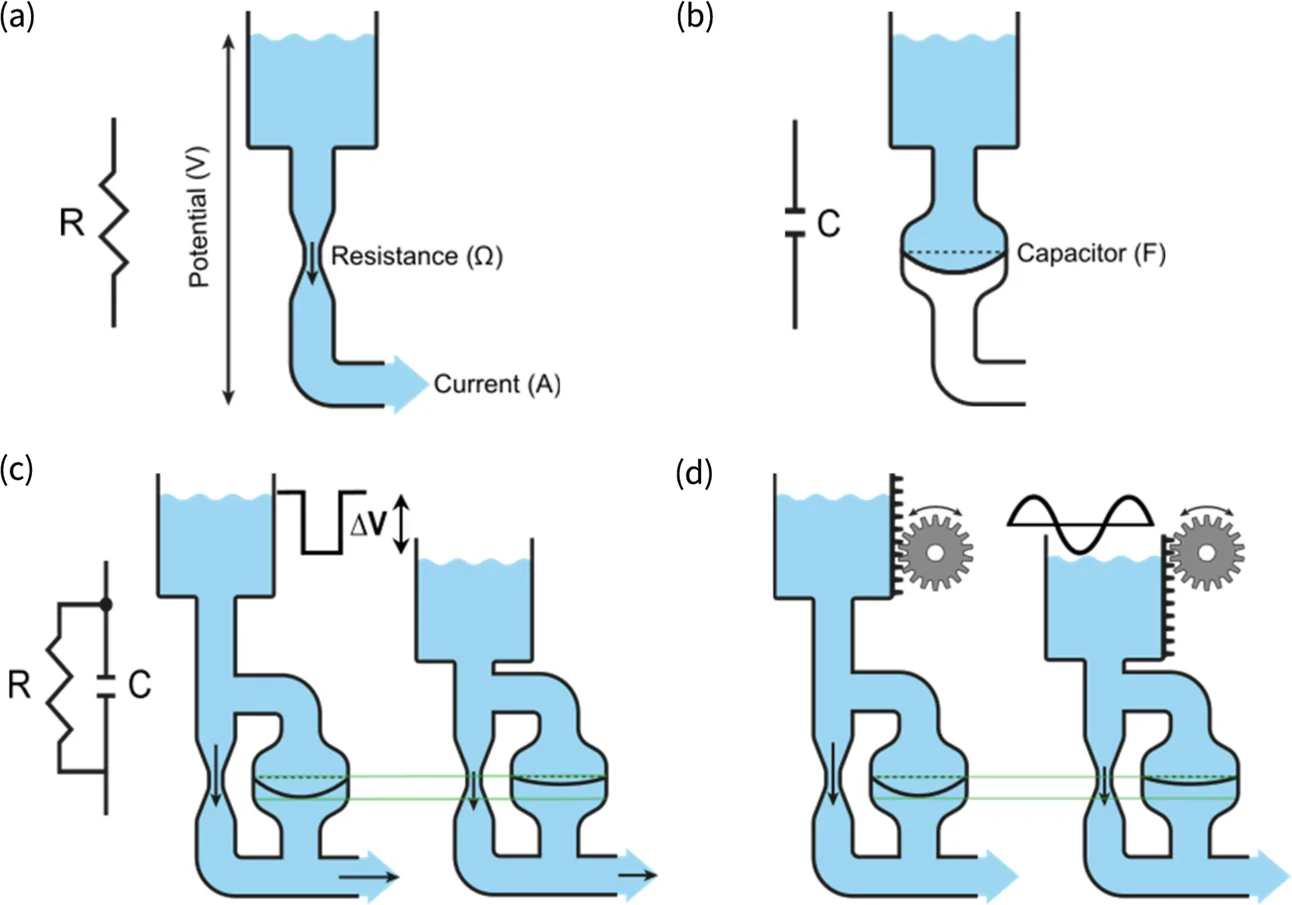
(a) Water flowing downstream through a resistance, where the potential energy mimics the applied voltage and the water flow represents the electrical current. (b) Flexible membrane is stretched by water’s potential energy and stores energy, equivalent to a capacitor storing charge. (c) Parallel connection of a resistor and a membrane undergoing a potential step. (d) Similar arrangement to (c), but now the pressure (voltage) change is a smooth, periodic oscillation controlled by the gear wheel; different oscillation frequencies can be achieved by changing the gear speed.

**Figure 2:**
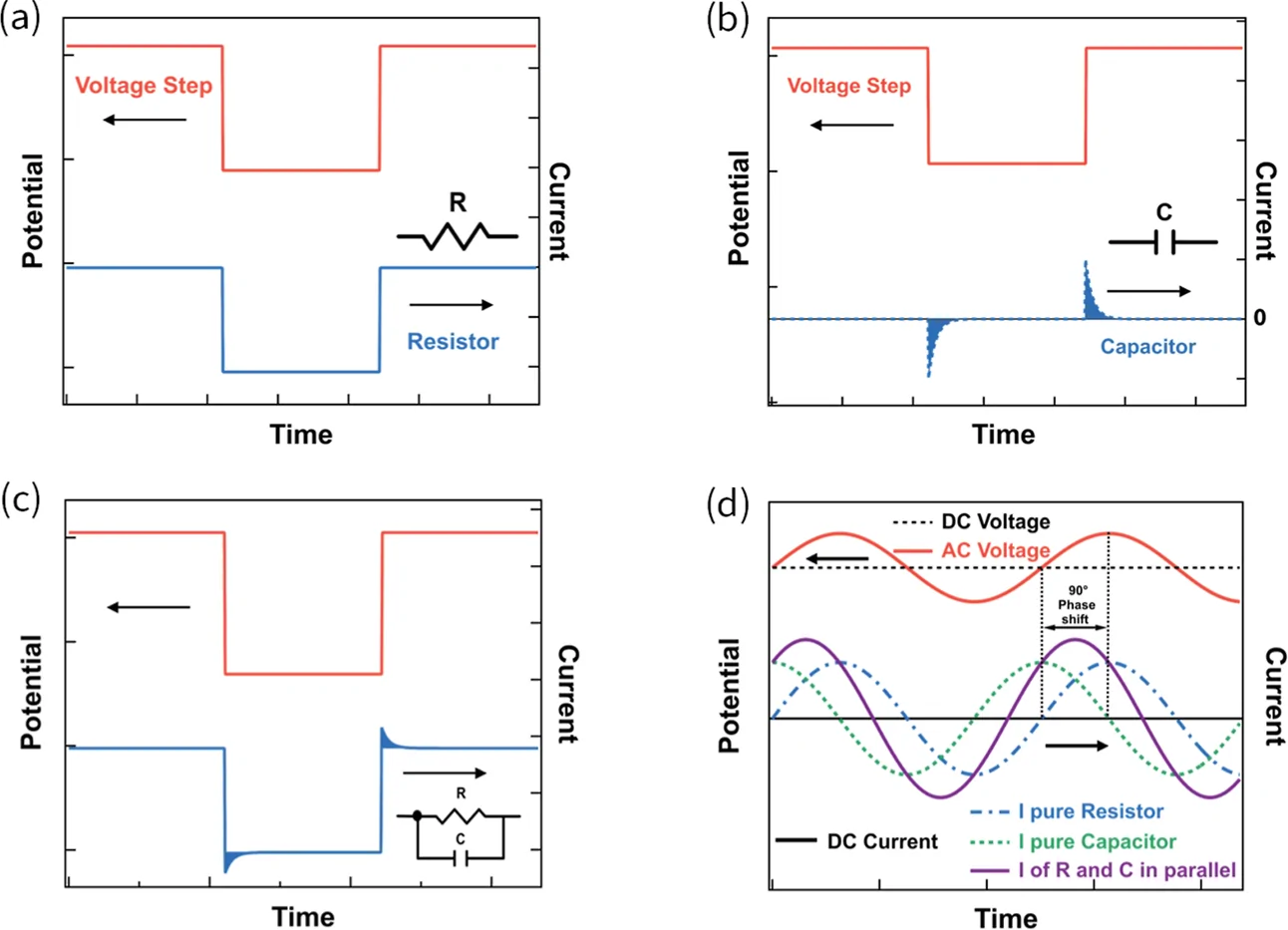
(a) Current–voltage characteristics of a resistor versus time. (b) Current–voltage characteristics of a capacitor versus time. (c) Current–voltage characteristics of a resistor and a capacitor in parallel ((RC)_p_) versus time. (d) Current–voltage characteristics of a resistor (blue), a capacitor (green), and their parallel combination (RC)_p_ (violet) against a periodic potential perturbation at a single frequency.

Consider a large water tank connected to a downstream tube, through which water flows. The water molecules can be interpreted as point charges, and the potential energy of water at a given height can be interpreted as voltage, where the water pressure is driven by gravity acting on individual water molecules that causes water to flow downhill (Figure 1a). Similarly, in an electric circuit, voltage represents the potential energy per unit charge, driving electric current through an electrical circuit. The potential energy of water in the tank (voltage) provides the driving force for water flow (current).

As water flows downhill with constant pressure, this represents unidirectional or “DC” current flow, which depends on the water tank’s height (voltage). Increasing the height of the tank leads to a higher water flow, just as increasing the voltage in an electrical system leads to a higher current. If the tube narrows, limiting the water flow, this narrow section is analogous to a resistor in an electric circuit, restricting the amount of water (current) passing through the tube (Figure 1a).

To mimic a capacitor in this hydraulic analogy, the tube running downstream contains an impermeable membrane (Figure 1b). As a result, water cannot flow freely through the tube. Although the net water flow is zero, the membrane is stretched by the applied water pressure and generates a counterpressure. The small amount of water stored in the stretched membrane is analogous to the charge stored in a capacitor.

For a simple resistive tube, as shown in Figure 1a, a voltage step function (i.e., an instantaneous change in the height of the water basin) leads to a corresponding change in current, as illustrated in Figure 2a. In contrast, when an impermeable membrane is present (Figure 1b), a potential step causes transient water flow associated with the storage and release of water (or charge). These transient currents are characteristic of capacitive behavior and are shown in Figure 2b.

We can now combine resistive and capacitive elements in direct analogy with an equivalent electrical circuit. The series configuration where a resistor is placed in the same tube either upstream or downstream of the membrane (Figure S1 in Supporting Information Note 1) will not be discussed in detail here. The parallel arrangement of the two elements, denoted as (RC)_p_, is illustrated in Figure 1c. In this configuration, water flows through the resistive tube as before, while a parallel flow path includes an impermeable membrane. Within this parallel channel, the membrane stretches under pressure, generating counterpressure and storing energy. Again, this capacitive element cannot be directly observed under steady-state downstream flow conditions (DC).

To fully describe and understand the system, information about both the resistive and the capacitive components is required. Time-dependent (AC) measurements provide access to the capacitive behavior of the system. The importance of the capacitance elements in electrical and electrochemical systems is further elucidated in the following sections.

Let us now consider a perturbation experiment that reveals the properties of the (RC)_p_ system depicted in Figure 1c,d. When the height of the water tank is changed stepwise (without altering the tube properties or introducing turbulence or other flow-related artifacts), the DC current through the resistive pathway responds instantaneously (Figures 1c and 2c). In addition, the charge moving into or out of the capacitor gives rise to transient currents following the potential step. These transient, time-dependent measurements provide direct insights into the stored charge within the system. Thus, AC and other time-dependent measurements are essential for uncovering information about system components that remain hidden under steady-state conditions[Fn fn1].

Establishing a clear and unambiguous analogy between AC electrical measurements and water flow is not trivial, particularly when illustrating a periodic voltage perturbation. Nevertheless, consider the following thought experiment: imagine a gear wheel attached to the water tank that smoothly raises and lowers it in a periodic manner, again, without altering the tube properties or introducing turbulence or other flow-related artifacts (Figures 1d and 2d). The periodic motion of the gear wheel modulates the water pressure, causing the resulting flow (current) to increase and decrease periodically (see also Figure S2). This modulation occurs around an underlying DC voltage and current and thus mimics AC conditions, analogous to those employed in EIS. In an EIS measurement, the current therefore oscillates periodically around a constant value determined by the DC steady-state current at the midpoint of the applied perturbation amplitudes (Figure 2d).

A simple resistor subjected to an AC potential perturbation produces a DC response with no phase shift relative to the applied potential (Figure 2d, blue dotted line), as it can respond instantaneously to changes in potential. In contrast, a system consisting solely of a capacitor exhibits a transient or phase-shifted current response of 90° to a varying potential. Under these conditions, only the storage and release of charge (a transient current) are determined (Figure 2d, green dotted line), though no faradaic DC current flow from the capacitor can be observed. The magnitude of the resulting current depends on the stretchability of the membrane material or, equivalently, on the capacitance of the capacitor. In a parallel combination of a resistor and a capacitor, these two current contributions—the instantaneous resistive response and the phase-shifted capacitive response—superimpose to yield the total measured current at a given frequency (Figure 2d, violet).

The vertical movement of the water tank at a certain frequency causes the membrane to stretch periodically. However, the membrane’s deformation, governed by its intrinsic mechanical properties, may occur on a different time scale than the imposed change of water pressure/potential. In the case of a high-frequency height or potential perturbation, the membrane may respond too slowly, resulting in little or no stretching and therefore only in a small or zero capacitive contribution. Only at lower frequencies does the membrane begin to store and release energy (charge), and at sufficiently low frequencies, it can become fully stretched (charged) and fully relaxed (discharged) in one period of potential swing. This behavior explains why individual capacitive elements can be resolved in systems containing multiple capacitances, provided that these elements have sufficiently different relaxation time constants (or membrane stiffness). This effect is called frequency-dependent relaxation or frequency dispersion. It also highlights the necessity of probing a wide range of frequencies to detect different capacitive elements within a system (along with their associated resistive elements). In EIS, the frequency of the voltage perturbation is therefore varied over several orders of magnitude. Electrochemical reactions, such as faradaic charge transfer at the electrode–electrolyte interface, are typically observed in the millihertz to hertz range. Responses associated with semiconductor properties, such as space–charge capacitance, can often be detected in the kilohertz to megahertz range, though in PEC devices these features may appear at considerably lower frequencies due to the additional interfacial processes involved.

A concise mathematical formalism describing the AC voltage perturbation and the corresponding Euler’s formula is presented in Supplementary Note 2. We also recommend that the readers familiarize themselves with Kirchhoff’s laws and the common ECs (Supplementary Notes 3 and 4) as well as the two most common plots to present EIS data, the Nyquist plot and the Bode plot (see Supplementary Note 5). General tips on how to perform a reliable EIS measurement can be found in Information Box 1 and further hints for the fitting and analysis of the EIS data in Information Box 2 and Supplementary Note 6. Finally, to account for roughness or local chemical variations on the electrode surface, the ideal (parallel-plate) capacitor in an EC is often replaced by a constant phase element (CPE). This modification offers in most cases a more realistic representation of the investigated system and typically results in improved fitting of the EIS data. Afterward, the CPE values must be converted into effective capacitance values. Several conversion formulas exist for this purpose, differing in their underlying physical assumptions and in the information required for their application (see Supplementary Note 7 for further details on the CPE and its conversion into capacitance). A good overview on the different conversion possibilities is given by Hirschorn et al.^22^ Throughout the manuscript and the presented data, only the Brug formula has been used for the conversion of a CPE to a capacitance. Using the Brug formula is justified, especially for the Helmholtz and the space–charge capacitance.^23^

The choice between the different ECs (see Supplementary Note 4) for the fitting procedure is not arbitrary but reflects the physical nature of the processes inside a device under operation. In all ECs, *R*_series_ appears at the outermost position, as it represents ohmic losses from cables, contacts, and the electrolyte solution that are common to all current pathways. In the Randles circuit, a single charge-transfer process at the metal–electrolyte interface is described by one (RC)_p_ element with an optional Warburg element to account for diffusion limitations. The parallel placement of an R and C implies that a junction simultaneously stores charge and allows current to flow just like a charge-transfer resistance and a Helmholtz capacitance at a metal–electrolyte solution interface. When two distinct processes are present and operate independently, the Voigt configuration is appropriate, as both (RC)_p_ elements are electrically decoupled. In this case, their resistances can be directly read from the *x*-axis of the Nyquist plot. On the other hand, when the two processes are instead coupled, implying that one process modulates the charging of the other, as occurs when charge transfer is coupled to a charged interfacial or adsorption layer, the Maxwell circuit applies. In this latter case, the resistance values must be extracted through fitting rather than read directly from the plot. Finally, the nested (Matryoshka) circuit describes a situation in which a single charge-transfer process is associated with two distinct capacitances in sequence, commonly encountered when transfer occurs through surface or defect states in photoelectrochemical systems.

To study the EIS of a buried-junction PEC photocathode, we will first examine the EIS of a metal–electrolyte solution interface, followed by a single p–n junction solar cell. This approach represents a dissection of a photoelectrode into its main components: the electrochemical interface for the hydrogen evolution reaction and the p–n junction for photon absorption and charge generation. In a final step, we will show how these two systems can be combined to characterize and understand PEC photoelectrodes.

### Metal–Electrolyte Solution Interface

To mimic the catalytic interface of a PEC photocathode using platinum as a hydrogen evolution catalyst on its surface, we investigated a polycrystalline Pt cathode in 1 M HClO_4_ as a first example of a system.

For the metal–electrolyte solution interface, we used HClO_4_, although 1 M H_2_SO_4_ is commonly used in PEC systems. Perchlorate ions are generally only weakly adsorbing on platinum surfaces, yielding a more pronounced separation of the time constants in the measured EIS data. Within the conditions studied here, sulfate and perchlorate differ only marginally: the same Pt electrode in 1 M H_2_SO_4_ produced spectra of the same form as in HClO_4_, differing only in how clearly the time constants could be resolved. Nevertheless, one should always be mindful of the electrolyte ions present in solution as they can sometimes profoundly influence the reaction kinetics, double-layer structure, and even the reaction pathway through competitive adsorption on active sites. These effects can manifest directly in the extracted EIS parameters and, in extreme cases, can render a more complex equivalent-circuit model necessary.^24,25^

A schematic representation of the metal–electrolyte interface overlaid with an electrochemical energy scale is shown in Figure 3a. The electrochemical behavior of this interface is governed by two principal elements: the charge-transfer resistance (*R*_CT_), associated with faradaic charge transfer, and the Helmholtz capacitance (*C*_Helm_), which describes nonfaradaic charge accumulation at the interface. The Helmholtz capacitance arises from the Coulombic attraction of oppositely charged electrolyte ions toward the charged electrode surface. To a first approximation, *C*_Helm_ is typically treated as a constant, with reported values in the range of 10–50 μF cm^–2^.^26–28^ Acting in parallel with the charge-transfer resistance, the Helmholtz capacitance is analogous to a membrane placed alongside a narrowed resistive tube in the hydraulic analogy (see EC in Figure 1c and inset in Figure 3b).

**Figure 3:**
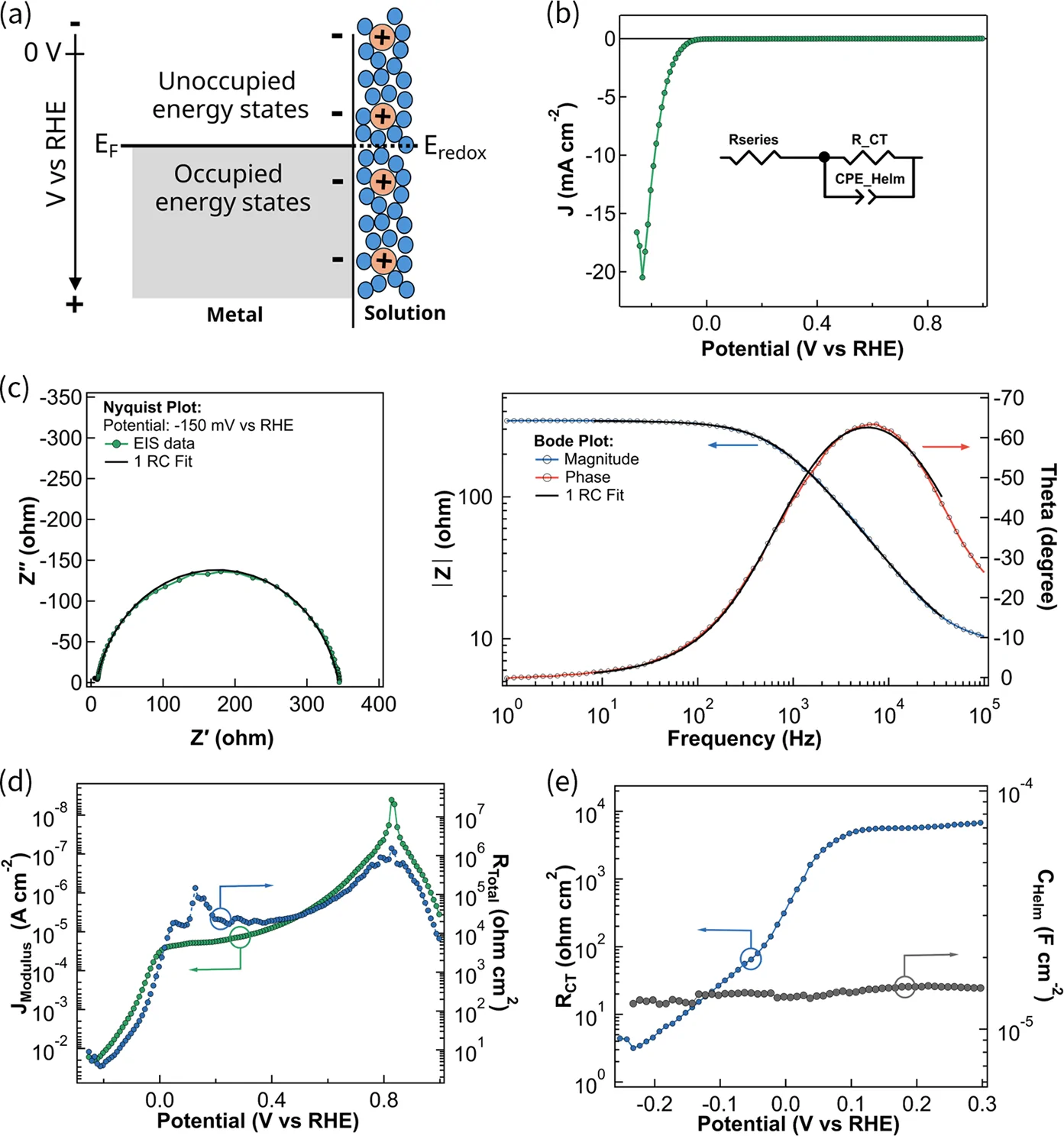
(a) Schematic of a metal–electrolyte solution interface overlaid with an electrochemical energy scale. (b) DC current–voltage characteristics of a polycrystalline Pt electrode in 1 M HClO_4_ during an EIS measurement (extracted from the lowest frequency of each single EIS spectrum); inset shows the EC used for the hydrogen evolution region. (c) Nyquist and Bode plots at −150 mV vs RHE (electrode area: 0.0314 cm^2^). Note that Z″ in the Nyquist plot is displayed in the positive quadrant, although it corresponds to increasingly negative values. (d) Logarithm of the DC current from (b) together with the total resistance determined for this *J*–*V* curve by differentiating voltage with respect to current (see main text). (e) *R*_CT_ and *C*_Helm_ obtained from the EIS fitting.

As a first step following the EIS measurement, the DC current is extracted from the lowest frequency applied during the experiment (Figure 3b). Figure 3c shows the corresponding Nyquist and Bode plots recorded under pronounced hydrogen evolution conditions at −150 mV vs RHE. The spectra exhibit a single time constant, represented by one semicircle in the Nyquist plot. This simple EC is valid only within the hydrogen evolution regime of a Pt electrode. In other potential regions—such as the Pt oxidation regime or the underpotential hydrogen deposition region—the impedance spectra display more than one time constant. Consequently, more complex ECs must be employed to accurately describe the system over the whole potential range (Supplementary Note 8).

Figure 3d shows the absolute current density on a logarithmic scale as a function of applied potential. The plotted differential resistance d*V*/d*J*, obtained by differentiating the DC *J*–*V* curve, represents the total system resistance, *R*_Total_, encompassing all resistive contributions—series resistance, charge-transfer resistance, and any other resistive elements. This value should therefore equal the sum of all resistances extracted from the EIS fitting. Equivalently, integrating the sum of all EIS-derived resistances over the current density should reproduce the DC–potential curve measured directly. Agreement between these two independently obtained quantities serves as a quality check, confirming that the EIS EC model accounts for all resistive contributions present in the system.

Current density and resistance are often displayed on logarithmic scales because these quantities can span several orders of magnitude. The logarithmic representation, therefore, enables clearer visualization and a more meaningful comparison across the full range of values.

Selected fitting results are presented in Figure 3e, showing only the extracted values of *R*_CT_ and *C*_Helm_. A more detailed discussion can be found in Supplementary Note 9, while the Nyquist and Bode plots of the measured data at five different potentials are shown in Supplementary Note 10. The extracted *R*_CT_ has the same magnitude as the total resistance of the system in Figure 3d and a similar voltage dependence. It reflects the role of R_CT_ as the dominant resistive contribution to the overall resistance of the system (the series resistance is only about 7 ohms over the whole bias potential range measured). The determined Helmholtz capacitance lies within the expected range of several tens of μF cm^–2^, with only minor variation over the presented potential range.

The most dramatic changes of the system can be observed in the hydrogen evolution regime at potentials lower than 0 V vs RHE. The current first increases exponentially, while the resistance shows the inverse behavior, decreasing exponentially. Finally, at increasingly negative potentials, the current drops due to vigorous bubble formation. The bubble formation starts to decrease the active surface area of the electrode while also disrupting the diffusion layer inside the electrolyte solution in the vicinity of the electrode. Under these conditions, the mass transport has to occur through an intermittent electrolyte contact leading to effective diffusion limitation of H^+^ in the vicinity of the electrode (represented by a Warburg diffusion element in the EC) and measurable concentration–polarization effects.^29,30^

Overall, this analysis demonstrates that EIS provides direct access to both faradaic and nonfaradaic processes at the metal–electrolyte interface and enables their clear separation under hydrogen evolution conditions. The extracted resistive and capacitive parameters are physically meaningful and in good agreement with the expected values for polycrystalline Pt. These results establish a reliable reference for interpreting more complex electrochemical and photoelectrochemical interfaces discussed in the following sections.

### p–n Junction Diode

Let us now consider a solid-state photovoltaic device (e.g., a silicon solar cell) that absorbs light and converts it into electricity. First, we relate a diode under DC conditions to a one-way water valve (Figure 4). A one-way valve allows water to flow only in one direction when the water pressure is applied in a certain direction. At zero water pressure, analogous to 0 V, the valve remains closed (Figure 4a; also represented by the origin in Figure 4e). When applying pressure (potential energy), water flows freely, much like electric current that flows through a forward-biased diode (Figure 4b,c). As the connecting tube narrows exponentially near the ball valve, the water flow does not switch abruptly from zero to maximum but rather increases exponentially with a linear increase in pressure until the system reaches its maximum flow capacity (Figure 4c). Conversely, when pressure is applied in the reverse direction, the valve closes and blocks the flow (Figure 4d; reverse region in Figure 4e).

**Figure 4:**
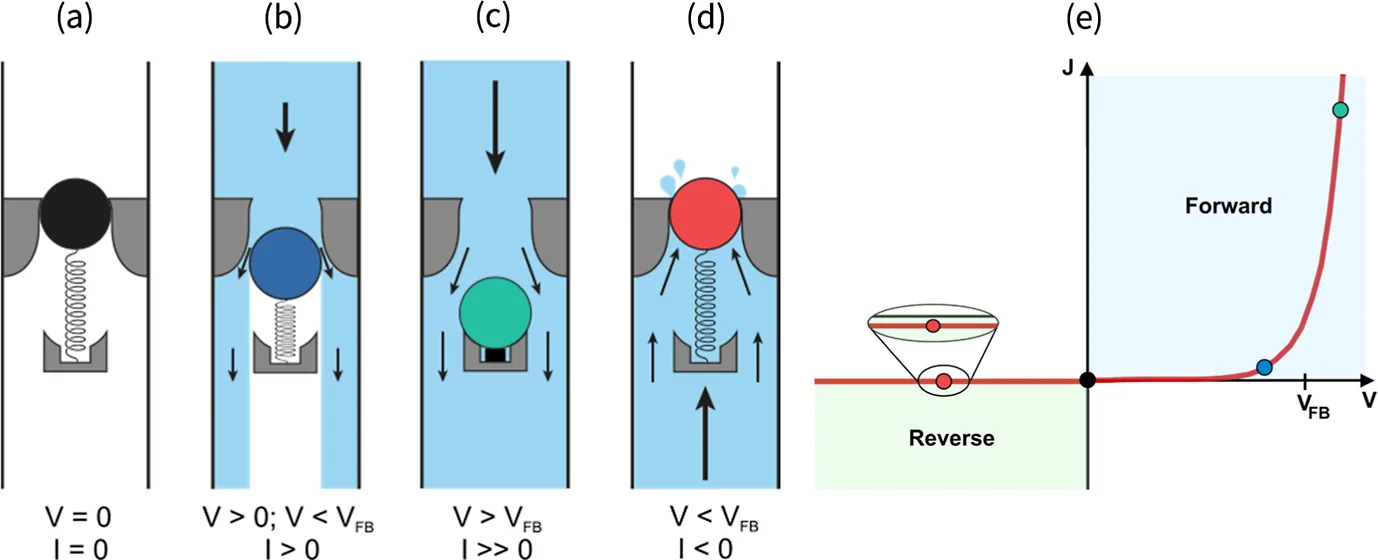
(a) Diode, a one-way valve, at 0 V. (b) Diode under low forward bias. (c) Diode under strong forward bias (maximum current flow). (d) Small leakage current under reverse bias. (e) Diode *J*–*V* curve under forward (anodic) bias and reverse (cathodic) bias.

To mimic this behavior electronically, semiconductor diodes—particularly p–n junctions—are commonly employed. A diode can switch between blocking and conducting states for charge carriers depending on the applied potential, reflecting the forward- and reverse-pressure regimes described in the water-valve analogy. Figure 5a illustrates the junction under depletion conditions (charge blocking), while Supplementary Note 11 also presents the junction in accumulation (charge conducting).

**Figure 5:**
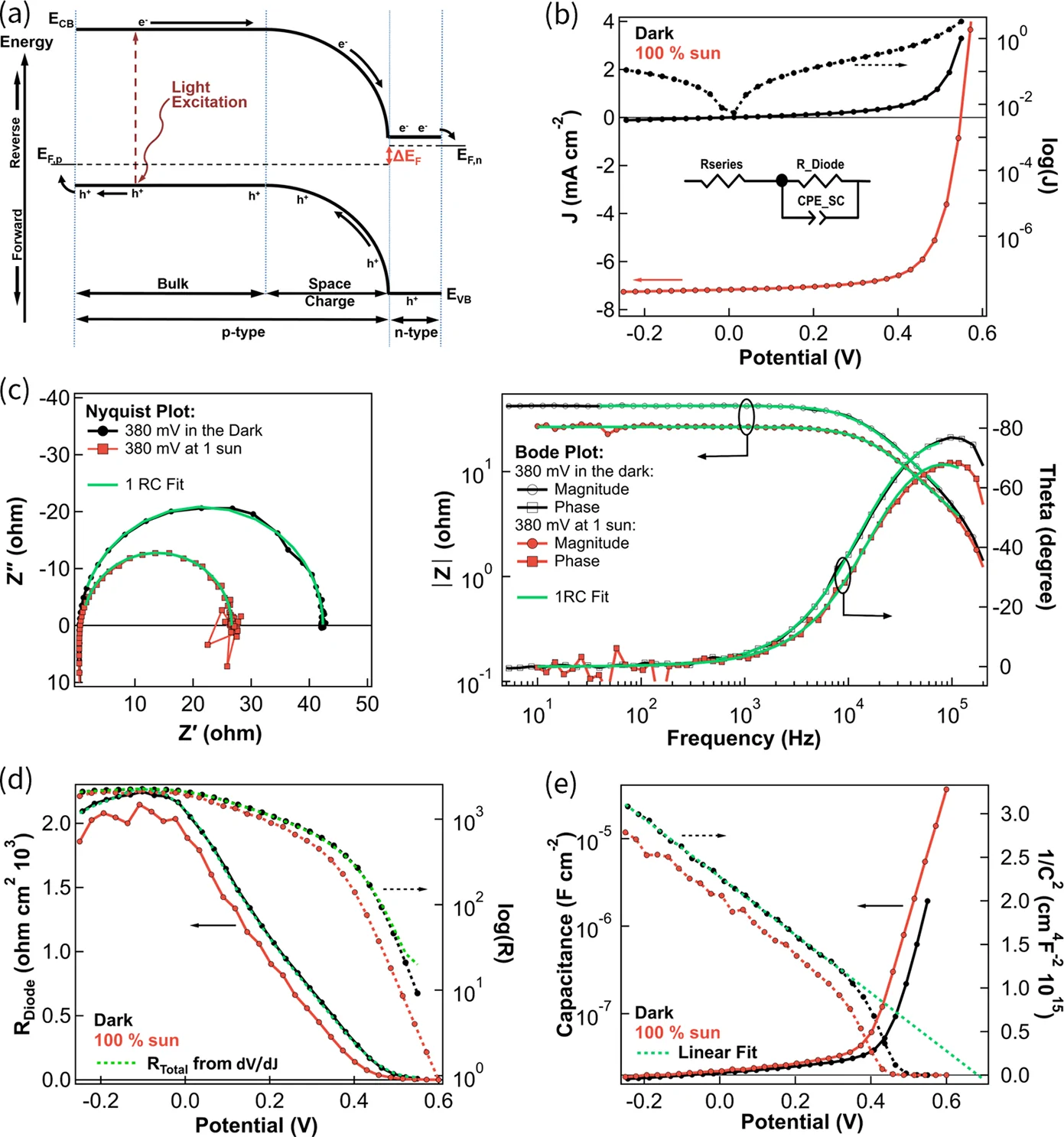
(a) p–n^+^ junction in depletion under illumination. The quasi-Fermi level splitting under illumination, Δ*E*_F_ = *E*_F,n_–*E*_F,p_, corresponds to the photovoltage through Δ*E*_F_ = *q*·*V*. (b) Current–voltage characteristics of a silicon solar cell in the dark and under 1 sun illumination intensity using white-light LED. Inset shows the EC used to fit the EIS data. (c) Nyquist and Bode plots at 380 mV in the dark and under 1 sun white-light LED illumination. Note that Z″ in the Nyquist plots is displayed in the positive quadrant, although it corresponds to increasingly negative values. (d) *R*_Diode_ plotted in linear and in log scale. (e) *C*_SC_ and Mott–Schottky plot (1/(*C*_SC_)^2^).

A diode consists of p-type (hole-conducting) and n-type (electron-conducting) regions. In a p–n^+^ diode (as presented in Figures 5 and S9), the heavily doped n^+^ region has a high density of free carriers (in this case electrons). When the two regions contact, the Fermi levels in each of the materials (representing the potential energy of the electrons) will align through the exchange of charge. The equilibration at the contact of the two differently doped regions induces a band bending in the lower-doped p-type semiconductor and the formation of a space–charge layer and its associated capacitance (*C*_SC_). Scanning the potential into reverse bias conditions, the p–n junction inhibits electrical current, while band bending increases. In contrast, when scanning the potential into forward bias, the junction becomes increasingly transmissive for current as the band bending at the p–n junction reduces, thereby reducing the barrier for charge transfer (Supplementary Note 11).

When scanning the potential far enough into forward bias, a condition where there is no band bending can be achieved, the so-called flat band potential (*E*_FB_). The flat band condition corresponds to the maximum theoretical photovoltage (*V*_OC_) the p–n junction can provide. In this potential region, the dark current is already substantial.

The total current density of a photodiode can be expressed with the diode equation^31^

1 $$J_{total}=J_{diode}-J_{photo}=J_{0}\left(e^{qV/nkT}-1\right)-J_{photo}$$

with *J*_total_ as total current density, *J*_photo_ as light-generated current density, *J*_diode_ as diode current density, *J*_0_ as reverse/dark saturation current density, and *q*, *V*, *n*, *k*, and *T* as the elementary charge, the applied voltage, the diode ideality factor, the Boltzmann constant, and the absolute temperature, respectively. The diode equation is presented in standard formulation (in contrast to solar cell literature in which the signs of the different currents are inversed).

Examining eq 1 qualitatively, one can deduce several key features. The photocurrent is a fixed value over the whole potential range, depending only on the light intensity. The dark current of the diode has (in the photovoltaic mode under forward bias) a unidirectional flow of current that varies exponentially with the applied potential. It represents a counter-current to the light-generated photocurrent, reducing its value. Under illumination at *V*_OC_, the diode current is equal to the light-generated photocurrent, and all the photogenerated charge carriers are compensated by the counter-current from the diode’s dark current (i.e., the net current is zero).

In its simplest form, the elements observed in the impedance measurement of a solar cell comprise a single parallel RC element.^4,32^ It represents the space–charge capacitance and the diode resistance. The resistance (*R*_diode_) we determine by this model represents solely the DC diode current (*J*_diode_), even under illumination conditions, since the photocurrent is constant to a first approximation (for a more detailed discussion, see Supplementary Notes 12 and 13). To complete the EC model, a series resistance accounting for cables and contact resistances is implemented (Figure 5b inset). This model already captures the fundamental behavior of the silicon p–n junction in response to voltage and light. Nevertheless, there exists a large number of publications with more advanced p–n junction models and refined interpretation of the measured results. General literature on EIS of silicon solar cells can be found from von Hauff^13^ and Mora-Seró et al.,^4^ while more advanced models like the two-diode model of a p–n junction can be found from, e.g., Yadav et al. and others to name only a few.^33–36^ We note that the analysis of EIS data from p–n junction silicon solar cells can be relatively straightforward in contrast to organic solar cells,^37,38^ perovskite solar cells,^39–41^ or dye-sensitized solar cells.^42^

Although the resulting EC resembles that used for a metal–electrolyte interface, the underlying (photo)­physical processes and their interpretation differ fundamentally. In solar cells, minimizing dark currents is crucial, and therefore, a high diode resistance is desired. In contrast, for metal–electrolyte interfaces, low resistances are typically sought to achieve high catalytic activity for the target electrochemical reaction.

As with the previous systems, EIS data processing begins by extracting the DC current from the lowest frequency measured during the impedance experiment (Figure 5b). In the dark, the current is cathodic at negative applied voltages and changes sign at a positive bias, reflecting the transition from reverse to forward diode operations. Representative Nyquist and Bode plots recorded both in the dark and under 1 sun illumination at 380 mV are shown in Figure 5c (further EIS data are presented in Supporting Information Note 14).

In a subsequent step, the voltage–current relationship is used to derive the total resistance of the system (Figure 5d). Applying the developed EC model to the EIS data enables extraction of the diode resistance (*R*_Diode_) and the space–charge capacitance of the monocrystalline p–n^+^ junction silicon solar cell (Figure 5d,e). The extracted diode resistance shows an exponential decrease when entering the forward bias regime (potential applied >0 V). It closely matches the total resistance obtained from the derivative of the voltage–current relationship, thereby confirming the validity of the fitting procedure (Figure 5d).

In contrast, the space–charge capacitance increases as the system transitions from the reverse to forward bias, reflecting the narrowing of the space–charge region due to reduced band bending at the p–n junction interface (Figure 5e). *C*_SC_ increases continuously (while still under depletion conditions), and the magnitude typically lies in the nF range, making it more than 2 orders of magnitude smaller than the Helmholtz capacitance. This difference in magnitude can also be used to differentiate between *C*_SC_ and *C*_Helm_ in more complex systems where both capacitances can be detected.

To extract further information about the junction and the semiconductor properties, the space–charge capacitance in the depletion regime is analyzed using a Mott–Schottky (MS) formula (see eq 2), as shown in Figure 5e (see Supplementary Note 15 for details on Mott–Schottky analysis).

2 $$\frac{1}{C_{SC}^{2}}=\frac{-2}{{A^{2}N}_{A}\varepsilon \varepsilon_{0}q}\left(V-V_{FB}+\frac{kT}{q}\right)$$

where ε is the dielectric constant of the electrode, ε_0_ is the vacuum permittivity, *q* is the charge of the electron, *N*_A_ is the acceptor density, *A* is the (surface) area, *V* is the applied potential, *V*_FB_ is the flat band potential, *k* is the Boltzmann’s constant, and *T* is the temperature. Here, the area is included explicitly in the MS formula, following the usual convention.

By this analysis, both the flat band potential (or built-in voltage) and the doping density of the semiconductor can be determined, as well as the doping type. These parameters represent key characteristics of the light-absorbing material and, in the present case, yield a built-in potential of approximately 700 mV for the p–n junction and a p-type doping density of 3.9 × 10^15^ cm^–3^.[Fn fn2]

### Buried-Junction PEC Photocathode

A buried junction PEC photocathode combines the two device concepts discussed above: a solid-state light absorber and a catalytic electrochemical interface.

Figure 6a presents the schematics of the PEC system for which a new EC should be applied, a buried junction p-silicon absorber with an n-type TiO_2_ heterocontact, and Pt as the hydrogen evolution catalyst. The system is depicted under depletion conditions. Schematics corresponding to other potential regimes are provided in Supporting Information Note 16.

**Figure 6:**
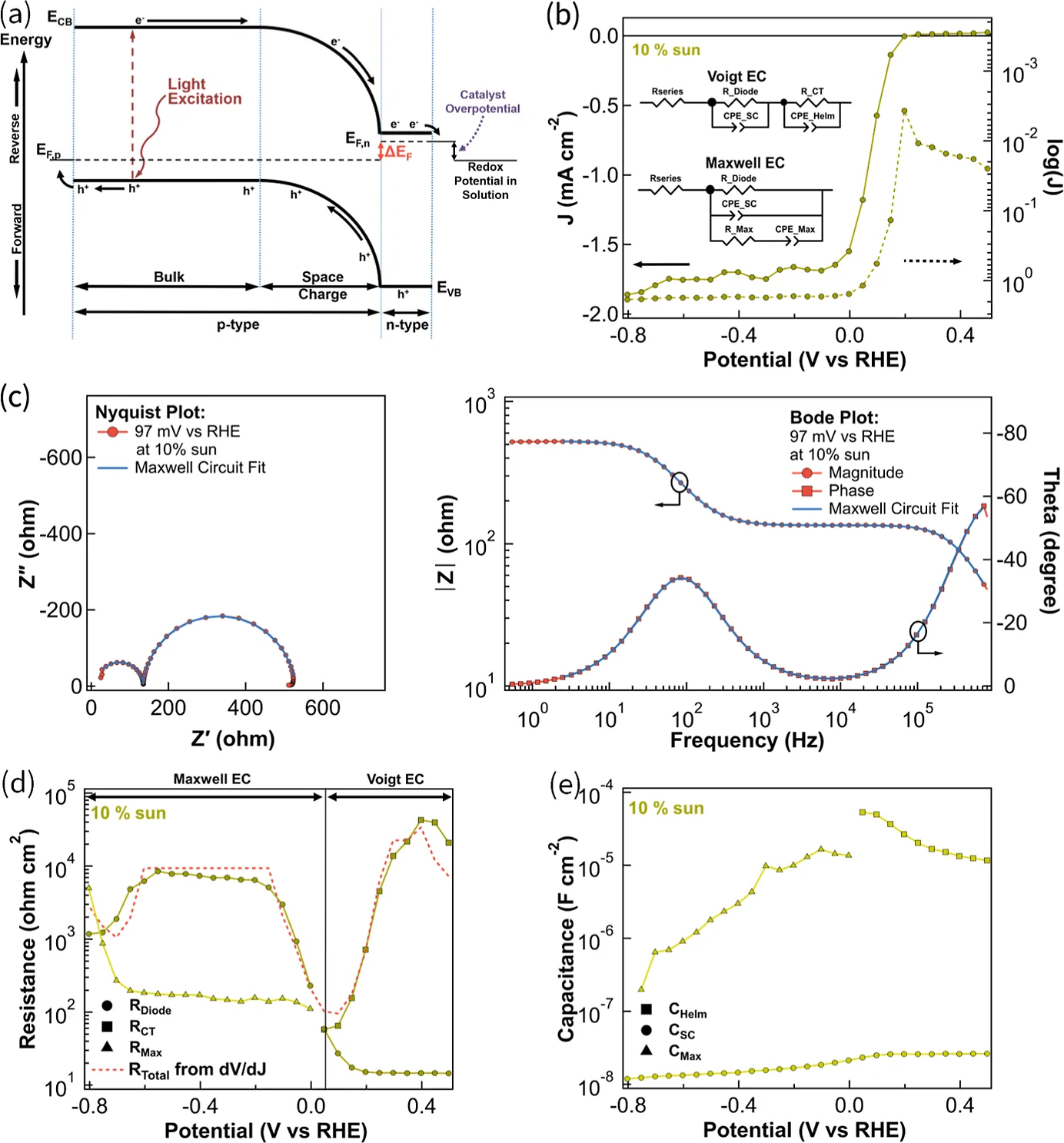
(a) Schematic of a buried-junction PEC device under illumination. (b) DC current extracted from the lowest EIS frequency (0.2 Hz) under 10% sun intensity using LED. Inset shows the Voigt EC (before photocurrent onset) and the Maxwell EC (after photocurrent onset). (c) Nyquist and Bode plots at 97 mV vs RHE under 10% sun white-light LED illumination (see Supplementary Note 17). Note that Z″ in the Nyquist plots is displayed in the positive quadrant, although it corresponds to increasingly negative values. (d) Resistances with an indication of which EC has been used in a certain potential region and (e) capacitances from the EIS fitting under 10% sun white-light LED illumination.

This device was measured under white-light LED illumination at 10% sun equivalent intensity to minimize gas bubble formation, which can otherwise interfere with EIS measurements (see also Information Box 1). The DC current at 10% light intensity was extracted from the EIS data and is plotted on both linear and logarithmic scales in Figure 6b. Over the entire investigated potential range, the Nyquist and Bode plots exhibit two semicircles, indicating the presence of two distinct time constants (Figure 6c and Supporting Information Note 17). A similar behavior with two time constants was also observed at different illumination intensities (Supporting Information Note 18).

Multiple semicircles under depletion and photocurrent-generation conditions have likewise been observed with other heterojunction PECs.^9,44^ These observations raise the question of which photophysical and electrochemical processes are captured in the present system and which equivalent-circuit model most appropriately describes the essential features governing the behavior of this PEC photocathode. Importantly, the assignment of the underlying (photo)­physical and electrochemical processes—such as recombination, charge transfer, ion migration, intercalation, or corrosion—must be performed individually for each device type and cannot be transferred indiscriminately between different electronic or (photo)­electrochemical systems. Similar silicon-based PEC systems have already been studied by EIS, though with much thinner or crystalline TiO_2_,^45–47^ to detect only the space–charge capacitance (with no complete EC model provided),^45^ or the EC model used was much more complex than the elements visible in our EIS data.^48^ Therefore, we aimed to provide a complete EC model for the different potential regions of these PEC photocathodes.^19^ The following discussion demonstrates how an interested researcher can systematically approach the EIS analysis of such a complex and initially unknown system.^19^

The DC photocurrent shown in Figure 6b resembles the *J*–*V* characteristic of a solar cell but exhibits notable differences in the potential region above the open-circuit voltage (>0.15 V vs RHE). In this regime, only a small anodic current is observed, in contrast to the rapid exponential increase typically seen in solar cells under forward-bias conditions. This behavior can be attributed, first, to the blocking nature of the n-type TiO_2_ contact. Owing to its deep-lying valence band, TiO_2_ prevents holes from the silicon valence band from reaching the electrolyte interface. Second, the oxidation reaction is further limited by the scarcity of energetically suitable hole acceptors in the electrolyte solution (i.e., molecular hydrogen), which suppresses anodic charge transfer.

When the appropriate EC is not known a priori, the insights gained from the metal–electrolyte interface and the solar-cell device can be used as guiding references to interpret the EIS response of the PEC electrode. By combining these reference frameworks with targeted additional experiments, the underlying processes can be systematically identified, and an appropriate EC model can be developed.

### Potential Region before the Photocurrent Onset

Before the photocurrent onset, *R*_CT_ from the Pt catalyst must be observable together with the Helmholtz capacitance (which should be in the range of tens of μF/cm^2^) in the low-frequency range of the EIS data. *R*_CT_ is expected to decrease exponentially near the photocurrent onset, as illustrated in Figure 6d. In the hydrogen evolution region, where charge transfer becomes extremely fast, this element may no longer be observable.

A second element can be assigned to either the diode (solid-state) element, the adsorption of hydrogen on Pt, or the intercalation of protons in the TiO_2_ layer. The associated capacitance of this element is more than 100 times smaller than that of the pure Pt electrode (Figure 6e) or atomic layer deposition (ALD) TiO_2_ in contact with the electrolyte.^9,49^ It is, however, in the same range as the space–charge capacitance of the investigated p-type silicon in our buried-heterojunction photocathode (see Supporting Information Notes 18 and 19).^19^ By the capacitance magnitude, its potential dependence, and comparison to reference systems, we exclude hydrogen adsorption and proton intercalation as dominant contributions.

On the basis of these observations, the EC for this potential region can be described as a Voigt-type model comprising two parallel RC elements (see Figure 6b): one representing the diode (*R*_Diode_ and *C*_SC_) and one for the charge transfer into the electrolyte solution (*R*_CT_ and *C*_Helm_).

The placement of circuit elements reflects the physical architecture of the device. The two RC elements are arranged in a Voigt configuration because the buried p–n junction and the semiconductor–electrolyte interface are physically distinct and electrically independent before the photocurrent onset. Each is governed by its own time constant—consistent with the two resolvable semicircles observed in the Nyquist plot. *R*_diode_ and *C*_SC_ capture the solid-state photovoltage-generating process, while *R*_CT_ and *C*_Helm_ describe charge transfer at the Pt–electrolyte interface.

### Potential Region Coinciding with Hydrogen Evolution

After the photocurrent onset, the diode resistance is expected to rise sharply, analogous to the behavior observed in the solar-cell device discussed above. Once the steady-state photocurrent is reached, this resistance remains essentially constant, as confirmed in Figure 6d. On the other hand, *R*_CT_ is drastically reduced by the applied/generated (photo)­potential and the catalyst used, which ultimately leads to the vanishing of the RC element representing the charge transfer into the electrolyte solution through the Helmholtz capacitance.

*C*_SC_ slowly decreases as the potential scans toward reverse bias (for the p-type silicon). The intrinsic space–charge properties of silicon should remain unchanged, regardless of whether it is used in a PEC device or a solid-state solar cell. If an MS plot with the expected slope (based on the semiconductor doping) can be generated from one of the measured capacitances, it can be confidently assigned to *C*_SC_ (see Figure 6e and Supporting Information Note 20).

Using different overlayers such as SiO_2_ or crystalline TiO_2_ yields similar *J*–*V* behavior compared to amorphous ALD TiO_2_, but only one RC element is observed in the Nyquist plots in the steady-state current condition. The diode resistance extracted from these devices can be compared to the *R*_Diode_ value determined using Maxwell and Voigt circuits for the amorphous TiO_2_ device. Only the *R*_Diode_ value obtained from the Maxwell circuit shows consistent agreement.^19^

Further evidence for the correct assignment of *R*_Diode_ comes from the reverse-bias region of the *J*–*V* curve (Figure 6b). From −0.6 V vs RHE onward, the total current increases, while *J*_photo_ remains unchanged—meaning the additional current originates solely from the diode current (*J*_diode_). This behavior, known as a photoshunt, becomes particularly pronounced at higher light intensities (Figure S17a) and is further reflected in the apparent change of the diode resistance in this potential region (Figures 6d and S17d). The effect originates from an increase in diode current under illumination that is independent of the photogenerated current (see Supplementary Notes 11, 12, and 13 for a detailed explanation of shunt currents).

At this point, we have a clear indication of which EC is appropriate for the PEC photocathode under study. Before the photocurrent onset, the Voigt circuit is appropriate: the low-frequency RC element represents charge transfer to the electrolyte solution, characterized by *R*_CT_ and *C*_Helm_, while the high-frequency element corresponds to the diode, characterized by *R*_Diode_ and *C*_SC_. When entering the potential region of hydrogen evolution, the Maxwell circuit is used, implying that the EC used has to be changed during the fitting procedure. Again, the diode part consisting of the diode resistance and the space–charge capacitance is detected paired with an element describing the charging at the p-silicon/amorphous ALD TiO_2_ interface. In the Maxwell circuit configuration, unlike the Voigt circuit used before the photocurrent onset, the two RC elements are no longer electrically independent but coupled—one element modulates the charging behavior of the other. This coupling reflects the intimate interfacial coupling between the silicon absorber and the TiO_2_ overlayer that becomes dominant in this potential region. As a consequence, the resistance values cannot be read directly from the semicircle diameters in the Nyquist plot but must be extracted through fitting. The electronic coupling of these two elements is most likely related to the known defect band in ALD amorphous TiO_2_ since proton intercalation into the TiO_2_ could be excluded.^19^

## Conclusion

In this work, we have approached the EIS analysis of a buried-junction multilayer photocathode by combining the behavior of a light-absorbing semiconductor diode with the electrochemical charge transfer occurring at a metal–electrolyte interface. Beyond explaining the distinct device types and interfaces and their signatures in EIS measurements, we have provided detailed guidance on how such measurements are performed, as well as how the resulting spectra can be fitted and analyzed. It is important to emphasize that the observations and conclusions presented here may not be directly applicable to all multilayer photocathodes. Nevertheless, clear similarities are evident in the EIS response among the absorber systems studied in our laboratory, including Si, Cu_2_O, and Sb_2_Se_3_.^9,44^

While this work does not cover all aspects of EIS and ultimately presents a simplified view of both a complex measurement technique and an energy-generating photoelectrochemical system, we believe that the approach outlined here will help beginners gain confidence in performing EIS measurements. It provides a practical framework for the initial fitting and analysis of such photoelectrochemical devices, demonstrating that even complex multilayer systems become tractable when approached through the stepwise combination of well-understood reference systems.

## Experimental Information

Electrochemical measurements of a Pt electrode: Measurements were performed with a 2 mm diameter multicrystalline Pt electrode from CHInstruments. The electrolyte was 1 M HClO_4_. The potentiostat was a SP-300 from Biologic. A Pt wire served as a counter-electrode. The reference electrode was an Ag/AgCl with a potential of 197 mV vs NHE. No specific measures were taken to exclude oxygen. EIS measurements were performed with 70 frequencies in logarithmic scale from 1 MHz down to 1 Hz with a perturbation voltage of *V*_rms_ = 7 mV. Bias potential steps were 10 mV from 0.8 V to −0.4 V vs Ag/AgCl with an equilibrium time of 20 s at each bias potential.

Measurements of the p–n junction solar cell: The p–n junction solar cell measured was a simple 9 cm^2^ monocrystalline silicon solar cell, as used for DIY projects. It was purchased from Conrad Electronics (TRU COMPONENTS MF-6609291 Solarmodul). The EIS measurements were performed with a Biologic SP-300 potentiostat. The stabilization time was 5 s. The bias potential steps were 28 mV between −0.3 and 0.55 V. Seventy frequencies were applied in logarithmic scale from 1 MHz to 0.2 Hz. The perturbation voltage was *V*_rms_ = 0.7 mV. Devices were illuminated using a white LED Lumiled Luxeon Rebel (SP-12-W5, cool white).

Fabrication and measurements of the PEC photocathode: p-doped ⟨100⟩ Si wafers with a thickness of 525 ± 25 μm and resistivity of 1–30 Ω cm were purchased from Si-Mat Silicon Materials. To clean the wafers, they were sonicated for 10 min each in acetone, water with deconex 11 UNIVERSAL, water, and isopropyl alcohol and dried in an N_2_ stream. The cleaned Si wafers were then further cleaned at 80 °C for 10 min in a 1/1/5 H_2_O_2_ (30%, Supelco Analytical): NH_4_OH (25%, Honeywell): water solution, followed by 10 min in a 1/1/5 H_2_O_2_ (30%, Supelco Analytical): HCl (37%, Merck): water solution. Then, the native oxide was etched through submersion in a 2% HF solution for 30 s followed by rinsing with water. For devices with a SiO_*x*_ protection layer, this final etching step was not performed.

A PICOSUN R-200 ALD system was used to deposit 100 nm of a-TiO_2_ on the Si substrates using water and tetrakis­(dimethylamino)­titanium (TDMAT) as the O and Ti precursors, respectively. The chamber inside the ALD was held at 120 °C during the deposition. A 1.6 s pulse of TDMAT and 7.0 s N_2_ purge were followed by a 0.1 s water pulse, followed by a 6.0 s N_2_ purge to deposit a single layer of a-TiO_2_. These steps were repeated until the desired thickness was achieved (1860 cycles). The thickness of the a-TiO_2_ layer was determined by using a J.A. Woollam Co. alpha-SE ellipsometer. For devices with a *c*-TiO_2_ protection layer, the samples were subsequently heated to 500 °C for 1 h on a hot plate in air.

The back contact for the device was made with GaIn eutectic (Ga 75.5%/In 24.5%, Merck). The backside of Si was scratched with a diamond pen, shortly cleaned with N_2_ flow, and then the eutectic was applied. Ag paste (Loctite EDAG 1415M) was then used to fix a piece of Cu foil to the back, which functioned as the current collector. The active area of the device was defined with epoxy (Loctite EA9466 and EA9461).

The Pt catalyst was deposited using a pulsed photoelectrodeposition method with 10 mM H_2_PtCl_6_ (ACS reagent, ≥37.50% Pt basis, Merck) in 2 M HCl. Under 1 sun illumination, the voltage was stepped between an open-circuit potential (for 5 s) and a negative bias (for 0.25 s) that reduced Pt onto the surface of the device. For devices with a-TiO_2_ and *c*-TiO_2_ protection layers, the bias voltage was −0.15 V vs Ag/AgCl. For devices with the SiO_*x*_ protection layer, the bias voltage was −0.55 V vs Ag/AgCl. The voltage steps were repeated until 10 mC cm^–2^ was passed. To finally activate the catalyst, cyclic voltammetry in 1 M H_2_SO_4_ under 0.5 sun was performed (25–50 cycles) to condition the catalyst and improve the fill factor.

For all PEC experiments, 1 M H_2_SO_4_ was used as the electrolyte solution. The counter-electrode was isolated from the device using a Nafion membrane to prevent oxygen entering the solution in which hydrogen evolution was performed. H_2_ was generated by a second potentiostat to maintain a fixed redox potential in the electrolyte solution. The constant current set for hydrogen generation was −0.2 mA throughout the measurements.

All photoelectrochemical tests were performed with a Biologic SP-300 potentiostat. Devices were illuminated using a white LED source from a Luxeon Rebel (SP-12-W5, cool white). EIS was performed using a single sine wave and a perturbation voltage of *V*_rms_ = 7 mV. With logarithmic spacing, 70 frequency points between 1 MHz and 0.2 Hz were probed at each bias potential. Bias potentials were applied in 50 mV steps. The actual EIS measurement started 30 s after the application of the bias potential. Nyquist plots were fitted with ZView software from Scribner.

## Supplementary Material



## Footnotes

a We note that some electrochemical “DC” measurements such as cyclic voltammetry (CV) are actually time-dependent measurements since a capacitor like the Helmholtz capacitance will respond as a function of the scan velocity. This principle is used, for example, to measure the electrochemically active surface area by the determination of the double-layer capacitance or the diffusion coefficient of redox-active species by the scan velocity-dependent redox peak currents.^20,21^

b If the density of states of the investigated semiconductor is known, the energetic position of the Fermi level relative to the conduction and valence band edges can be determined. This information is particularly valuable for semiconductor/electrolyte interfaces. When combined with the band gap of the material obtained, for example, from optical measurements such as diffuse reflectance spectroscopy, the established Fermi level position allows construction of the complete energy-band diagram of the PEC system.^43,44^

## Notes

Information box 1: checklist for reliable PEC-EIS data acquisition; information box 2: analysis scheme for EIS data; series connection of *R* and *C* in the hydraulic analogy; AC voltage perturbation and Euler’s formula; circuit laws and impedance expressions; common standard EC models; representation of the measured EIS data; tips for fitting EIS data from a CPE to a capacitor; ECs used to fit EIS data; Butler–Volmer equation and detailed treatment of EIS of a multicrystalline platinum electrode; Nyquist plots of the measured Pt electrolyte solution interface; contact formation at a p–n diode; *J*–*V* curve of a solar cell, diode EC model, and EIS results; extended diode equation; Nyquist and Bode plots of the silicon solar cell; Mott–Schottky plots; contact formation in a buried-junction PEC device; measured EIS of p-Si/n-TiO_2_/Pt in 1 M H_2_SO_4_ at 10% sun and in the dark; three different light intensities of p-Si/n-TiO_2_/Pt; implications of the space–charge capacitance before the photocurrent onset and dual working electrode measurement; and Mott–Schottky plots of the PEC electrode (PDF)
