# Supplementary material for: Electrochemical Impedance Spectroscopy of (Photo)electrochemical Systems: A Practical Guide
Source: ACS Meas Sci Au. 2026 Aug 7;6(5):1433–44. doi: 10.1021/acsmeasuresciau.6c00109 (PMC13693279; doi:10.1021/acsmeasuresciau.6c00109)
Supplement: Supplementary file 1 [file tg6c00109_si_001.pdf]

Supporting Information

Electrochemical Impedance Spectroscopy of (Photo)electrochemical Systems: A Practical Guide

Thomas Moehl,*[a] Erin Service,[a] Wooseok Yang,[b,c] S. David Tilley*[a]

[a] Department of Chemistry, University of Zurich, Winterthurerstrasse 190, 8057 Zurich, Switzerland

[b] School of Chemical Engineering, Sungkyunkwan University (SKKU), Suwon 16419, Republic of Korea

[c]SKKU Institute of Energy Science and Technology (SIEST), Sungkyunkwan University, Suwon 16419, Republic of Korea

Content

Content ... 2
Information Box 1: Checklist for reliable PEC-EIS data acquisition ... 3
Information Box 2: Analysis scheme for EIS data ... 5
Supplementary Note 1: Series connection of R and C in the hydraulic analogy ... 6
Supplementary Note 2: AC voltage perturbation and Euler's formula[1,2] ... 7
Supplementary Note 3: Circuit Laws and Impedance Expressions ... 10
Supplementary Note 4: Common standard EC models ... 11
Supplementary Note 5: Representing the measured EIS data ... 12
Supplementary Note 6: Tips for fitting EIS data ... 16
Supplementary Note 7: From a Constant Phase Element (CPE) to a capacitor ... 18
Supplementary Note 8: The equivalent circuits used to fit EIS data ... 20
Supplementary Note 9: Butler–Volmer equation and detailed treatment of the EIS of a multicrystalline platinum electrode ... 21
Supplementary Note 10: Nyquist plots of the measured Pt electrolyte solution interface ... 24
Supplementary Note 11: Contact formation at a p–n diode ... 25
Supplementary Note 12: *J–V* curve of a solar cell, diode EC model and EIS results ... 27
Supplementary Note 13: Extended Diode Equation ... 30
Supplementary Note 14: Nyquist and Bode plots of the silicon solar cell ... 31
Supplementary Note 15: Mott–Schottky plots ... 32
Supplementary Note 16: Contact Formation in a Buried Junction PEC device ... 34
Supplementary Note 17: The measured EIS of a p-Si/n-TiO_2/Pt in 1 M H_2SO_4 at 10% sun illumination by white-light LED ... 36
Supplementary Note 18: Three different light intensities of a p-Si/n-TiO_2/Pt ... 38
Supplementary Note 19: Implications of the C_{SC} before the photocurrent onset and Dual Working Electrode Measurement ... 39
Supplementary Note 20: Mott–Schottky plots of the PEC electrode ... 42
Glossary ... 43
References ... 47

Information Box 1: Checklist for reliable PEC-EIS data acquisition

To perform the EIS measurements, several important preconditions (e.g., stability, linearity) must be met.[1–3] Especially in PEC devices, where corrosive side reactions are nearly always a challenge, there must be a balance between the stability of the measurement and the stability of the measured system.

- Small-signal perturbation (linearity):
  The sinusoidal voltage perturbation applied around the desired DC bias should be small, typically in the range of V_{pp} =5–10 mV. Impedance theory assumes linearity of the current–voltage relation, which is only valid for small perturbations (see Supplementary Notes 2 and 3). For example, if the current depends exponentially on voltage, Ohm's law (linear relation of current and voltage) is only applicable for sufficiently small, applied voltage perturbations.
- AC voltage definitions:
  For applying an acceptable voltage perturbation, the user also has to be familiar with how the amplitude of an AC signal is described:
  - V_{pp} – Peak-to-Peak Voltage – describes the total swing of an AC signal with:
    $$V_{pp} = V_{max} - V_{min} = 2 \cdot V_{peak}$$
  - V_{rms} – Root Mean Square Voltage – describes the DC equivalent that would deliver the same average power to a resistor as the AC waveform does:
    $$V_{rms} = \frac{V_{peak}}{\sqrt{2}} = \frac{V_{pp}}{2\sqrt{2}} \approx 0.3535 \cdot V_{pp}$$
- Measurement duration and stabilization
  Be aware of the total time required for an EIS measurement. With 60–70 frequencies ranging from 1 MHz down to 0.2–0.1 Hz, a single spectrum at one bias potential typically can take up to 1 minute. Additionally, a stabilization time of 10–40 s at each bias potential is crucial to ensure steady-state conditions before acquiring the spectrum (see Supplementary Note 6).
  Typical conditions:
  15 bias potential steps (e.g., 50 mV steps over ~800 mV of bias potential)
  30 s stabilization time per bias potential step
  This results in approximately 1.5 min per potential step and >20 min per full scan. Performing both dark and illuminated measurements can easily exceed 45 min overall, which can challenge device stability.
- Verification of stability:
  To verify system stability, perform cyclic voltammetry (CV) scans over the same potential range before and after the EIS measurement (two to three scans each). Significant deviations between the CVs before and after the EIS measurement indicate electrode degradation or instability.
- Reproducibility:
  Measure at least two nominally identical samples (no differences in fabrication parameters). If the EIS spectra are reproducible, the integrity of the data can be considered reliable.
- Extraction of the DC *J–V* curve:
  The DC *J–V* curve of the measured system can be extracted directly from the EIS data by plotting the current at the lowest frequencies (≤ 1 Hz) against the applied bias potential. This yields a stepped steady-state *J–V* curve.

- Light source considerations:
  White-light LEDs are preferred over Xe arc lamps in EIS measurements. LEDs provide continuous illumination, whereas pulsed light sources can introduce noise, particularly at low frequencies. Furthermore, the LED light intensity can be easily controlled via the driving current or voltage as compared to the arc lamps (where one should use metal meshes for reducing the light intensity). Reducing light intensity is often essential in PEC-EIS measurements, as gas bubble formation at high light intensities can introduce significant noise in the EIS data. For example, a single data point measurement at 0.2 Hz requires at least 5 s, which is sufficient for bubble formation and/ or detachment. Therefore, PEC devices are often measured at ~10% sun equivalent using white LEDs.
  LEDs typically exhibit a spectral mismatch compared to the solar spectrum or standard AM1.5G. If this leads to unrealistically low photocurrents, the LED intensity can be adjusted to match the current density obtained under Xenon AM1.5G lamp illumination.
- Counter electrode configuration:
  During EIS measurements, the counter electrode should be placed in the same electrolyte solution but separated by a frit to prevent products of the counter reaction from contaminating the working electrode compartment.
- Comparison with literature:
  Caution should be exercised when directly comparing EIS data with values reported in the literature. Variations in chemical purity, processing conditions, electrode preparation, and operator handling can significantly affect results. Literature comparison should focus on trends, not absolute values.
- Comparative device studies:
  For meaningful conclusions, comparative studies are highly recommended. The different devices should differ only by a single, well-defined modification. Comparing the EIS responses to a reference device greatly facilitates identifying and interpreting changes.
- Potential-dependent EC validity:
  Be aware that different applied bias potentials may activate or suppress different physical or electrochemical processes. Consequently, the appropriate EC model may change with potential regions. For example, dye-sensitized solar cells may require three or more distinct ECs over a potential range of ~700 mV.[4]

Typical measurement conditions for PEC devices:

Scanned potential range: generally, from low current to the voltage range with higher current;
start of the EIS measurement is at least 100 to 200 mV before photocurrent onset.
Potential steps: 50 to 25 mV (the more steps the better for subsequent data analysis but increasing the measurement time may challenge the stability of the sample).
Stabilization time at each potential: 30 s as a rule of thumb but clearly depends on the sample.
Frequency range: 1 MHz down to 0.2 or 0.1 Hz.
Voltage perturbation on applied DC potential: $V_{pp} < 20$ mV so about $V_{rms} < 7$ mV.

Information Box 2: Analysis scheme for EIS data

1. Study the known literature especially regarding the EC used and why it has been used. Employing an established EC model is not necessarily easy but it facilitates the analysis, while creating a new EC model for a novel system can be challenging, especially for devices with multiple interfaces and materials.
2. Perform the measurements.
3. Extract the DC current from the lowest measured frequency.
4. Take the derivative of the applied voltage with respect to the current to determine the total resistance during the measurement.
5. Examine the current–voltage characteristics (also in logarithmic plot). They can give information on different potential regions like forward or reverse bias or before and after hydrogen evolution onset.
6. Check in the Nyquist and Bode plots how many time constants are observable.
7. Check how the data in the EIS plots change with applied DC potential.
8. If there is an equivalent circuit known, crosscheck if the literature EC matches the elements in the observed EIS data. Be aware that certain EC models belong to a certain applied potential range and change when entering a new potential region.
9. Estimate the series resistance at high frequencies and determine the other resistances from the x-axis of the semicircle(s).
10. Build your model according to literature or, if the model is unknown, you can start with $(RC)_p$ elements in series in combination with a resistance accounting for the series resistance from cables or electrolyte solution.
11. Try fitting with capacitor elements. If the quality of the fitting is not satisfactory replace the capacitors with constant phase elements. Be aware that the exponent (phase factor) of the CPE should not go below 0.8.
12. Convert the CPE values into their equivalent capacitance (see Supplementary Note 7).
13. Plot the resulting data from the fitting against the applied DC potential. Since the changes of resistance can be over several orders of magnitude, logarithmic representation helps in visualizing the presented data.

Supplementary Note 1: Series connection of R and C in the hydraulic analogy

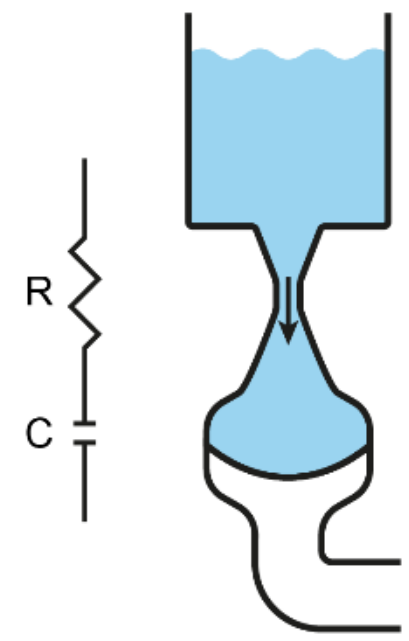

Figure S1. Series connection of a resistor and capacitor. Under series connection, no water can flow through the tube. The same applies to an ideal electrical circuit with a resistor and capacitor in series.

The series connection of a resistor and a capacitor yields zero current under DC conditions in the ideal case. Upon application of a DC potential, the capacitor charges with a time dependence governed by the RC time constant, which is limited by the resistance of the connecting pathway. The corresponding electrochemical analogy is underpotential hydrogen deposition on Pt or charge-compensated ionic adsorption at an electrode surface, where charge accumulation occurs without faradaic current.[5]

Supplementary Note 2: AC voltage perturbation and Euler's formula[1,2]

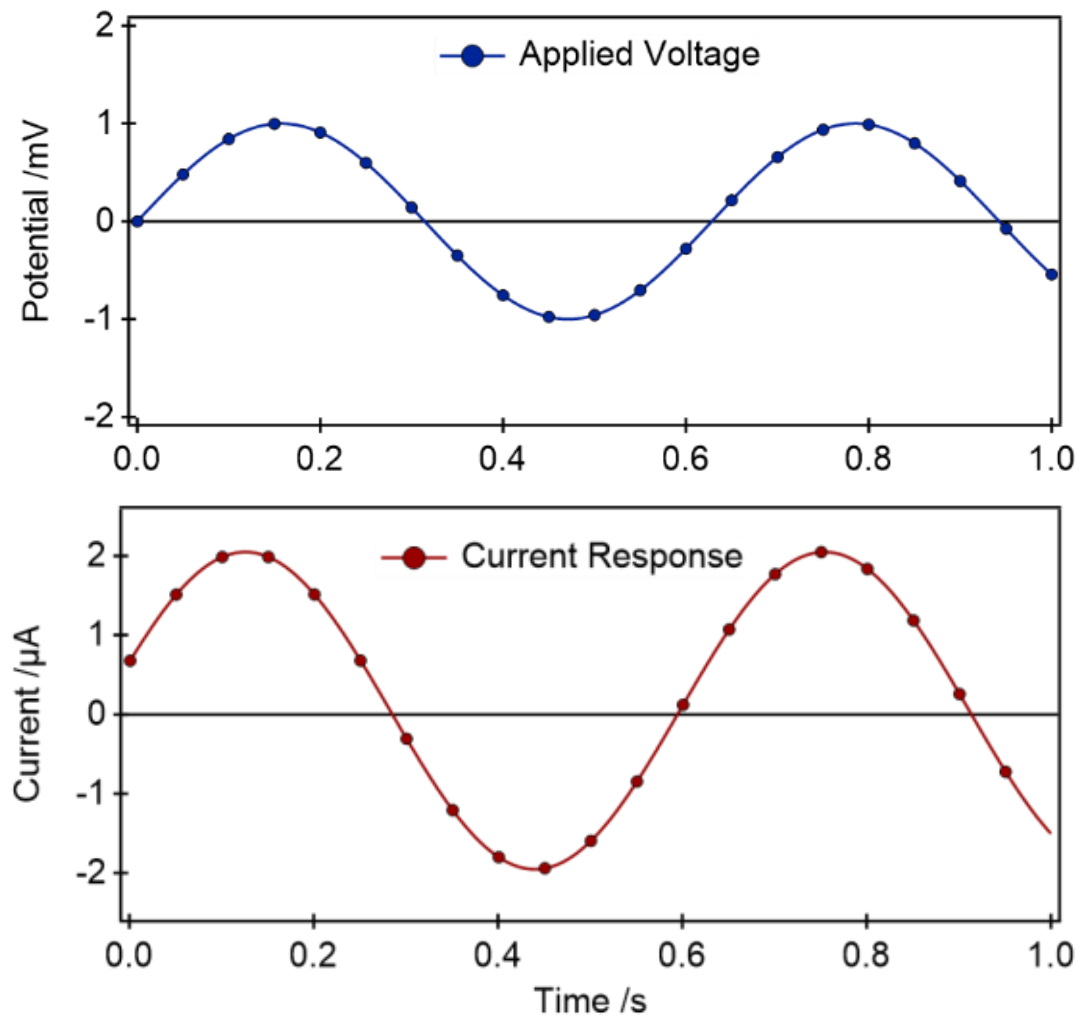

$$\mathrm{v(t)} = \mathrm{V}_0 e^{i\omega t}$$

$$\mathrm{i(t)} = \mathrm{I}_0 e^{i(\omega t+\phi)}$$

Figure S2. Current response to a sinusoidal voltage perturbation (f ≈ 1.67 Hz). V_0 and I_0 represent the voltage or current amplitude; i is the imaginary unit; ω the angular frequency; t the time and ϕ the phase shift (for a more detailed explanation see below).

Electrochemical impedance spectroscopy data is represented in multiple coordinate systems because each form emphasizes different aspects of the complex impedance. The Cartesian (rectangular or real–imaginary) form clearly shows the individual resistive and reactive (phase-shifted) contributions, making it easier to analyze circuit components. Polar, magnitude–phase, and exponential forms emphasize the overall impedance magnitude and phase angle, which are often more intuitive for interpreting frequency-dependent behavior, phase shifts, and system dynamics (see Table S1 at the end of the section). A detailed and thorough treatment of the mathematical description of EIS data can be found in the manuscript of Lazanas et *al.*[2] and others.[3]

All formulas presented here are based on the engineering/ electrochemistry convention (in contrast to the convention in physics and optics). A small sinusoidal voltage perturbation is applied to the device, and the resulting current response is recorded. An impedance measurement quantifies how strongly a system opposes the flow of alternating current as a function of angular frequency $\omega = 2\pi f$. The impedance $Z(\omega)$ is expressed as

$$Z(\omega)=R(\omega)+iX(\omega),$$

where R is defined as the real *in-phase* component (resistance), X as the *out-of-phase* component (reactance) and *i* is the imaginary unit.

Using Euler's formula, $e^{i\phi}=\cos(\phi) + i\sin(\phi)$, AC signals can be represented as complex numbers, with ϕ being the phase shift between voltage and current. When a sinusoidal voltage

$$v(t)= V_0 \cos(\omega t) = \Re\{\tilde{V} e^{i\omega t}\}$$

is applied, the resulting current

$$i(t) = I_0 \cos(\omega t + \phi) = \Re\{\tilde{I} e^{i\omega t}\}$$

can show a phase shift (ϕ), for example in the presence of capacitive elements (V_0 and I_0 represent the voltage or current amplitude around a bias voltage or current). The last part of the formula represents the real part of voltage and current in phasor notation. A common point of confusion is that although we represent signals using complex exponentials, the physically measured voltage and current are always real. One can measure it with a voltmeter or ammeter, and it never has an imaginary component (although the current might contain a phase-shifted contribution from e.g. a capacitor).

The impedance is defined as the ratio of the voltage and current phasors $\tilde{V}$ and $\tilde{I}$ (not the instantaneous time-domain values, which would vary with t):[1,2]

$$Z(\omega) = \frac{\tilde{V}}{\tilde{I}} = \frac{|V|}{|I|e^{i\phi}} = \frac{|V|}{|I|} e^{-i\phi} = |Z|e^{-i\phi}$$

Using Euler's formula, the impedance can be written in rectangular (Cartesian) form as

$$Z(\omega) = |Z|(\cos(\phi) - i\sin(\phi)) = Z'(\omega) + iZ''(\omega) = R + iX$$

where

$$Z'(\omega) = R = |Z|\cos(\phi)$$

$$Z''(\omega) = X = -|Z|\sin(\phi)$$

where $Z'(\omega)$ and $Z''(\omega)$ denote the real and imaginary components of the impedance, respectively. X, as mentioned above, denotes the reactance, which takes negative values for capacitive elements and positive values for inductive elements.

The components of $Z'(\omega)$ and $Z''(\omega)$ are related to the magnitude and phase as defined by the other coordinate systems by

$$|Z| = \sqrt{[Z'(\omega)]^2 + [Z''(\omega)]^2} \text{ and } \phi = \arctan\left(\frac{Z''(\omega)}{Z'(\omega)}\right)$$

Table S1: The different coordinate systems used in impedance.

**Coordinate System**	**Name**	**Mathematical Form**	**Component Relations**
Cartesian (x–y plane)	Rectangular	$Z = Z' + iZ''$	$Z' = \mathrm{Re}(Z)$,<br>$Z'' = \mathrm{Im}(Z)$
Polar (r–φ)	Trigonometric	$Z = \lvert Z\rvert(\cos\phi - i\sin\phi)$	$\lvert Z\rvert = \sqrt{Z'^2 + Z''^2}$<br>$\phi = \arctan\left(\frac{Z''}{Z'}\right)$
Polar (r–φ)	Magnitude–Phase (Phasor)	$Z = \lvert Z\rvert\angle\phi$	$\lvert Z\rvert = \sqrt{Z'^2 + Z''^2}$,<br>$\phi = \arctan\left(\frac{Z''}{Z'}\right)$
Exponential (complex)	Euler	$Z = \lvert Z\rvert e^{i\phi}$	$\lvert Z\rvert = \sqrt{Z'^2 + Z''^2}$,<br>$\phi = \arctan\left(\frac{Z''}{Z'}\right)$

Supplementary Note 3: Circuit Laws and Impedance Expressions

(1) Resistances in series: $R_{total} = R_1 + R_2 + R_3$

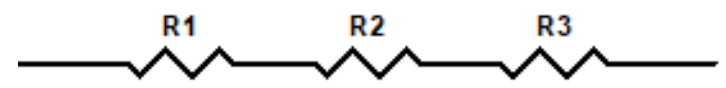

(2) Resistances in parallel: $\frac{1}{R_{total}} = \frac{1}{R_1} + \frac{1}{R_2} + \frac{1}{R_3}$

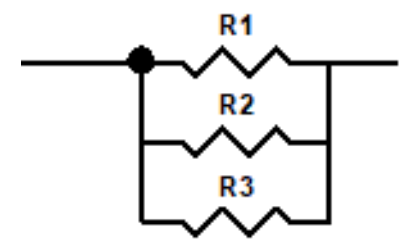

(3) Capacitances in series: $\frac{1}{C_{total}} = \frac{1}{C_1} + \frac{1}{C_2} + \frac{1}{C_3}$

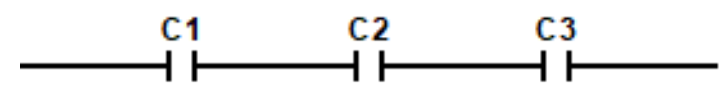

(4) Capacitances in parallel: $C_{total} = C_1 + C_2 + C_3$

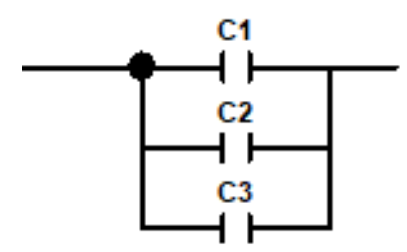

We can sum up impedances just like resistances in a DC circuit.

(5) Impedance in series: $Z_{total} = Z_1 + Z_2 + Z_3$

(6) Impedance in parallel: $\frac{1}{Z_{total}} = \frac{1}{Z_1} + \frac{1}{Z_2} + \frac{1}{Z_3}$

(7) Impedance of a resistor: $Z_R = R$

(8) Impedance of a capacitor: $Z_C = iX = \frac{1}{i\omega C} = \frac{-i}{\omega C}$

(9) Impedance of a CPE (Constant Phase Element): $Z_{CPE} = \frac{1}{(i\omega)^n Q}$; with Q as a measure of the magnitude of the CPE, ω is the angular frequency (2πf), and n (0 ≤ n ≤ 1) is the so-called phase factor.

(10) Impedance of the reduced Randles circuit (without the Warburg diffusion element) at a metal/ electrolyte solution junction (with the Helmholtz capacitance and the charge-transfer resistance, C_{Helm} and R_{CT}):

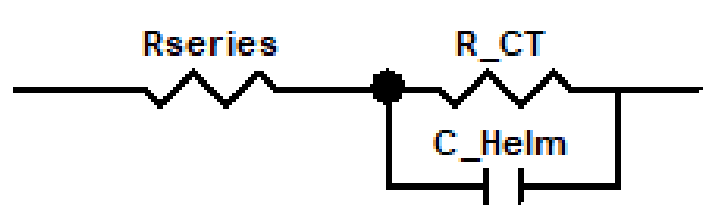

$$Z_{Randles} = R_{series} + \frac{Z_{C_{Helm}} Z_{R_{CT}}}{Z_{C_{Helm}} + Z_{R_{CT}}} = R_{series} + \frac{\frac{R_{CT}}{i\omega C_{Helm}}}{R_{CT} + \frac{1}{i\omega C_{Helm}}} = R_{series} + \frac{R_{CT}}{1 + i\omega R_{CT} C_{Helm}}$$

Supplementary Note 4: Common standard EC models

Reduced Randles equivalent circuit model (with charge-transfer resistance and Helmholtz capacitance):

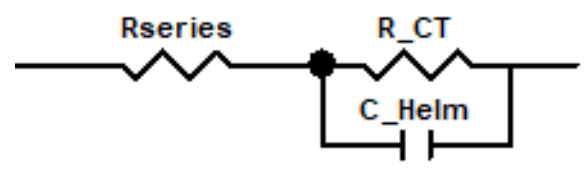

The extended Randles circuit includes an additional diffusion element (a Warburg impedance) and replaces the ideal capacitance with a constant phase element (CPE) to account for non-ideal capacitive behavior arising from surface roughness, heterogeneity, or distributed time constants:

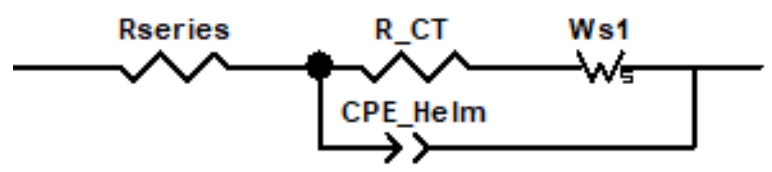

The three simplest EC models for two semicircles in the Nyquist plot (all with CPEs instead of ideal parallel plate capacitors):

a) Voigt EC: The two resistive–capacitive elements operate independently of each other. Each resistance is associated with its own time constant, and both resistances can be read directly from the diameters of the corresponding semicircles in the Nyquist plot.

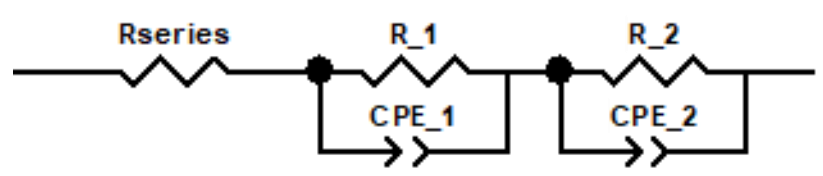

b) Maxwell (Dielectric) EC (a parallel RC element in parallel with a series RC element): One resistance does not directly conduct current but still influences the overall impedance response by modulating the charging behavior of the system. In the present context, this configuration represents charge transfer that is coupled to a charged interfacial layer. In contrast to the Voigt circuit, resistance values cannot be read directly from the Nyquist plot.

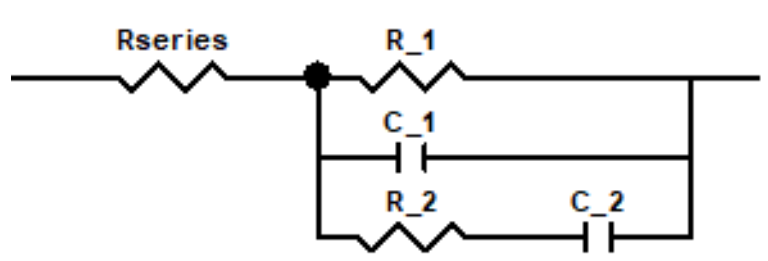

c) Nested EC (also called Matryoshka EC): Commonly used in photoelectrochemical systems, this model describes a single charge-transfer process that is associated with two distinct capacitances, often attributed to charge transfer through surface or defect states.

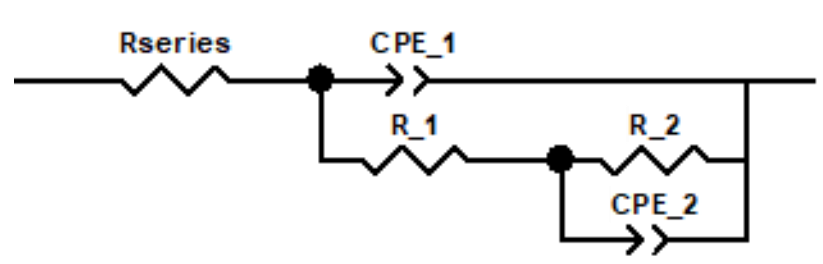

While the resistance values of the Voigt and nested ECs can be extracted directly from the Nyquist plot, the values for the Maxwell circuit must be extracted through fitting. Impedance data can be mathematically transformed between these different EC representations, yielding equivalent physical information.[6,7]

Supplementary Note 5: Representing the measured EIS data

The two most common ways to present EIS data are the **Bode plot** and the **Nyquist plot**. The Bode plot provides a frequency-dependent representation of the impedance magnitude (or modulus) and phase angle, whereas the Nyquist plot displays the imaginary part of the impedance as a function of its real part.

For many researchers, the Nyquist plot offers a more intuitive visualization of impedance data, as it allows different electrochemical processes to be readily identified and separated by the appearance of semicircles. The diameters of these semicircles often correspond to resistance values, which can usually be obtained by extrapolation to the x-axis, Fig. S3. Note that this does not apply when using a Maxwell equivalent circuit. In addition to semicircular features, the Nyquist plot can also reveal charge transport or diffusion-related processes, which typically appear as straight lines with a slope of approximately 45°, characteristic of diffusion-controlled impedance behavior.

Nyquist Plot in EIS

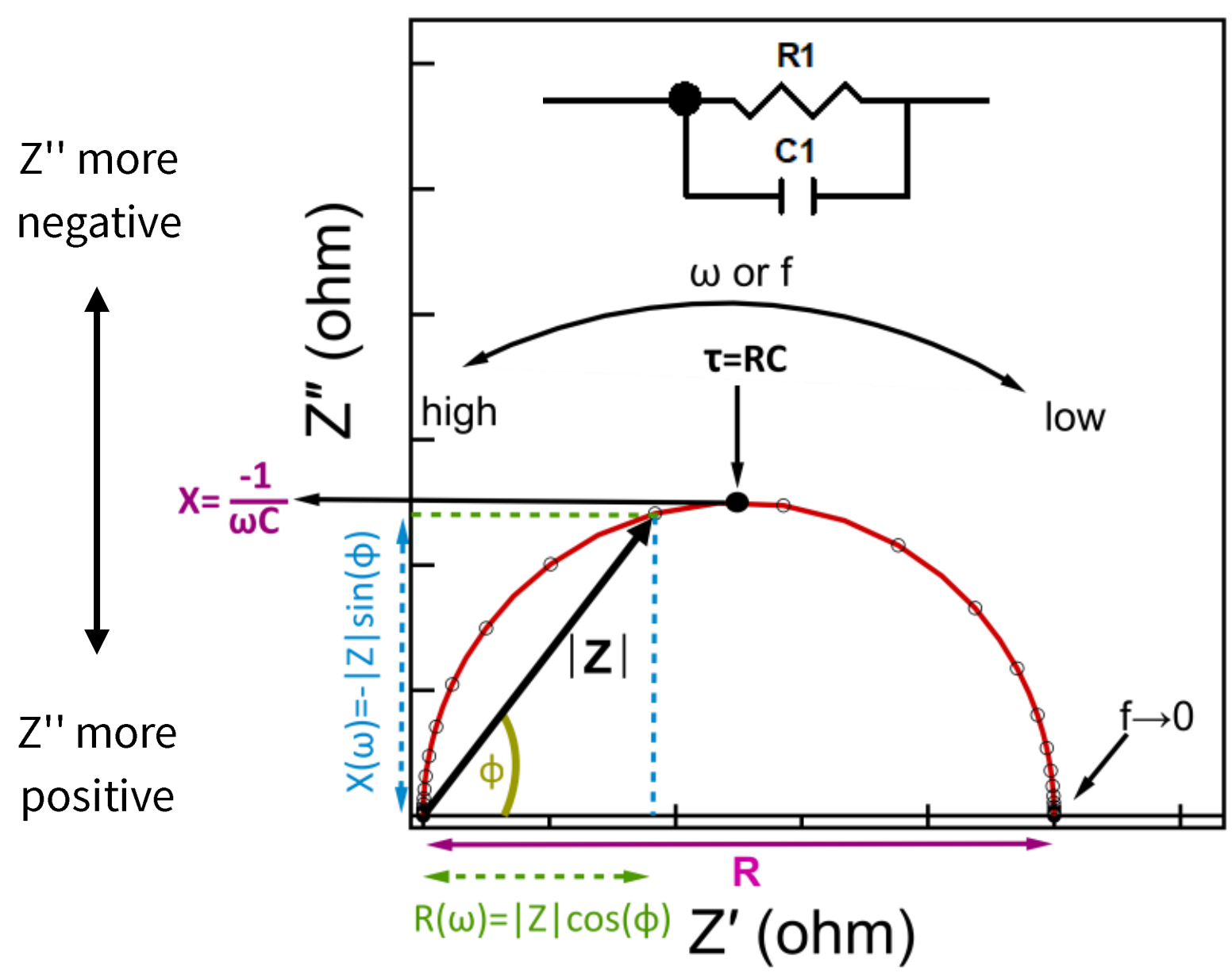

Figure S3. Nyquist representation of the EIS data. Note that Z'' is displayed in the positive quadrant, although it corresponds to increasingly negative values. Each point on the semicircle is determined by the vector of modulus | Z | at angle ϕ from the origin of the coordinate system. This vector defines the real and imaginary parts on the x- and y-axes.

The plot is a complex plane plot with the real part of the impedance (Z') on the x-axis and the imaginary part of the impedance (Z'') on the y-axis. Some authors define the y-axis as –Z'' and plot positive values (Fig. S3).

The data is plotted as a series of points representing different frequencies, typically starting from high (left) to low frequencies (right). Z'' is displayed in the positive quadrant, although it corresponds to increasingly negative values (Z'' is also often plotted in the literature as –Z'' with increasingly positive values).

For a simple parallel RC element, the resistance value can be determined at the low-frequency limit, where the semicircle intercepts the real (x) axis in the Nyquist plot, corresponding to the diameter of the semicircle. The low-frequency limit essentially represents DC conditions and therefore reflects the overall resistance of the system derived from the DC current response. Again, this direct determination of resistance from the real axis applies to simple equivalent circuits but does not hold for the Maxwell equivalent circuit, where the resistive elements are coupled differently and cannot be read directly from the Nyquist plot.

The imaginary part of the impedance represents the reactance X, which originates from capacitive (or inductive) behavior. This reactance can be converted into a capacitance, as described in Supplementary Note 3.

Each point in a Nyquist plot corresponds to a specific frequency and is defined by the impedance magnitude |Z| and the phase angle ϕ, representing the real and imaginary components of the system's response at that frequency.

The overall shape of the Nyquist plot provides insight into the underlying electrochemical or electronic processes. Semicircles commonly indicate charge-transfer resistance in parallel with double-layer (or Helmholtz) capacitance at electrochemical interfaces. In photodiodes or solar cells, a semicircle typically represents the diode resistance in parallel with the space-charge capacitance.

Straight 45° lines at low frequencies might indicate diffusion processes in the electrolyte solution (Warburg impedance)[1,2] or charge transport limitations in (disordered) materials.[4]

Bode Plots in EIS

A Bode plot in EIS provides a frequency-dependent visualization of the impedance of an electrochemical system by plotting both the impedance modulus (also referred to as magnitude) and the phase information as a function of frequency (Fig. S4).

Figure S4 presents the magnitude and phase plots for a simple parallel RC element. Three distinct frequency regions can be identified. At high frequency, the response is dominated by the capacitive element, as evidenced by an approximately -90° phase shift between current and voltage (see also Supplementary Note 2). At low frequencies, the response is governed by the resistive element and corresponds to the DC current of the investigated system. In the intermediate frequency range, a mixed response of the resistor and capacitor is observed.

The inflection point in the phase plot, occurring at a characteristic frequency (in the range of 10–20 Hz for this example), corresponds to the characteristic frequency of the RC element and coincides with the maximum of the semicircle in the corresponding Nyquist plot shown in Fig. S4.

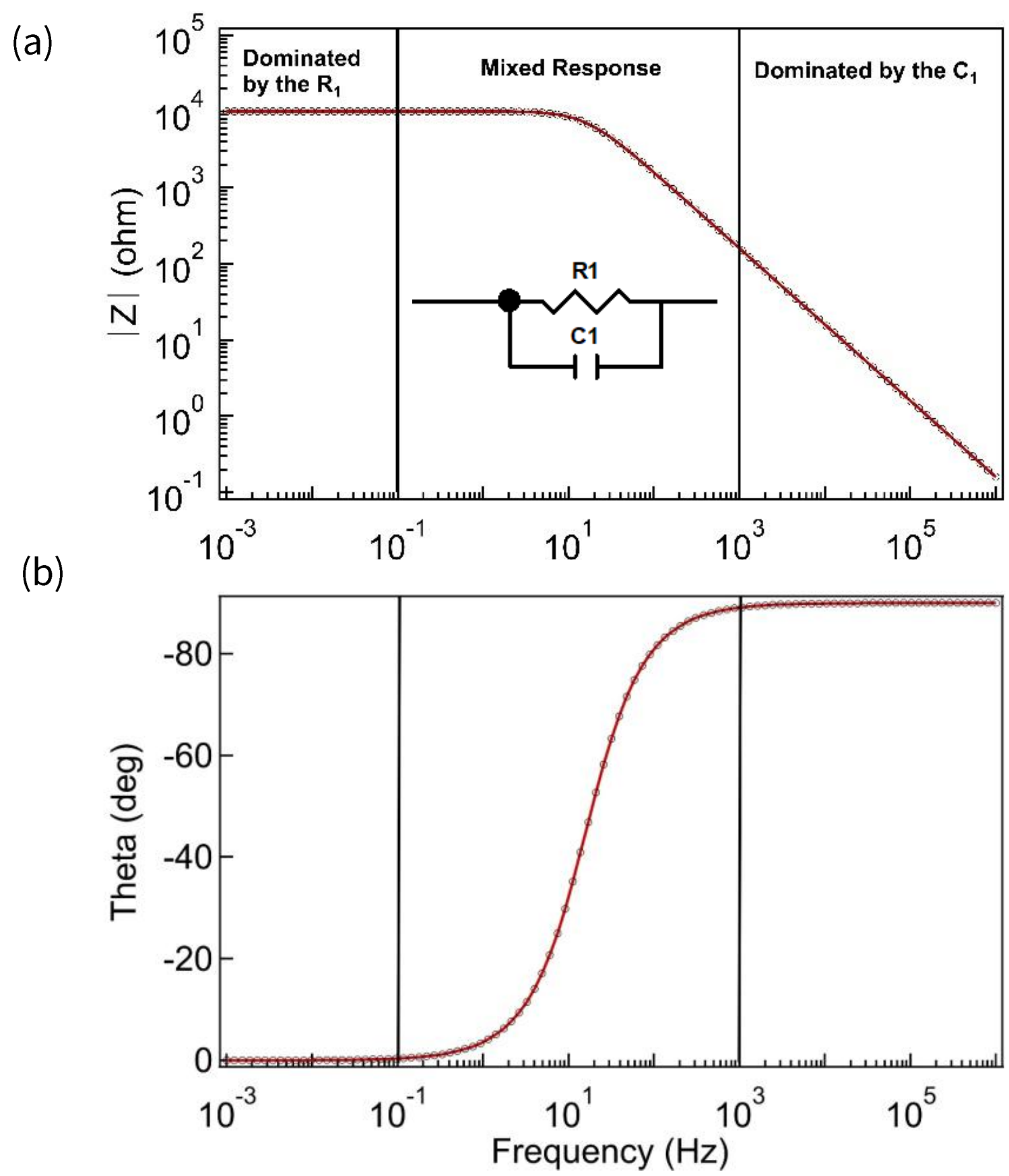

Figure S4. (a) Bode plots of | Z | vs frequency of the potential perturbation (log scale). (b) Phase shift vs frequency. Parameters: $R_1 = 10^4$ ohm and $C_1 = 1 \cdot 10^{-6}$ F

How would the different EC elements appear in both Nyquist and Bode plots?

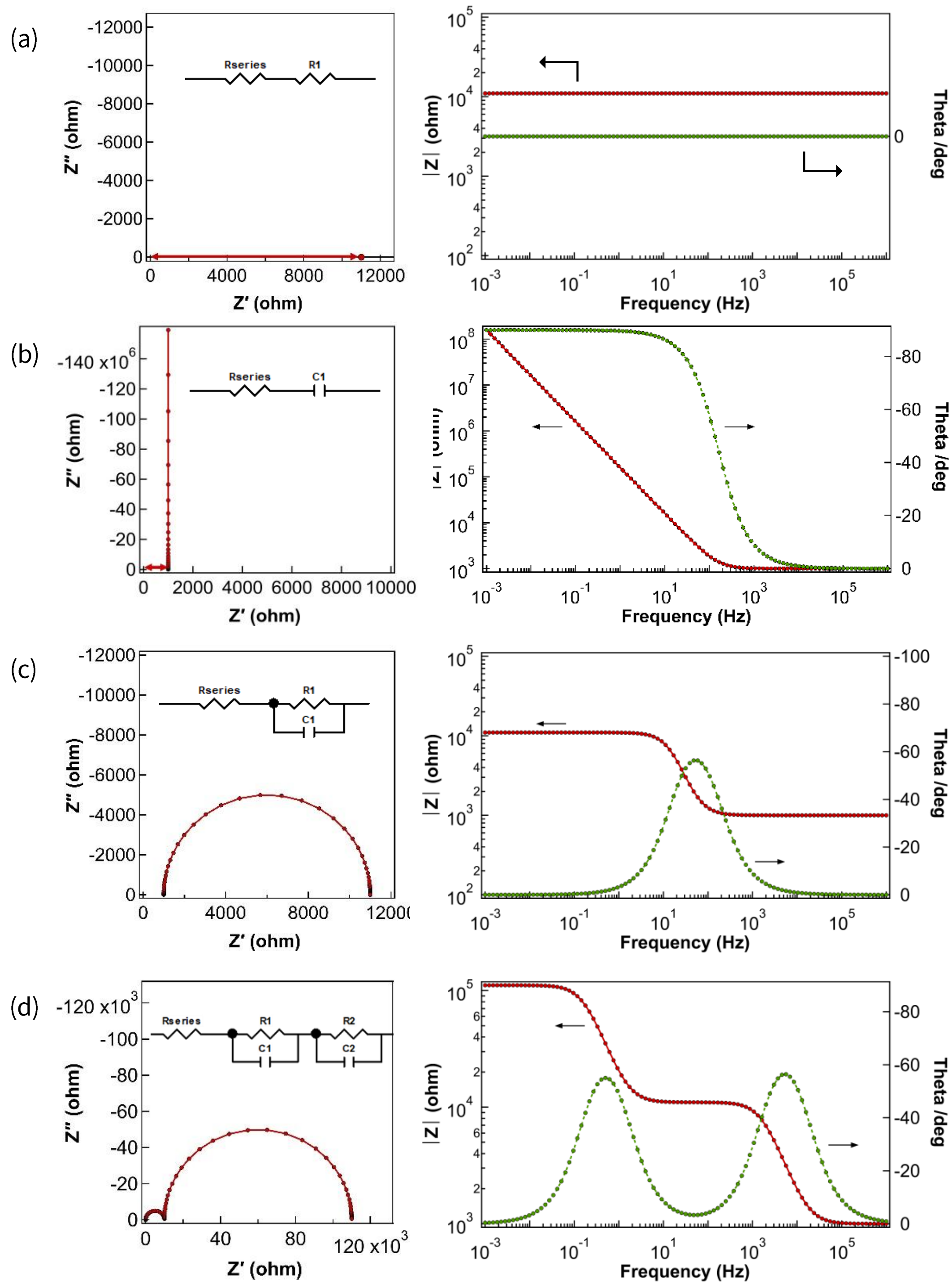

Figure S5. Nyquist (left) and Bode plot (right) of (a) two resistances in series (R_{series}= 1•10^3 ohm and R1= 1•10^4 ohm). The Nyquist plot shows only one point at 1.1•10^4 ohm. (b) Series connection of a resistance and capacitance (R_{series} = 1•10^3 ohm and C1 = 1•10^{-6} F). (c) Series resistance and a resistance and capacitance connected in parallel (R_{series}= 1•10^3 ohm; R1 = 1•10^4 ohm and C1 = 1•10^{-6} F). (d) Series resistance with two $(RC)_p$ connected in series (R_{series} = 1•10^3 ohm; R1 = 1•10^4 ohm and C1 = 1•10^{-8} F; R2= 1•10^5 ohm and C2 = 1•10^{-6} F).

Supplementary Note 6: Tips for fitting EIS data

Most potentiostat software includes built-in functions for fitting impedance data. However, these tools can be cumbersome, especially when handling large datasets. Free software programs such as EIS-Spectrum-Analyzer (http://www.abc.chemistry.bsu.by/vi/analyser/) or MEISP (https://impedance0.tripod.com/) for Windows provide sufficient functionality for beginners. Commercial alternatives include ZView (Scribner) or RelaxIS (RHD Instruments).

EIS data is commonly fitted to an EC model using Complex Nonlinear Least Squares (CNLS) fitting, in which both the real and imaginary parts of the impedance are simultaneously minimized.[8–10] The algorithm most widely used for this purpose is the Levenberg–Marquardt Algorithm (LMA), which iteratively adjusts the model parameters to minimize the weighted sum of squared residuals between the measured and model-generated impedance across all frequencies. The LMA adaptively transitions between a gradient descent approach — which is robust when parameter estimates are far from their optimal values — and a Gauss–Newton approach — which converges rapidly in the vicinity of the solution.[11,12] In practice, the quality of a CNLS fit should always be assessed not only from the visual overlap of the fitted curve with the Nyquist and Bode plots, but also from the residuals of the real and imaginary components plotted as a function of frequency. A good fit is characterized by residuals that are small in magnitude and randomly distributed around zero, with no systematic trends. Systematic deviations in the residuals indicate that the chosen EC model does not adequately capture all physical processes present in the system, and a revised model should be considered. A good numerical fit does not guarantee meaningful results. The applied equivalent circuit must reflect the physical, photophysical, or electrochemical processes of the system, rather than being an arbitrary combination of resistors and capacitors.

If an EC has been established for a particular system, literature values can provide reasonable starting points for the fitting parameters, ensuring a stable and reliable fit. Pay special attention to the high-frequency intercept, which corresponds to the series resistance; an inaccurate value here can cause the fitting to fail.

For unexplored systems, the first step is to inspect the Nyquist and Bode plots to identify the number of distinct time constants. For instance, if two semicircles are visible, a good starting point is a Voigt model with two parallel RC elements in series. Capacitive elements can be replaced with a constant phase element if the fit is not initially satisfactory (see Supplementary Note 7). Data from a Voigt circuit can also be converted to Maxwell circuit parameters using standard equations.[13]

It is common for the EC to change across different potential regions. Therefore, analyze distinct potential regions separately, such as before and after the onset of (photo)current, to account for different processes taking place.

A frequent challenge for beginners is choosing initial values for resistances and capacitances. The series resistance can normally be read from the high frequency intercept in the Nyquist plot. For the other resistances and capacitances for sample areas of ~ 1 cm^2 practical starting points include:

- RC elements: resistance values can often be estimated from semicircle diameters. For incomplete low-frequency semicircles, a rough extrapolation even by eye is usually sufficient to estimate a reasonable starting value for a resistance.
- Capacitances: high-frequency space-charge capacitance can be around ~10^{-7} F, while low-frequency capacitances are typically ~10^{-5} F. If needed, replace capacitors with a CPE (exponent > 0.8) to improve fitting routine.

Finally, it is not recommended to initiate the fitting procedure using the first EIS spectrum recorded at the initial bias potential. As this represents the first application of an external potential to the device, the system generally exhibits the longest stabilization time and sometimes has not reached the steady-state condition yet (Fig. S6). Instead, fitting should start with EIS data obtained at the second or third bias potential step and then proceed backward to earlier potentials.

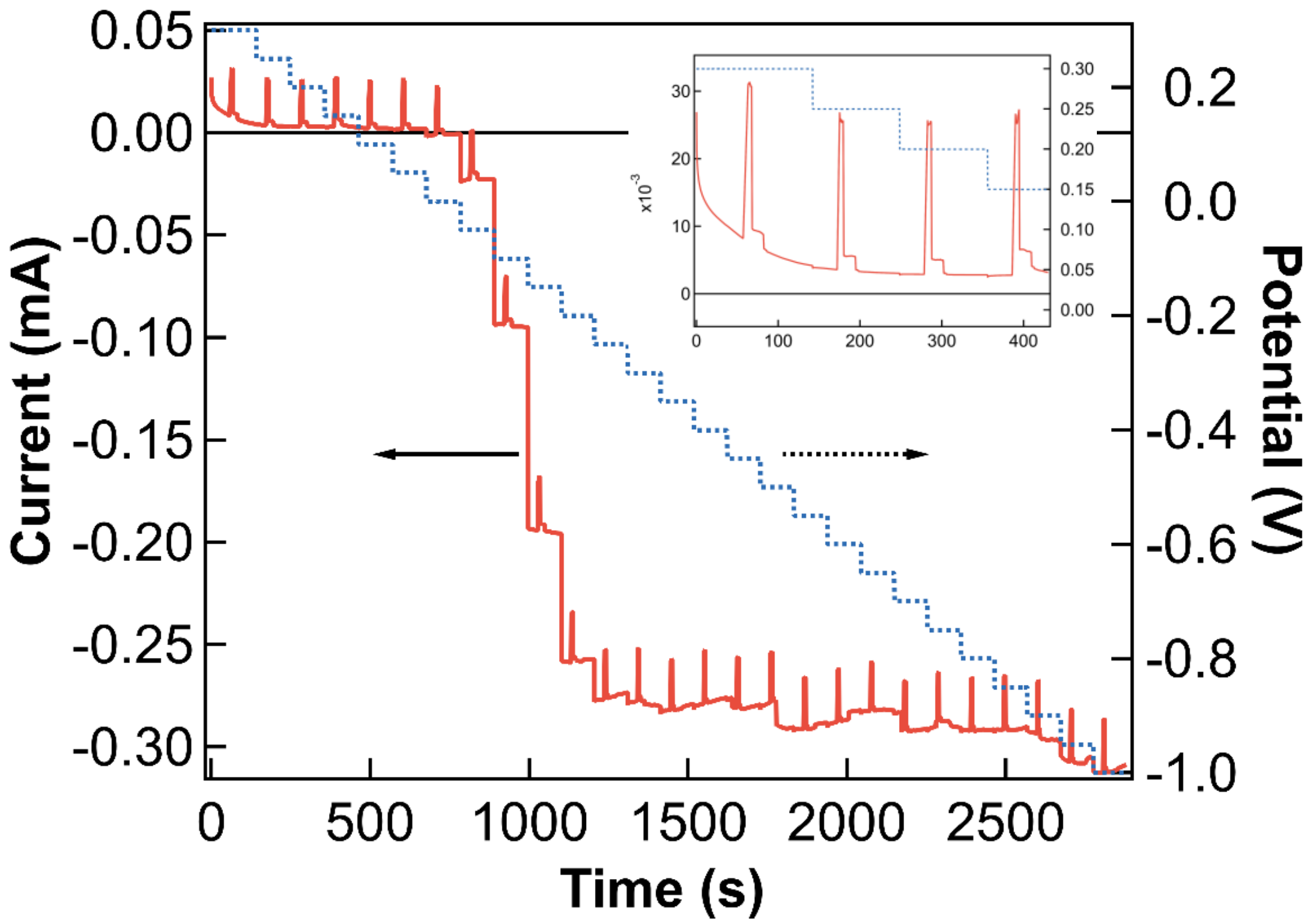

Figure S6. Current and potential during an EIS measurement plotted vs time. Especially at the first applied potential (see inset) the stabilization time can be much longer compared with the following potentials. Inset shows a magnification from the start of the measurement.

Supplementary Note 7: From a Constant Phase Element (CPE) to a capacitor

A capacitor (C) stores and releases charge (electric or ionic) and is characterized by a constant capacitance value and an impedance that decreases with increasing frequency (Fig. S5b), following $Z=1/(i\omega C)$.

A constant phase element (CPE), on the other hand, is a more generalized model used to describe non-ideal capacitive behavior observed in real systems, for example due to surface roughness, inhomogeneities, or distribution of time constants for the processes taking place at the surface or interface (*e.g.*, charge-transfer processes or recombination events).[14,15]

The impedance of a CPE is given by

$$Z(CPE) = \frac{1}{Q(i\omega)^n}$$

where Q is the CPE magnitude parameter (with units $F \cdot s^{n-1}$), ω is the angular frequency (2πf), and n ($0 \leq n \leq 1$) is the so-called phase factor. For n=1, the CPE reduces to an ideal capacitor with Q=C.

Unlike an ideal capacitor, a CPE can model a range of behaviors between a pure resistor (n=0), a Warburg like diffusion feature (n=0.5) and a pure capacitor (n=1). This flexibility makes CPEs valuable for accurately fitting impedance data of complex (photo)electrochemical systems. However, care must be taken when interpreting the phase factor n. In our fitting procedure, the CPE is used purely to mimic a capacitive element. The phase factor n should therefore be near 1 (with n=1 being an ideal parallel plate capacitor). One should first try to model the capacitances with an ideal parallel plate capacitor, though an ideal capacitor is often insufficient to describe real interfaces. Especially surface roughness can lead to a deviation from the phase factor from 1. As a rule of thumb, *the phase factor should remain above 0.8* if the CPE is still intended to represent a capacitance. If n falls below this value, the validity of the applied model should be carefully re-evaluated.

Because Q is not a capacitance, a conversion is needed to extract a physically meaningful effective capacitance value based on the extracted fitting data from the CPE.[16–20] The most common formula used is the Brug formula. For a CPE in parallel with a resistance R_p and a series resistance (see the reduced Randles circuit in Supplementary Note 4), the effective capacitance can be calculated as:

$$C = Q^{\frac{1}{n}}\left(R_{series}^{-1} + R_p^{-1}\right)^{\frac{(n-1)}{n}}$$

Formula	Expression	Circuit context	Parameters needed	Comments
**Brug formula[18,20] (Faradaic, general)**	$C_{eff} = Q^{1/n} \cdot \left(\frac{R_{series} R_p}{R_{series}+R_p}\right)^{(1-n)/n}$ $= Q^{\frac{1}{n}} \cdot \left(R_{series}^{-1} + R_p^{-1}\right)^{\frac{(n-1)}{n}}$	CPE in parallel with charge-transfer resistance (2D surface heterogeneity)	Q, n, R_{series}, R_p all from EC fit	Helmholtz or Space-charge capacitance
**Brug formula[18,20] (blocking electrodes)**	$C_{eff} \approx Q^{1/n} \cdot \left(R_{series}^{-1}\right)^{\frac{(n-1)}{n}}$	No Faradaic reaction at electrode	Q, n, R_{series}, all from EC fit	Helmholtz or Space-charge capacitance
**Hsu–Mansfeld formula[19,20]**	$C_{eff} = Q \cdot \omega_{max}^{(n-1)}$ with $\omega_{max} = 2\pi f_{max}$ where $-Z''$ is maximum	Any single, well-resolved (RC) feature with an identifiable −Z″ maximum (empirical, ω_max-based)	Q, n from EC fit; ω_{max} read from −Z″ peak in raw spectrum	Oxide or passive film when measured alone or with well separated time constants. Can only be applied if ω_{max} can unambiguously be identified.
It is an ongoing discussion in literature on how to convert CPEs into an effective capacitance. The interested reader is guided to the publication of Hirschorn *et al.* and others.[14,15,20,21]				

Supplementary Note 8: The equivalent circuits used to fit EIS data

Platinum electrolyte solution interface:

The equivalent circuit used depends upon the potential range investigated, as different processes occur at different potentials.

a) At positive potentials with anodic currents in the platinum oxide formation region:

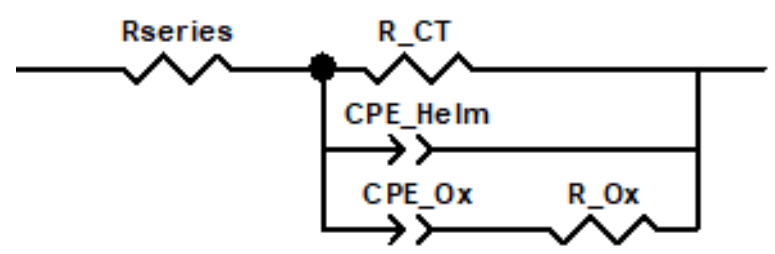

b) In the potential region approaching the onset of hydrogen evolution (UPD region):

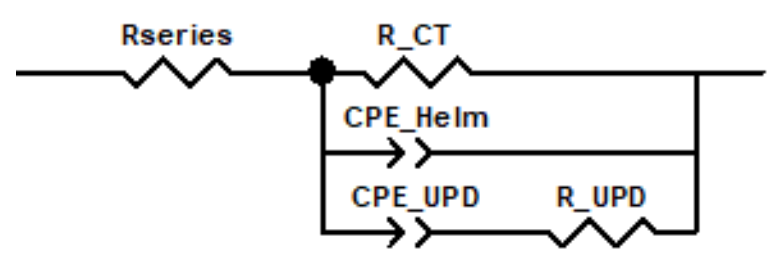

c) Potential region of hydrogen evolution with and without Warburg diffusion element:

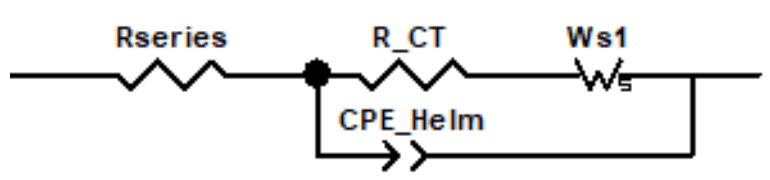

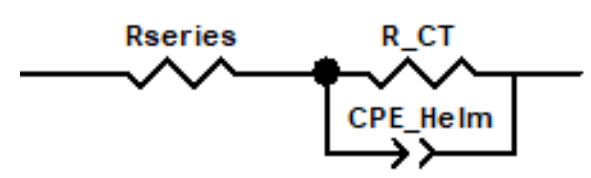

Silicon solar cell:

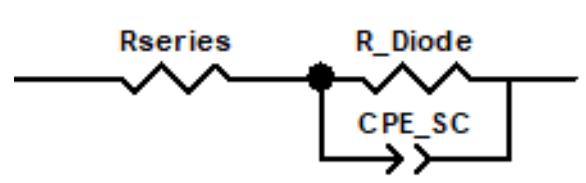

p-Si/a-TiO$_2$/Pt PEC photocathode:

a) Before the onset of photocurrent:

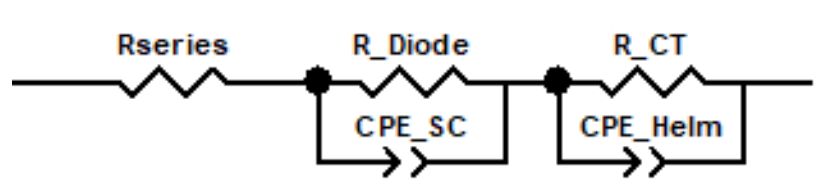

b) After the onset of photocurrent:

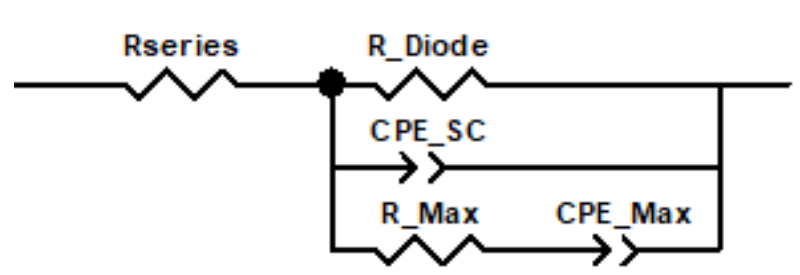

Supplementary Note 9: Butler–Volmer equation and detailed treatment of the EIS of a multicrystalline platinum electrode

The current through a metal–electrolyte solution interface can be described by the Butler–Volmer equation (BV). It relates the anodic and the cathodic current to the applied (over)potential:[22,23]

$$J_{Total} = J_{Anode} + J_{Cathode} = J_o * e^{\left(\frac{\alpha_A * z * F}{RT} * \eta\right)} - J_o * e^{\left(\frac{-\alpha_C * z * F}{RT} * \eta\right)} \qquad \text{(Eq. S1)}$$

with J_{Total}: overall current density; J_{Anode} and $J_{Cathode}$: anodic and cathodic current density; J_0: exchange current density; α_x: the charge-transfer coefficient for the anode and the cathode ($\alpha_A + \alpha_C = 1$ under ideal conditions); z: the number of electrons transferred; F: Faraday's constant; R: the universal gas constant; T: the temperature; η: the overpotential.

A simulation of the faradaic current using the Butler–Volmer equation with literature values for a multicrystalline Pt electrode (charge-transfer coefficient $\alpha_C = 0.5$, $\alpha_A = 0.001$ and exchange current density = $4.4 \cdot 10^{-4}$ A/cm^2) is presented in Fig. S7a together with its derivative of the voltage vs the current, which represents the total resistance of the simulated current voltage characteristics ($R_{Total} = \frac{dV}{dJ}$).[24,25] The asymmetry of the charge-transfer coefficient stems from the fact that the reduction product of this reaction, the hydrogen gas, is only present in low concentrations in solution.

The BV equation provides a useful first approximation; however, it is based on assumptions that neglect diffusion effects, capacitances, and multistep adsorption reactions.[23,26] Consequently, it cannot describe certain properties of platinum electrodes, such as behavior in the potential region of hydrogen underpotential deposition (UPD), which occurs at potentials more positive than the thermodynamic hydrogen evolution onset at 0 V vs RHE.

The example measurement of a metal–electrolyte solution interface, a polycrystalline Pt electrode in 1 M $HClO_4$, was also described in the main text (see Fig. 3) though only the hydrogen evolution region was addressed. Here we will take a look at additional potential regions like the UPD of hydrogen.

The DC current measured during the EIS experiment was first extracted and plotted as a function of the applied potential (Fig. S7b). In a second step, the derivative of the voltage with respect to the current density yielding the total resistance of the system was plotted versus the applied potential (Fig. S7c).

In addition to the hydrogen evolution region, other potential regions are discussed to demonstrate that even a seemingly straightforward and well-known system such as a Pt/$HClO_4$ interface can exhibit significant complexity. This complexity arises from multiple factors, including the type of Pt electrode (mono- or multicrystalline and dominant crystal facets), surface preparation (polishing, flame annealing), electrochemical pretreatment (e.g., prior *J*–*V* measurements), the applied potential window, pH, and even the nature of the acid counterion, all of which influence the measured *J*–*V* curve and, consequently, the EIS response.

The *J*–*V* curve in Fig. S7b shows three distinct regions. At positive potentials with anodic current platinum oxides are formed.[27,28] Below 0 V vs RHE the cathodic H_2 evolution is observed (this region can

be used to extract valid Tafel and BV parameters[29,30] if the iR drop correction has been applied to the data[31]). Between these potential regions, the double layer region and the region of UPD of hydrogen should be observed. The latter is in the potential region before the H_2 onset while the double layer region is near the potential of the platinum oxide formation. In our measurement though, the two potential regions cannot be clearly separated, likely because the multicrystalline Pt electrode was measured in ambient atmosphere.

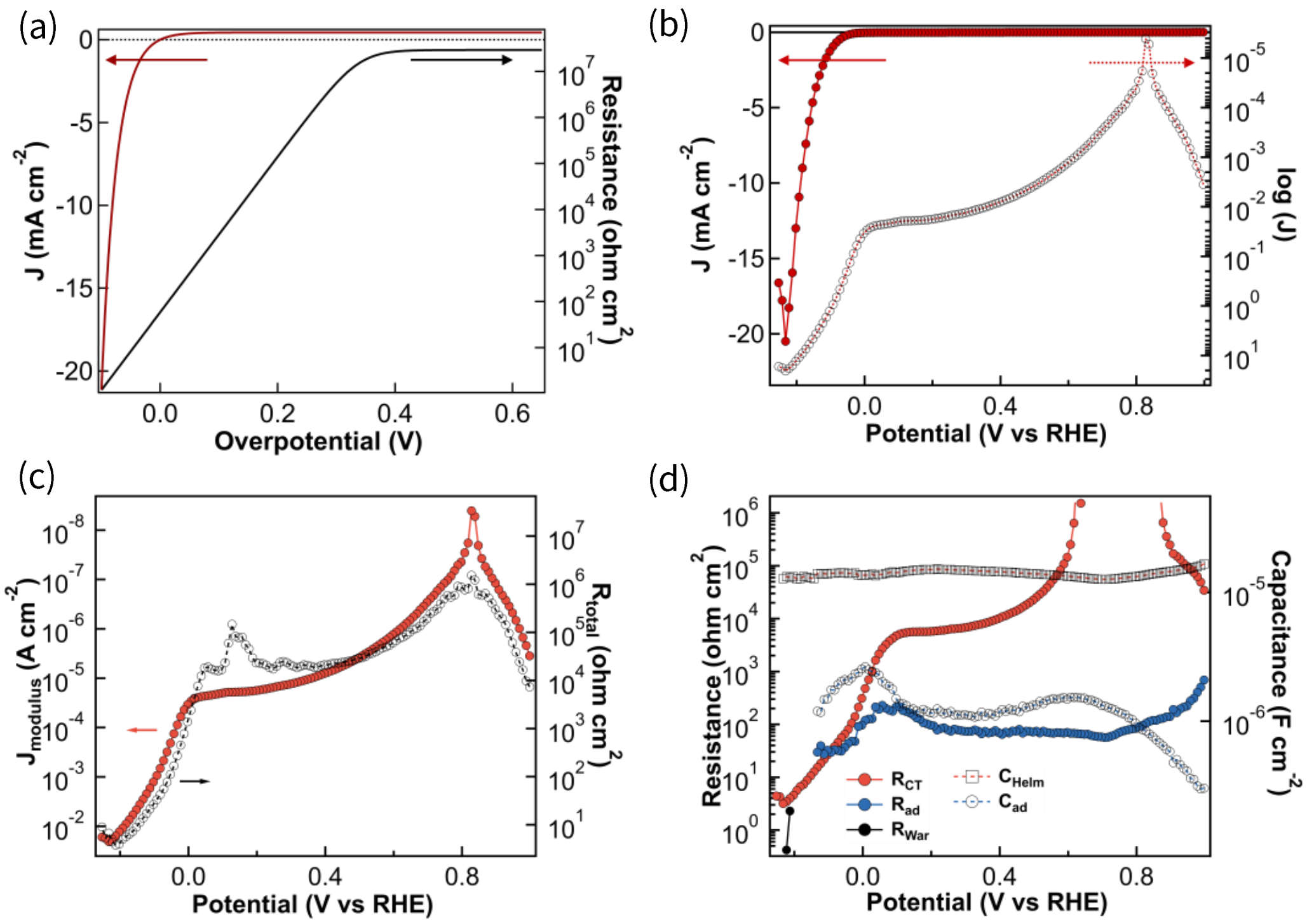

Figure S7. (a) Butler–Volmer equation simulation and associated total resistance of the simulated current (see text for more details). All the data presented in b, c and d is based on the same data presented in Fig. 3 of the main manuscript. (b) DC current density from the lowest frequency of the EIS measurement, plotted both linearly and logarithmically. (c) Log DC current density and associated total resistance from the derivative of bias potential and current density. (d) Resistances and capacitances from the EIS fitting with the Maxwell circuit in the potential region 1 V to -100 mV vs RHE. From -100 mV to -200 mV vs RHE with a reduced Randles circuit and afterwards with the Randles circuit.

Similar to the *J*–*V* curve, it is convenient before starting the actual fitting procedure of the EIS data to take a qualitative look (see Supplementary Note 10). From the Nyquist and, in particular, the Bode plots, it becomes evident that a simple equivalent circuit consisting of a series resistance R_S combined with a $(RC)_p$ is insufficient to describe the system over the entire potential range (see black dotted line representing an R_S-$(RC)_p$ fit in Fig. S8). Instead, two time constants can be identified.

To account for this behavior, the Maxwell equivalent circuit is commonly employed, and in some cases an additional Warburg element is included to represent diffusion processes.[29–31] Physically, the Maxwell

circuit describes charge transfer into the electrolyte while simultaneously accounting for adsorption of ionic species (e.g., protons or hydroxide ions) at the metal surface. Although this equivalent circuit is applied here without an in-depth discussion, it should be noted that, particularly in the anodic potential region, some authors favor the Voigt equivalent circuit or transmission line models, arguing that the growing platinum oxide layer can behave as a resistive or blocking overlayer.[27,32,33]

The existence of multiple proposed equivalent circuit models highlights the difficulty in selecting an appropriate representation. We remind here again that, unlike the Voigt or Matryoshka circuits, resistance values obtained from the Maxwell circuit cannot be directly read from the Nyquist plot.

Another important point that is often not clearly communicated is that the applied equivalent circuit can change multiple times depending on variations in the (photo)physical and electrochemical processes occurring in the device depending on the different potential regions (e.g. see Supplementary Note 8 on the different EC models used for the platinum electrode EIS data). In the present case, the Warburg diffusion element was rarely observable, and no distinct double-layer region could be identified under the applied measurement conditions. Furthermore, in the region of high cathodic faradaic currents, no resistance associated with hydrogen underpotential deposition was detected, effectively reducing the Maxwell equivalent circuit to a Randles-type circuit.

The complete fitting results are presented in Fig. S7d. R_{CT} closely follows the shape of the current–potential curve, as it represents the dominant resistance in this potential region. The observed reduction in current at highly negative potentials, accompanied by an increase in R_{CT}, can be attributed to gas bubble formation at the platinum electrode, which limits the effective electrode area accessible to the electrolyte solution.

Most important for the present study is the observation that upon passing 0 V vs RHE, a steady exponential decrease in R_{CT} accompanies the increase in cathodic faradaic current, and the equivalent circuit transitions from a Maxwell-type circuit to a Randles-type circuit.

Supplementary Note 10: Nyquist plots of the measured Pt electrolyte solution interface

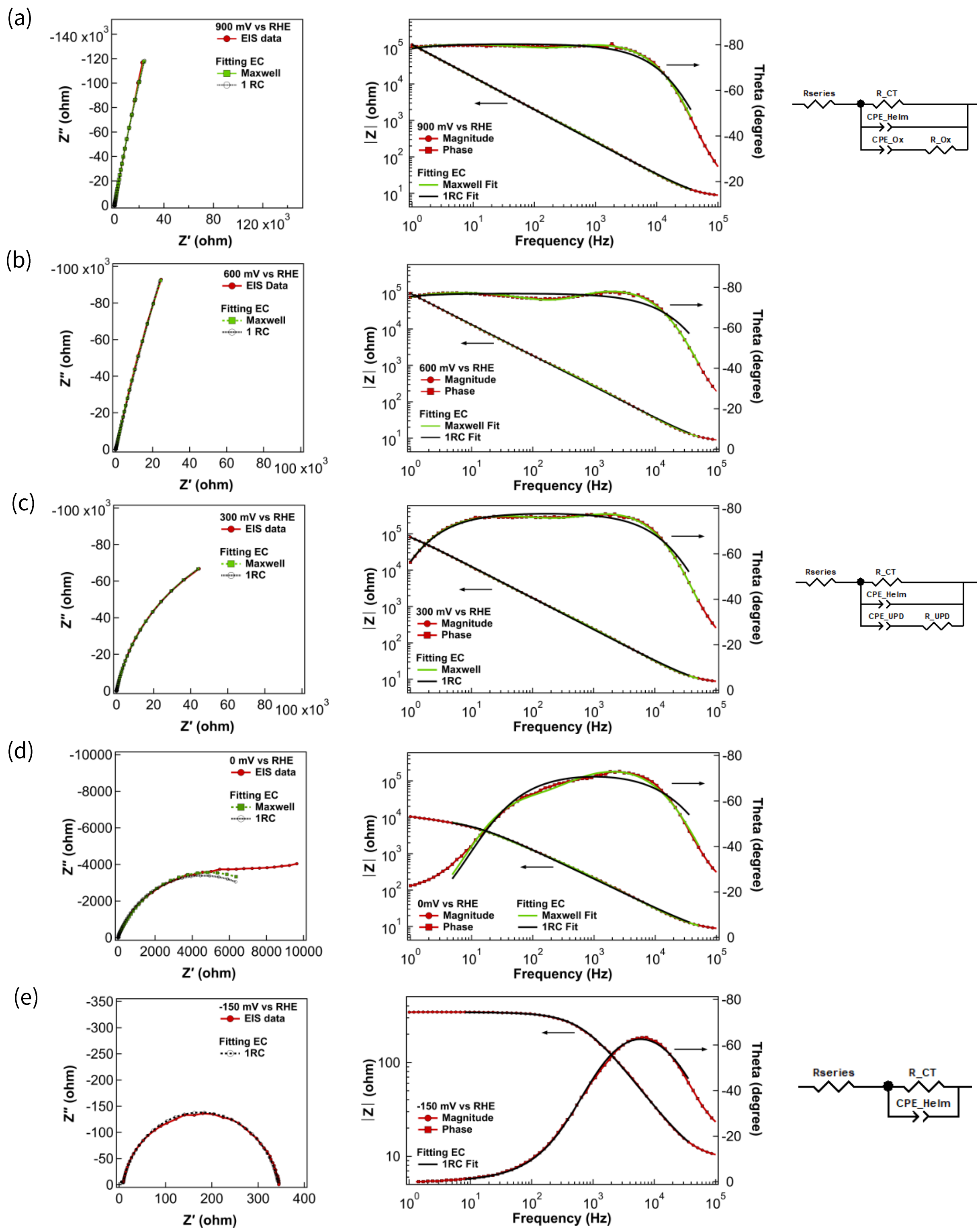

Figure S8. (a) – (e): Nyquist and Bode plots at different applied potentials of a multicrystalline Pt electrode in 1 M $HClO_4$. The right side shows the EC models used for the fitting. For the potential regions with more than one time constant the fit with a single $(RC)_p$ element is also shown.

Supplementary Note 11: Contact formation at a p–n diode

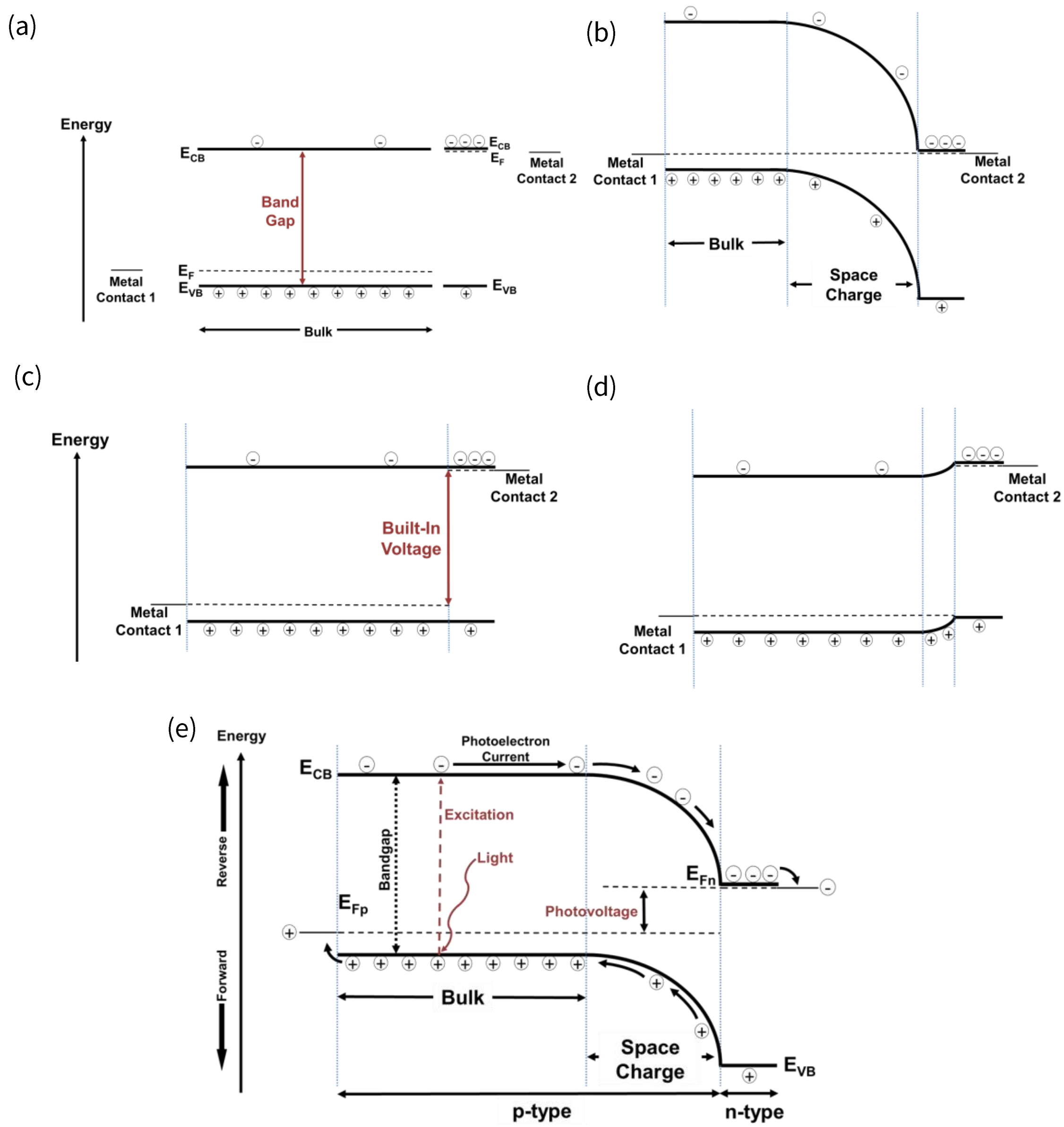

Figure S9. Silicon p–n$^+$ junction: (a) Before contact. (b) After contact: depletion condition at 0 V. (Polarizing the metal contact 1 to more negative potentials will lead to reverse bias conditions. Applying more positive potentials will lead to forward bias condition, where the solar cell is operated). (c) Flat band potential (ideally maximum photovoltage the junction can generate). (d) Accumulation condition. (e) In depletion and illumination conditions.

A p–n$^+$ diode is schematically shown in Fig. S9, where n$^+$ denotes a highly doped semiconductor with a high density of free charge carriers. Upon forming contact, charge carriers are exchanged between the p-type and n$^+$-type regions until thermodynamic equilibrium is reached and the Fermi levels align. As a

consequence, band bending forms at the p–n^+ interface, predominantly within the lower-doped p-type semiconductor. This process is analogous to a semiconductor–electrolyte interface, where the Fermi level of the semiconductor aligns with the redox potential of the electrolyte.

The resulting band bending gives rise to a space-charge region and its associated space-charge capacitance (C_{SC}), separating the depleted interfacial region from the field-free bulk semiconductor. At 0 V applied between the front and back contacts, the p-type semiconductor is in depletion, meaning that the space-charge region is depleted of majority carriers (holes), while the n^+ region remains largely unaffected (Fig. S9a,b).

Applying a more negative potential to the p-type semiconductor (higher electron energy) increases the band bending and drives minority carriers (electrons) toward the junction. Under dark conditions, the limited availability of electrons results in only a small cathodic leakage current, defining the reverse-bias regime (not shown here but see also Fig. 4d in the main manuscript). In contrast, applying a positive potential reduces the band bending and narrows the space-charge region, thereby lowering the internal electric field. This increases the probability for majority carriers (holes) to cross the junction, leading to an exponential increase in current as described by the diode equation (forward bias).

At a sufficiently positive potential, the band bending in the p-type semiconductor vanishes, defining the flat band potential E_{FB} (Fig. S9c). The built-in potential of the junction corresponds to the energy difference between the Fermi levels of the isolated p- and n^+-type materials prior to contact and represents the maximum achievable photovoltage under ideal conditions. Beyond the flat band potential, an accumulation layer forms in the p-type semiconductor, further increasing the dark current (Fig. S9d).

Although the equivalent circuit is formally similar to that used for a metal–electrolyte interface, the physical meaning of the elements and the underlying (photo)physical processes are fundamentally different. In a solar cell, the goal is to suppress dark currents, which corresponds to a high resistance representing the diode and shunt contributions. In contrast, at a metal–electrolyte interface a low resistance is desired, as it reflects efficient charge transfer from the electrode into the electrolyte and thus high catalytic activity.

Under illumination, photogenerated electron–hole pairs are created in the p-type absorber. The internal electric field in the depletion region efficiently separates these carriers, driving electrons toward the n^+ region and subsequently to the metal contact (Fig. S9e). This process is most efficient near 0 V and under reverse bias, where the dark current is minimal and the band bending assists charge separation. At open-circuit conditions, the photogenerated current is exactly compensated by the diode dark current.

Supplementary Note 12: *J–V* curve of a solar cell, diode EC model and EIS results

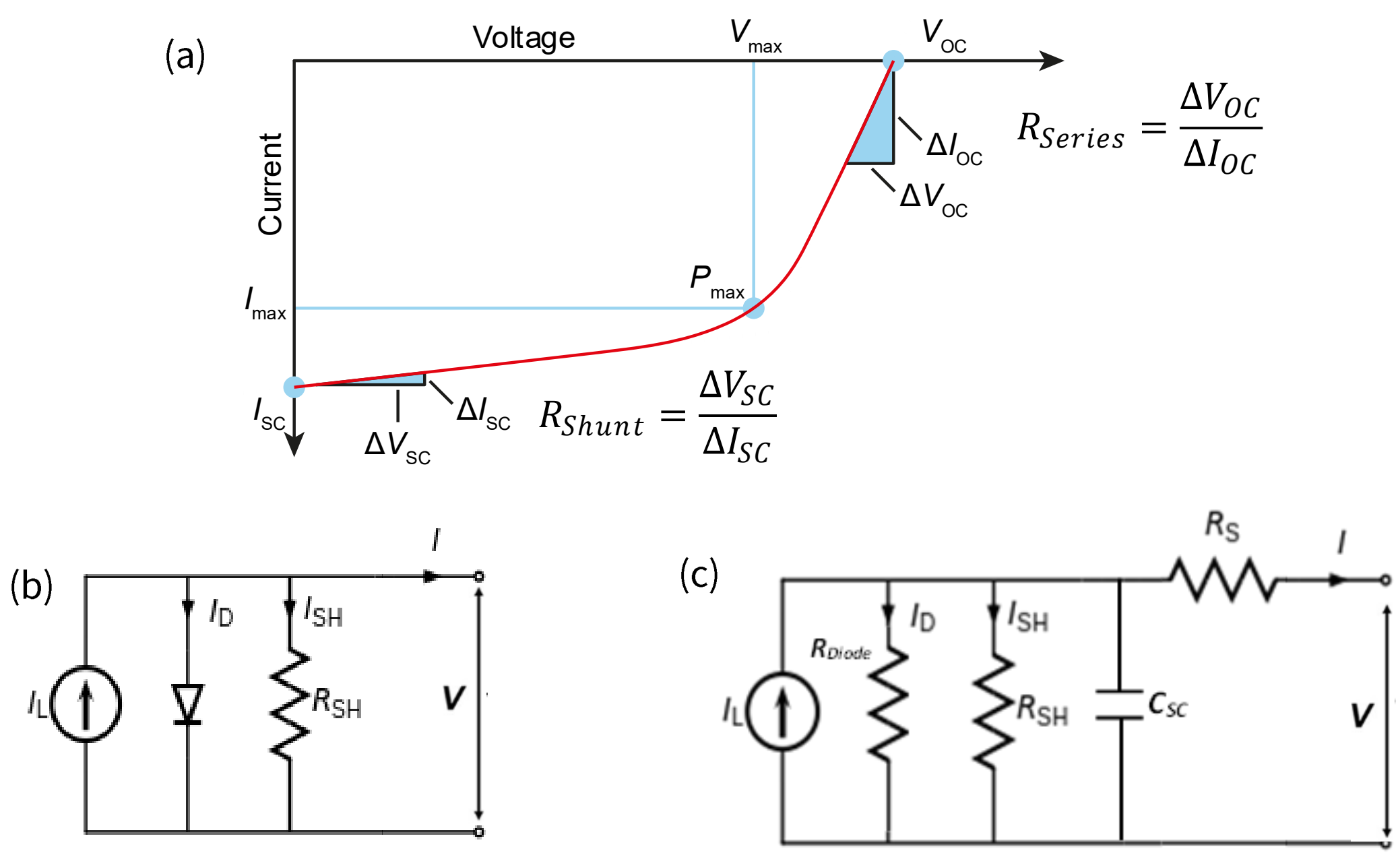

Figure S10. (a) Generic *J–V* curve of a solar cell with the key information. (b) Diode equivalent circuit. (c) Diode EC separated into single C and R elements and the series resistance. (d) *J–V* curve of a silicon solar cell.

The *J–V* curve of a solar cell under illumination is characterized by key parameters at a certain light intensity: the short circuit photocurrent (J_{sc}) at 0 V, the photovoltage (open circuit photovoltage V_{OC}), and the fill factor (FF) (Fig. S10a). The FF is the ratio of the maximum obtainable power (J_{max}·V_{max}) to J_{sc}·V_{OC}, and shows where the solar cell should be operated for maximum power output.[34]

Important information on the current–voltage characteristics can be extracted from the differential quotient of voltage with respect to current. In particular, the shunt resistance can be determined near short-circuit conditions, while the series resistance can be determined near open-circuit conditions (Fig. S10a):

$$R_{Shunt} = \frac{\Delta V_{SC}}{\Delta J_{SC}}$$

$$R_{Series} = \frac{\Delta V_{OC}}{\Delta J_{OC}}$$

By taking the derivative of the applied voltage with respect to the current, the potential-dependent total resistance, R_{Total}, is obtained (see also Fig. 5d, Fig. 6d and Fig. S11c). The R_{Total} value should correspond to the overall resistance (the sum of all resistances) determined from the EIS fitting.

The model is normally extended with an additional channel for parasitic current loss, which is called a shunt. A shunt current is an additional leakage path in the diode, increasing the dark current in forward bias and reducing the efficiency of a solar cell (see also Supplementary Note 13). To implement the parasitic shunt current, an additional resistance, the shunt resistance (R_{Sh}), is included in parallel to the diode element (Fig. S10b). Ideally, the shunt resistance should be as high as possible to reduce current loss. It is important to note that distinguishing between R_{Diode} and R_{Sh} is not straightforward as we will only measure the sum of all parasitic currents and the dark current. Therefore, there is only one resistance for these processes detectable. For simplicity, we will refer only to R_{Diode} but it is important to note that it includes R_{Sh}.

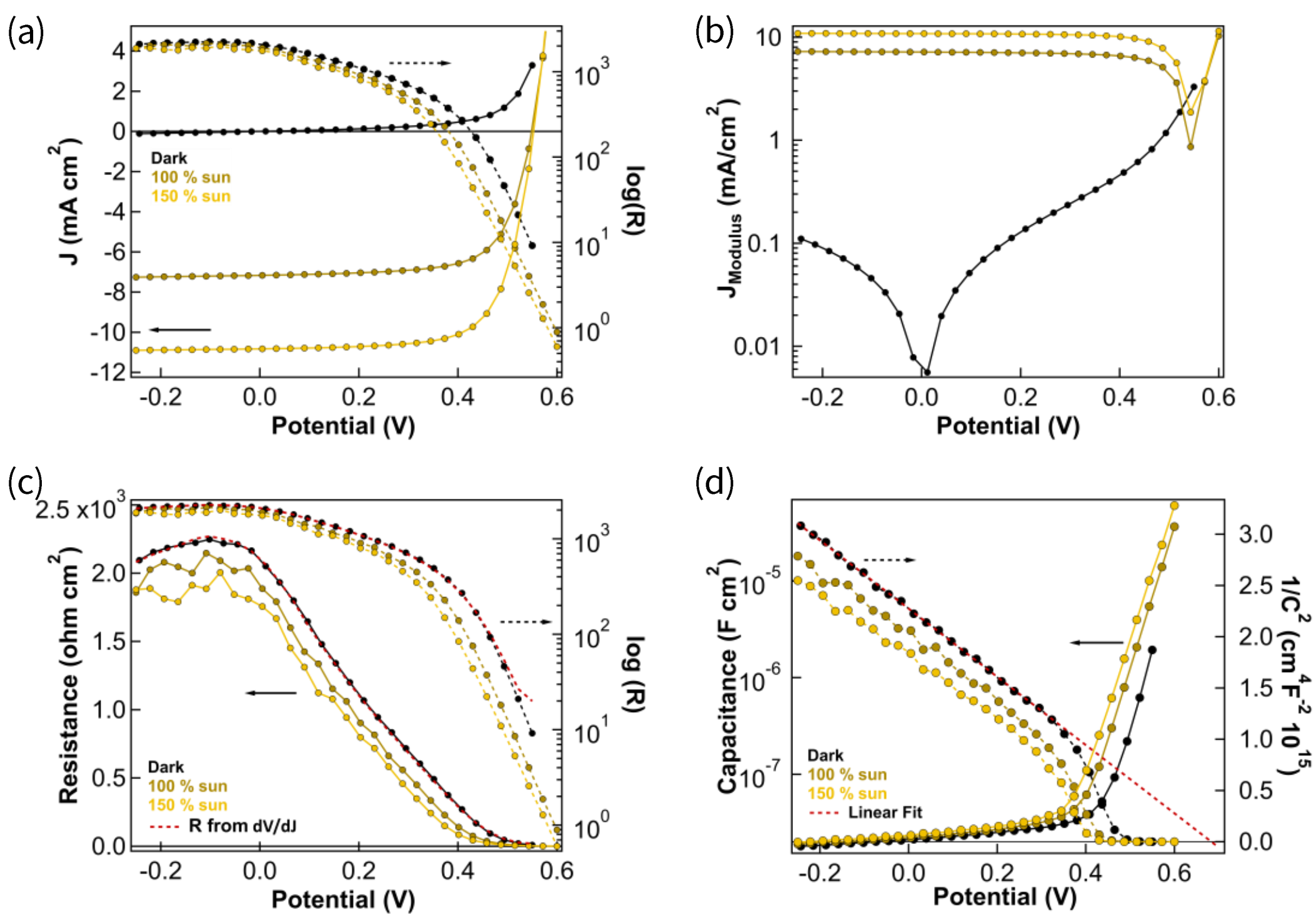

Figure S11. Same data as Fig. 5 in the main manuscript but with additional data at 150 % light intensity. (a) *J–V* curve of a silicon solar cell. (b) *J–V* curve plotted in log scale. (c) R_{Diode} and R_{Total} (red dotted line represents the DC resistance determined from the derivative of voltage vs the current dV/dJ) plotted in linear and in log scale. (d) Space-charge capacitance and Mott–Schottky plot.

The extracted current from the EIS measurement below 0 V is cathodic and changes sign when reaching positive voltage. It is the transition from reverse to forward bias as described in Fig. S11a and b. The current starts to increase exponentially when entering the forward regime as described by the diode equation while the diode resistance will inversely decrease exponentially. As in the case of the electrochemical interface, the derivative of the applied voltage vs the current passed yields the total

resistance of the device which matches the resistance from the fitting procedure showing the validity of the fitting.

One can clearly observe that the resistance values for measurements under illumination are only slightly reduced (Fig. S11c). The reduction in resistance is based on an increased shunt current which is reflected in a lower diode resistance (since it is parallel to R_{Diode} as explained above). The C_{SC} rises when transitioning from reverse to forward voltage region as the space-charge region is narrowing due to the reduced band bending at the p–n junction interface (see also Fig. S11d).

Continuing to apply more forward bias, the V_{OC} of the solar cell is passed (as mentioned before the photocurrent is now equal to the dark current of the diode) followed by the flat band potential. For a well-performing solar cell, the V_{OC} at 1 sun approaches the actual flat band potential.

The space-charge capacitance of the semiconductor (in depletion) is widely used to determine the flat band potential and the doping density via a Mott–Schottky (MS) plot analysis, as depicted in Fig. S11d (see also Supplementary Note 15 on Mott–Schottky plots). Both are important characteristics of the light absorbing semiconductor.

In strong depletion (in reverse bias), we observe the expected linear behavior in the MS plot. However, when the potential approaches E_{FB}, a deviation from linearity due to the chemical (or diffusion) capacitance is observed. The increased concentration of majority carriers at this interface results in additional stored charge giving rise to the chemical capacitance. The chemical capacitance exists in parallel to C_{SC}. Consequently, in this potential range, the measured capacitance corresponds to the sum of both capacitances. The presented solar cell device shows the influence of the chemical capacitance from about 400 mV onward, as observed by the deviation of the MS plot from the linear fit (Fig. S11d).

Supplementary Note 13: Extended Diode Equation

The diode equation presented above and in the main manuscript is a simplification. In a more extended diode model, both a series resistance and a shunt resistance are included. The series resistance accounts for resistive losses arising from cables, contacts, and bulk transport, while the shunt resistance represents parasitic leakage currents through alternative current pathways. When both resistances are included, the current–voltage relationship can be expressed as:[34]

$$J = J_{Diode} - J_{Photo} = J_o\left(e^{\frac{q(V-JR_S)}{nkT}} - 1\right) + \frac{V-JR_S}{R_{shunt}} - J_{Photo} \qquad \text{(Eq. S2)}$$

Supplementary Note 14: Nyquist and Bode plots of the silicon solar cell

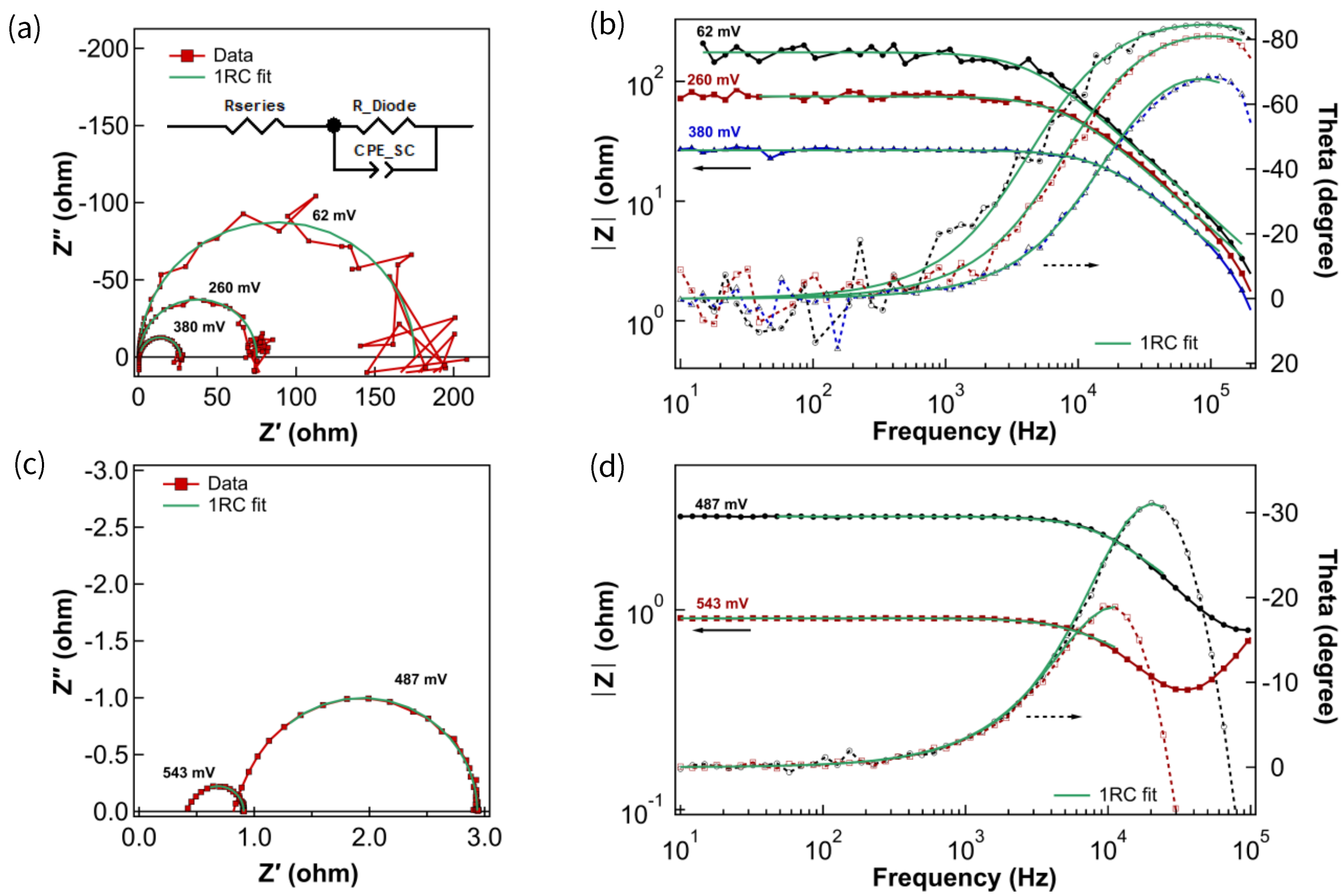

Figure S12. Nyquist and Bode plots of the EIS measurement and the fitting results of the silicon solar cell. (a) Nyquist plots at 62, 260 and 380 mV and fitting results. The inset shows the EC used to model the data. (b) Bode plots and fitting results for the same data as in (a). (c) Nyquist plots at 487 and 543 mV. (d) Bode plots and fitting results for the data in (c).

Supplementary Note 15: Mott–Schottky plots

Mott–Schottky plots are widely used for the characterization of semiconductors and semiconductor junctions, such as solar cells or semiconductor–electrolyte interfaces. The method is based on the determination of the space-charge capacitance of the (lower doped) semiconductor. Importantly, the Mott–Schottky relation is only valid for the C_{SC} in the depletion regime of the semiconductor.

Especially for MS plots there are several good literature sources: Hankin *et al.,* Sivula *et al.* and Ravishankar *et al.* to name only a few.[17,35,36] These works provide valuable guidance for inexperienced users and help clarify common questions and pitfalls associated with MS analysis. Here, we only emphasize selected aspects based on difficulties frequently encountered in practice.

Mott–Schottky analysis is a technique used in solid-state physics and electrochemistry to determine the flat band potential and the doping density of semiconductor materials. By measuring the space-charge capacitance of a p–n junction or a semiconductor–electrolyte interface as a function of applied potential, a Mott–Schottky plot ($1/C^2$ versus applied potential) can be constructed, as shown in Fig. S11d. This analysis is only meaningful when the measured capacitance corresponds to the space-charge capacitance of the semiconductor in depletion.

Plotting $1/C^2$ as a function of applied potential should yield, at least over a certain potential range, a linear relationship. The slope of the MS plot is inversely proportional to the doping density: a steeper slope corresponds to a lower doping density, and vice versa. The extrapolation of the linear region to the voltage axis yields the flat band potential (offset by kT/q), which corresponds to the potential at which band bending in the semiconductor vanishes. This analysis is crucial for understanding the electronic properties of semiconductors in a wide range of applications.

The potential dependent space-charge capacitance of a p-type semiconductor in depletion is defined as:[34]

$$C_{SC} = A\left(\frac{qN_A\varepsilon\varepsilon_0}{2}\right)^{1/2}\left(V_{FB} - V - \frac{kT}{q}\right)^{-1/2} \qquad \text{(Eq. S3)}$$

which can be rearranged to:

$$\frac{1}{C_{SC}^2} = \frac{-2}{A^2N_A\varepsilon\varepsilon_0 q}\left(V - V_{FB} + \frac{kT}{q}\right) \qquad \text{(Eq. S4)}$$

where ε is the dielectric constant of the electrode, ε_0 is the vacuum permittivity, q is the charge of the electron, N_A is the acceptor density, A is the (surface) area, V the applied potential, V_{FB} the flat band potential, k is the Boltzmann’s constant and T is the temperature.

It is important to note that in a buried junction, the capacitance of the lower doped semiconductor is measured, since capacitors in series are dominated by the smallest capacitance (see section on Kirchhoff’s laws). In the case of a semiconductor–electrolyte interface, the space-charge capacitance of the semiconductor is usually observed, as it is typically at least an order of magnitude smaller than the Helmholtz (double-layer) capacitance. If this condition is not fulfilled, the Helmholtz capacitance must be explicitly taken into account.[17]

Finally, we comment on a commonly used but potentially problematic approach to MS analysis. Often, only one or two frequencies are used to determine the capacitance at each applied bias potential, and these values are then used to construct an MS plot. In this approach, the user attempts to extract the capacitance of a specific process from a highly reduced EIS measurement. Ideally, the selected frequency should correspond to the characteristic frequency of the relevant RC element, typically near the maximum of the semicircle in the Nyquist plot, where the capacitance can be directly related to the space-charge capacitance.

However, several issues may arise. First, even in this reduced approach, sufficient stabilization time is required to ensure a stable DC current during the AC measurement. Second, if the chosen frequency is not close to the characteristic time constant of the space-charge capacitance, it becomes unclear which capacitance is being probed, and whether additional capacitive contributions are present. Third, frequency dispersion must be considered: upon changing the applied potential, the characteristic time constant—and thus the characteristic frequency—of a process may shift.

Figure S13 illustrates this effect, where a change in resistance (from the red to the green semicircle) shifts the characteristic frequency to higher values while the capacitance remains constant. If a single fixed frequency is used over the entire potential range, such shifts can lead to systematic deviations from the true $1/C_{SC}^2$ behavior. Consequently, inaccurate values for the flat band potential and doping density may be obtained.

Performing full EIS measurements over a wide frequency range at each bias potential and fitting each spectrum provides a much more reliable basis for constructing Mott–Schottky plots. Once this comprehensive analysis has been completed, reduced-frequency MS measurements can be used as a cross-check to identify suitable frequencies and to assess the extent of frequency dispersion in the system.

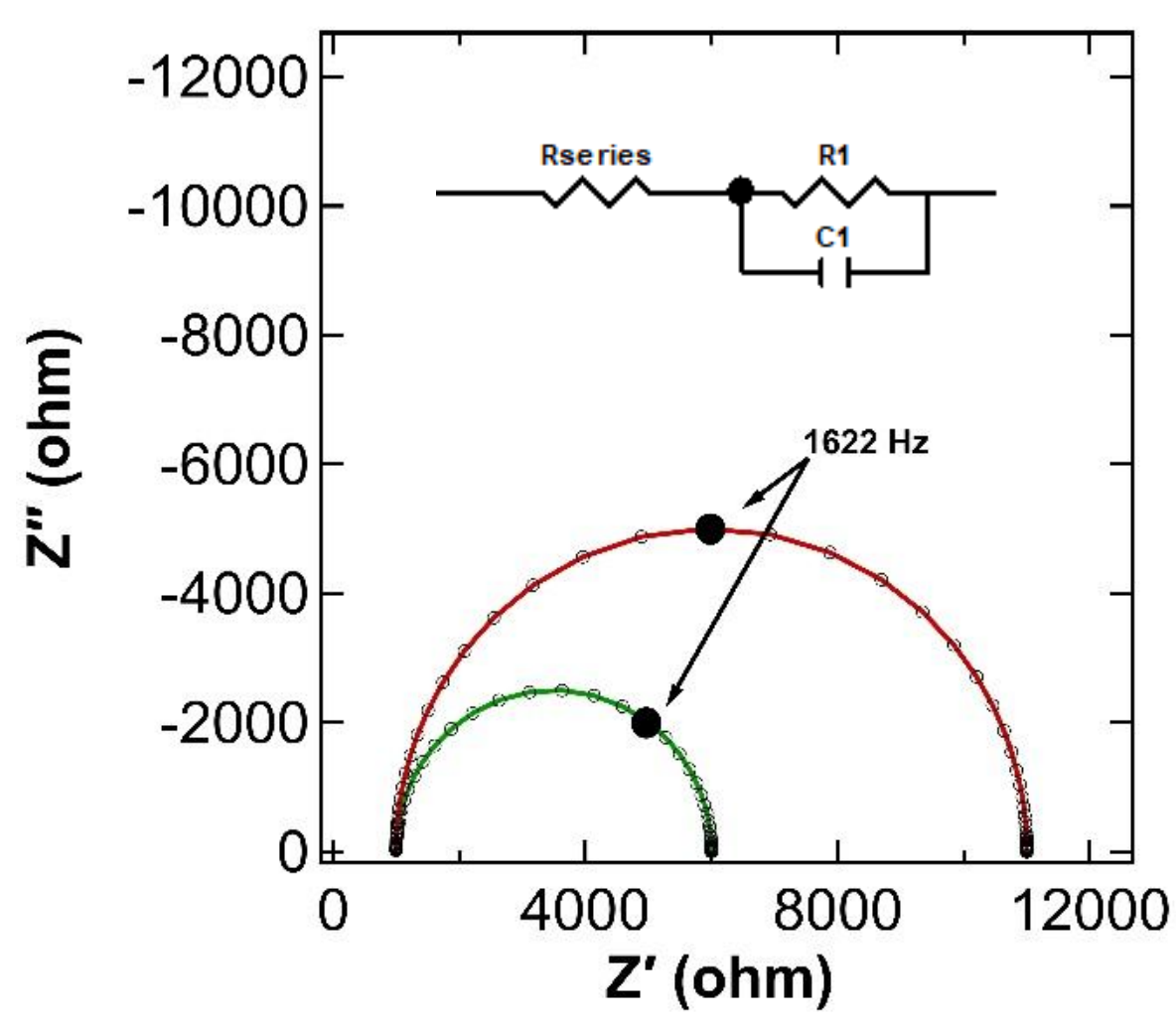

Figure S13. Nyquist plots showing frequency position shift with parallel resistance change. Red: R_{series}: $1{\cdot}10^4$ ohm; C1: $1{\cdot}10^{-8}$ F; R1: $1{\cdot}10^4$ ohm. Green: R1: $5{\cdot}10^3$ ohm; R_{series} and C1 are the same.

Supplementary Note 16: Contact Formation in a Buried Junction PEC device

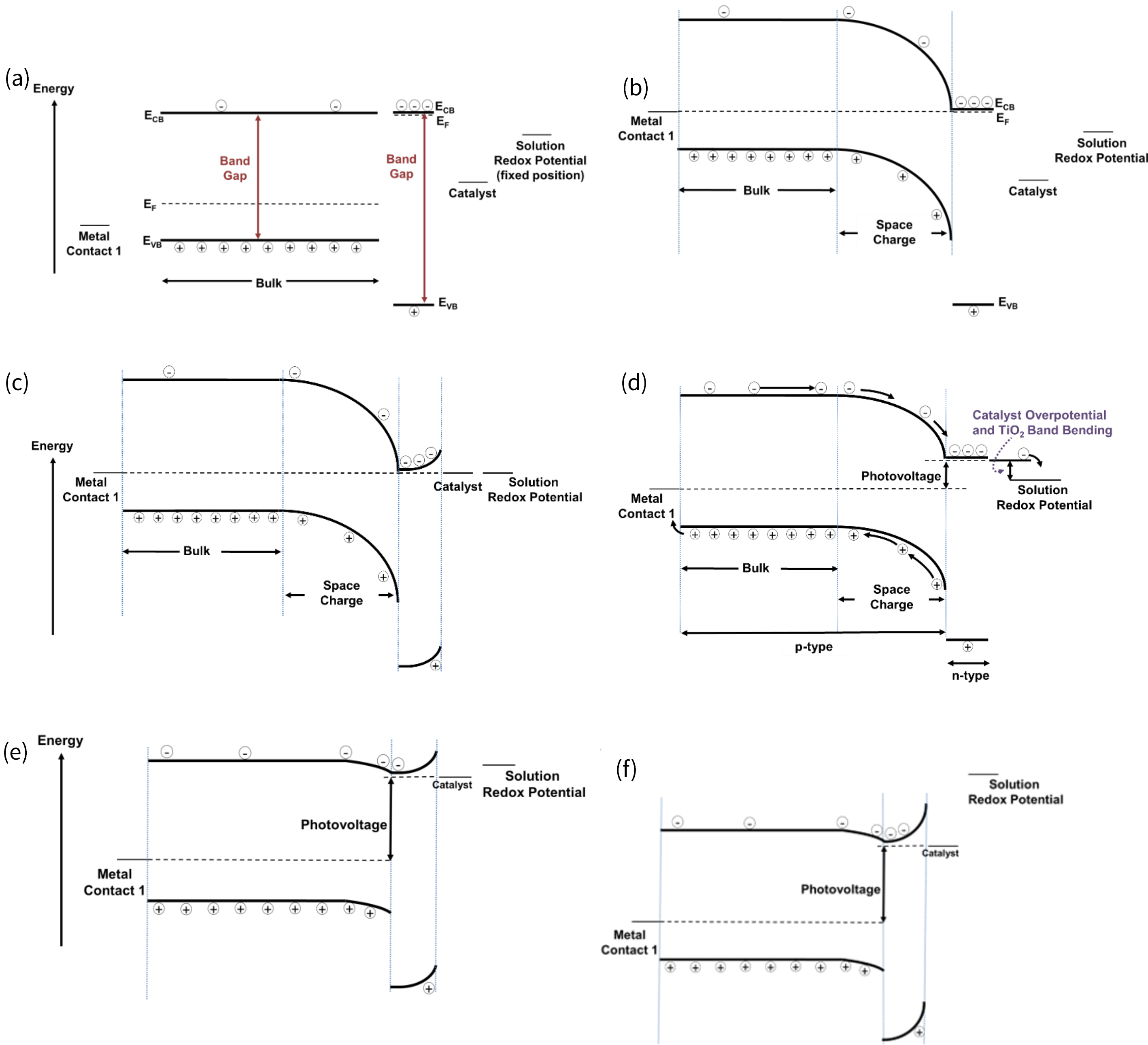

Figure S14. Band diagrams for a p-type semiconductor/n-TiO_2/electrolyte solution PEC system: (a) Before contact. (b) Only the PV part is connected. (c) Complete system aligned to solution redox potential. (d) In depletion under illumination with the catalyst overpotential and TiO_2 band bending overcome. (e) System under illumination before onset potential of photocurrent. The Fermi level of TiO_2 is not pinned to the electrolyte anymore due to the applied potential. (f) Under strongly biased conditions, the TiO_2 band edge may also become unpinned and the whole p–n junction shifts with increasing positive potential.

The band diagram of the PEC cell is shown in Fig. S14. In a first step, the contact between p-type Si and n-type TiO_2 is formed, analogous to the p–n junction in a conventional solar cell (Fig. S14b). Since TiO_2 is more highly doped than the p-type Si, the majority of the potential drop develops within the silicon. Consequently, band bending occurs primarily in the p-type Si, while the TiO_2 remains approximately field-free.

In a second step, the TiO_2 layer is brought into contact with the electrolyte solution. Upon contact, the Fermi level of the TiO_2 adjusts to the redox potential of the solution. Importantly, this adjustment does not affect the band bending at the buried p-Si/TiO_2 junction, which remains unchanged after contact with the electrolyte (Fig. S14c).

Figure S14d illustrates the equilibrium condition after contact formation (without kinetic limitation for the exchange of charge over the solid liquid interface) under illumination.

Figures S14e and S14f illustrate the positive potential region prior to the onset of photocurrent, where an unpinning of the semiconductor band edges partly occurs. A detailed discussion of this behavior is provided in Supplementary Note 19 (Dual Working Electrode Measurements).

Supplementary Note 17: The measured EIS of a p-Si/n-TiO_2/Pt in 1 M H_2SO_4 at 10% sun illumination by white-light LED

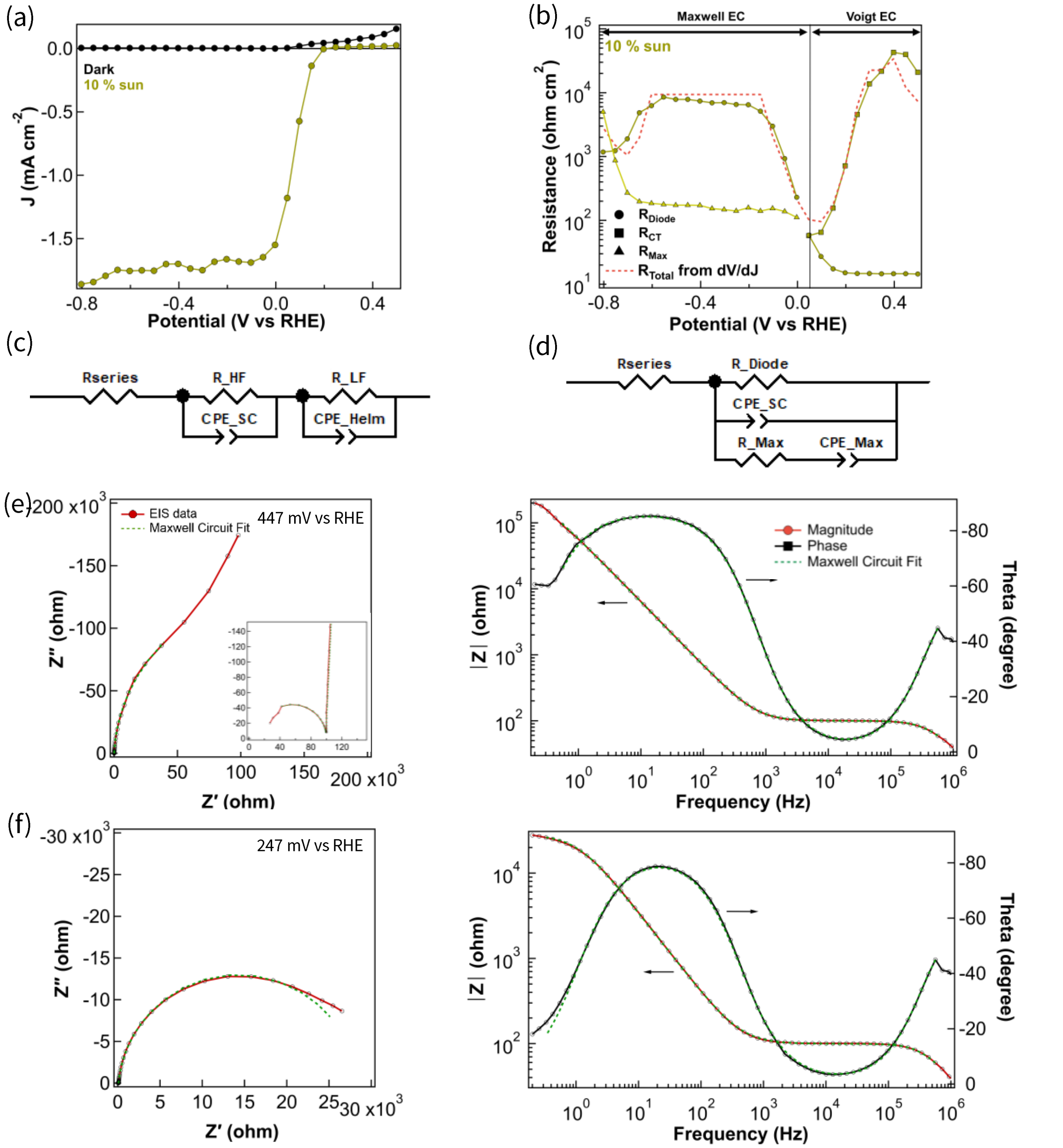

Figure S15. (a) Steady state *J*–*V* response of a p-Si/n-TiO_2/Pt photocathode under 10% sun illumination. (b) Extracted resistances and the total resistance determined from the dV/dJ derivative. (c) EC model used for the fitting before photocurrent onset. (d) EC model for the fitting after photocurrent onset. (e) and (f) Nyquist and Bode plots for different bias potentials (red: experimental data; green dotted: EIS fit).

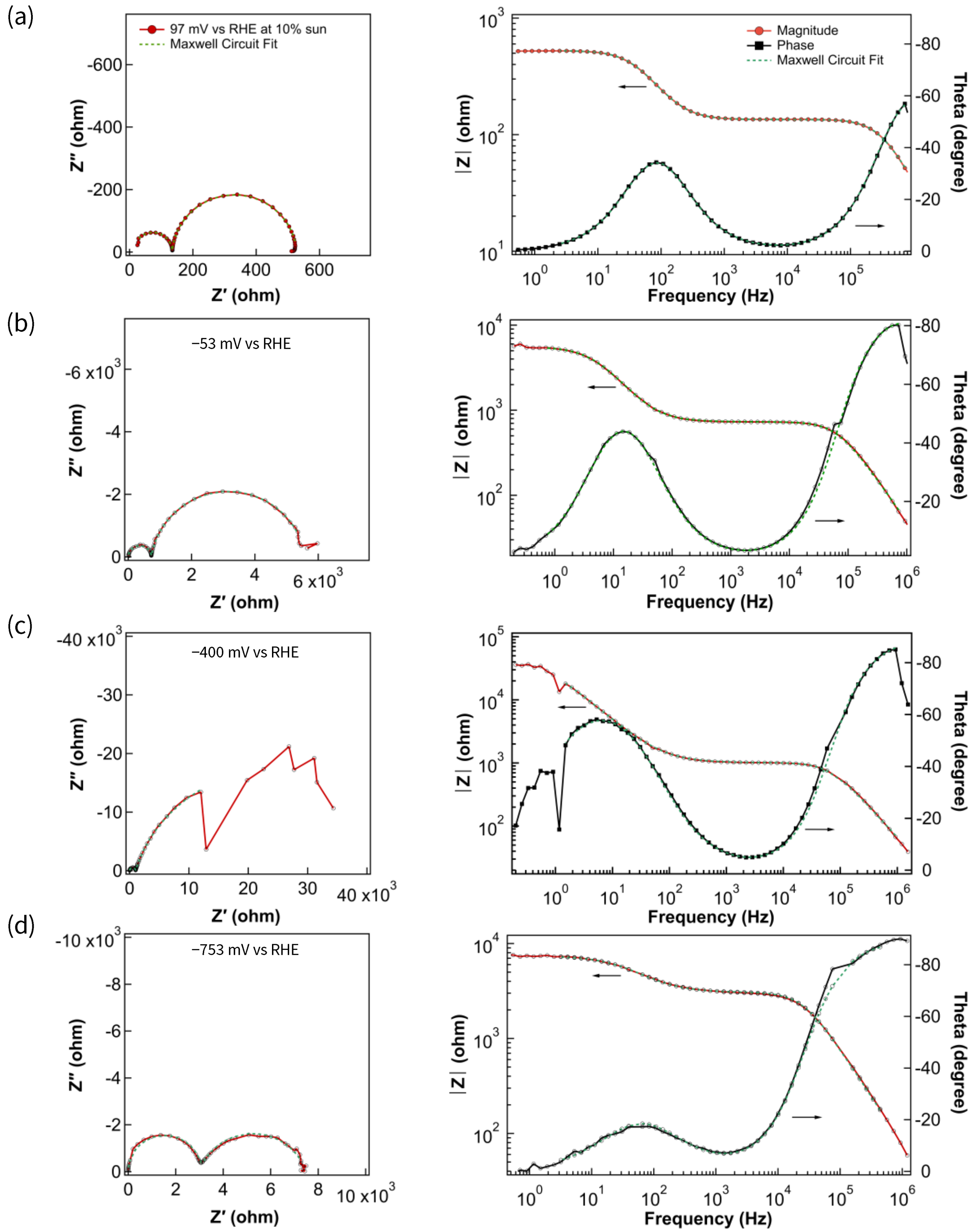

Figure S16. (a), (b), (c) and (d) Nyquist and Bode plots for different bias potentials. Continued from Fig. S15.

Supplementary Note 18: Three different light intensities of a p-Si/n-TiO_2/Pt

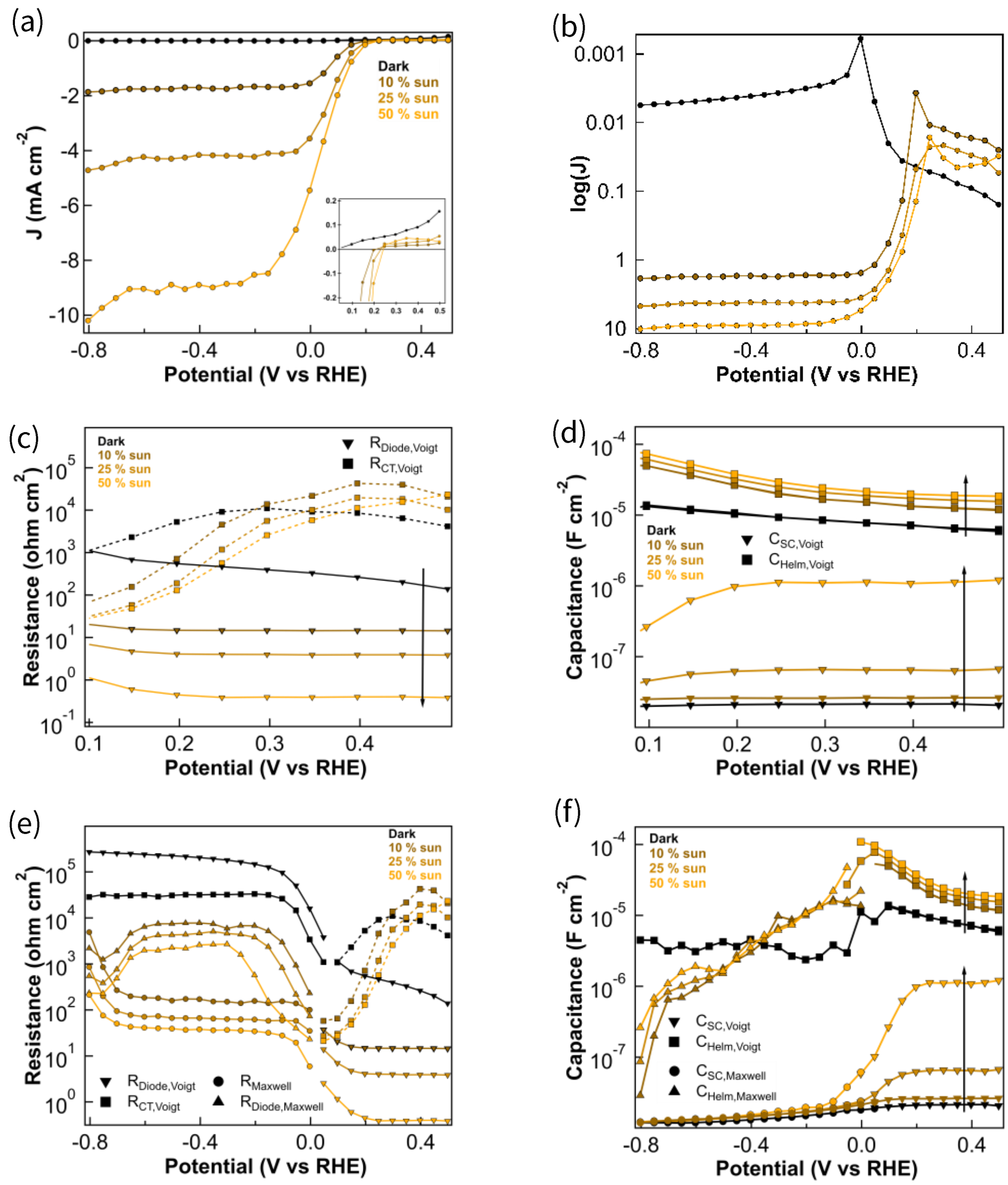

Figure S17. (a) DC current from the lowest frequency (0.2 Hz); inset: anodic current before photocurrent onset. The 10% sun measurement is the same as presented in the main text. (b) Modulus of the DC current plotted in log scale. (c,d) Resistances and capacitances before photocurrent onset. (e,f) Resistances and capacitances after onset. Arrows indicate the trend with increasing light intensity.

Supplementary Note 19: Implications of the C_{SC} before the photocurrent onset and Dual Working Electrode Measurement

Interestingly, C_{SC} is constant in the potential range positive of V_{OC} for a fixed light intensity. This behavior is unexpected when compared to a conventional solar cell, where an exponential increase of the space-charge (and/or diffusion) capacitance is typically observed under forward bias conditions.

The origin of this unexpected phenomenon is the unpinning of the surface potential of the solid-state device from the redox potential of the electrolyte solution. To fully understand this behavior, results from dual working electrode (DWE) measurements must be considered to refine the energetic picture of the device.[37] In DWE measurements, the back contact potential of the device is controlled, while a second working electrode measures the potential at the semiconductor–electrolyte interface relative to a reference electrode. This technique allows determination of the Fermi level splitting between the front and back contacts under illumination as a function of the applied back contact potential.

Let us now examine dual working electrode measurements, which provide further insight into the processes occurring at the different interfaces of the PEC device. DWE measurements employ an additional sensing electrode at the top surface of the PEC device to probe the potential at the semiconductor–electrolyte interface while independently controlling the back contact potential. This approach allows direct determination of the voltage difference between the back contact and the front contact (here, the TiO_2 layer).[37,38] One key outcome of this technique is the ability to identify the magnitude of the photovoltage generated by the buried p–n junction and the applied potential at which this photovoltage is established.

Cui *et al.* showed that prior to the onset of the photocurrent, the Fermi level of TiO_2 remains clearly positive of the hydrogen evolution potential (so does not have enough driving force for hydrogen production), while the photovoltage of the buried junction is already fully developed (Fig. S18a). This observation has several important implications. First, it indicates that TiO_2 forms a depletion layer at the TiO_2/electrolyte interface that is larger than initially expected. Second, it implies a shift of the absorber energetics, i.e., a downward shift of the vacuum level of the photoabsorber relative to the solution (Fig. S18b).

In this potential regime, two distinct features may be observed in the Nyquist plot: one corresponding to the charge-transfer resistance of TiO_2 toward the hydrogen evolution catalyst, and a second associated with the intrinsic charge-transfer resistance of the catalyst itself.[39,40]

These observations allow us to refine our initial assumption that the TiO_2 Fermi level is rigidly pinned to the redox potential of the electrolyte. Instead, the DWE results demonstrate that this pinning condition does not necessarily apply over the entire potential range.

Figure S18b illustrates the energetic situation inferred from the DWE measurements. The photoabsorber is already in depletion, while the TiO_2 Fermi level remains several hundred millivolts more positive than the hydrogen evolution potential. Upon decreasing the applied potential (i.e., moving to more negative potentials), the TiO_2 Fermi level progressively shifts toward the redox potential of the electrolyte. Importantly, the photovoltage between the absorber and the TiO_2 remains largely unchanged until the

TiO_2 Fermi level reaches the hydrogen evolution potential and the overpotential of the catalyst is overcome. In the potential region where TiO_2 is in depletion at the TiO_2/electrolyte interface, a Mott–Schottky analysis can be performed.

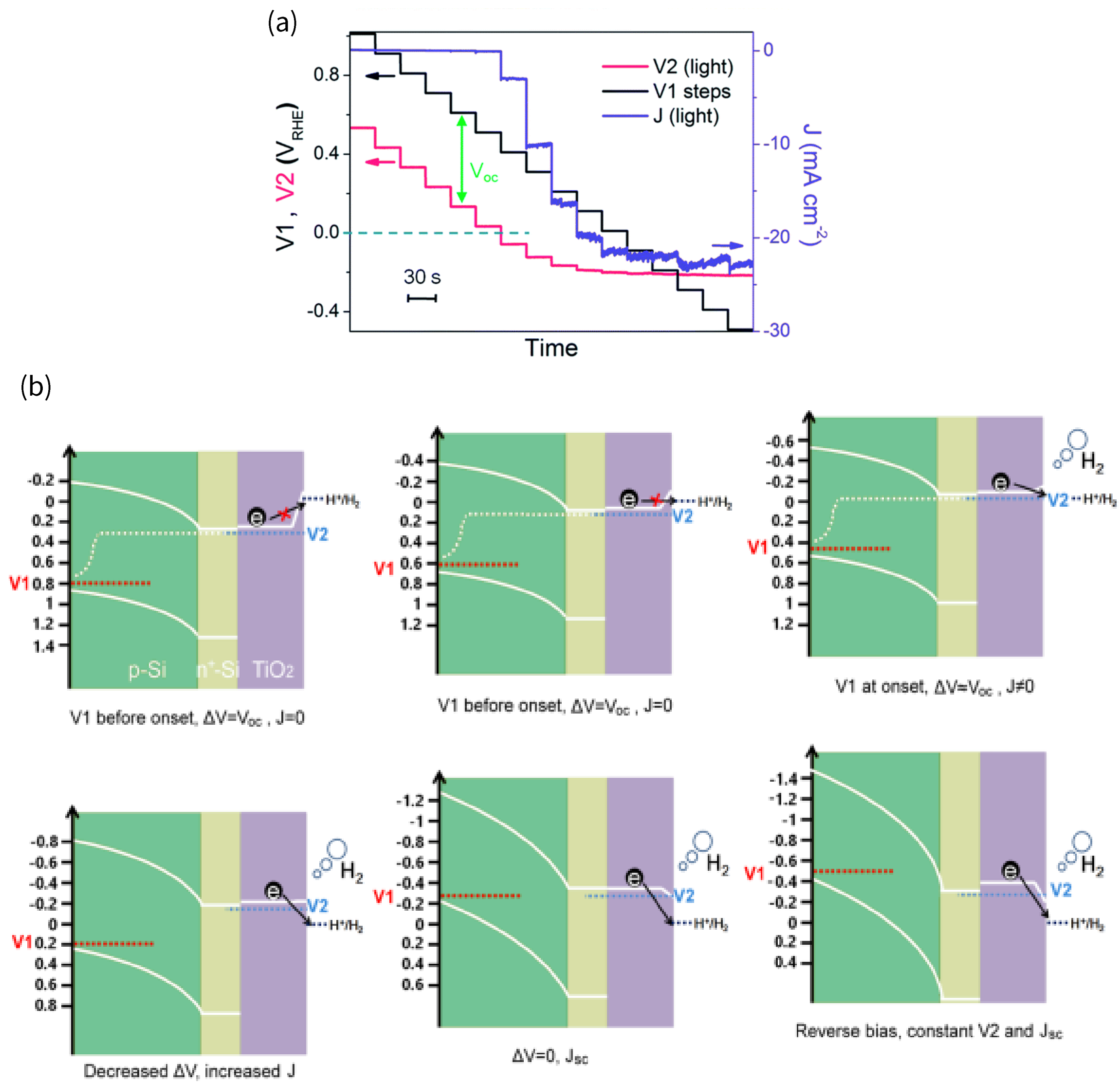

Figure S18. (a) Dual working electrode measurement of p–n$^+$ junction photocathode PEC device. (b): Showing the change in band bending at the different interfaces scanning from forward to reverse bias (see also description below). Reproduced from Ref. [37] under the terms of the Creative Commons CC BY 3.0 license (http://creativecommons.org/licenses/by/3.0/). Copyright [2018], Cui *et al.*

The implications of the DWE measurements for the investigated system are straightforward. They show that prior to the photocurrent onset, the band edges of the TiO_2 are not pinned to the electrolyte solution (Figs. S14e, S14f, and S18), while the buried p–n junction has already developed a Fermi level

splitting due to illumination. In this regime, the p-type semiconductor is already in depletion and a photovoltage is established.

The constant C_{SC} observed in this potential region indicates that the band bending at the buried p–n junction interface does not further change upon scanning the applied potential to more positive values. Instead, variation of the back contact potential results in a rigid shift of the entire energy diagram against the redox potential in solution. Consistent with this interpretation, no diffusion capacitance is observed in the EIS data, indicating that the p-type semiconductor does not enter the accumulation regime.

Supplementary Note 20: Mott–Schottky plots of the PEC electrode

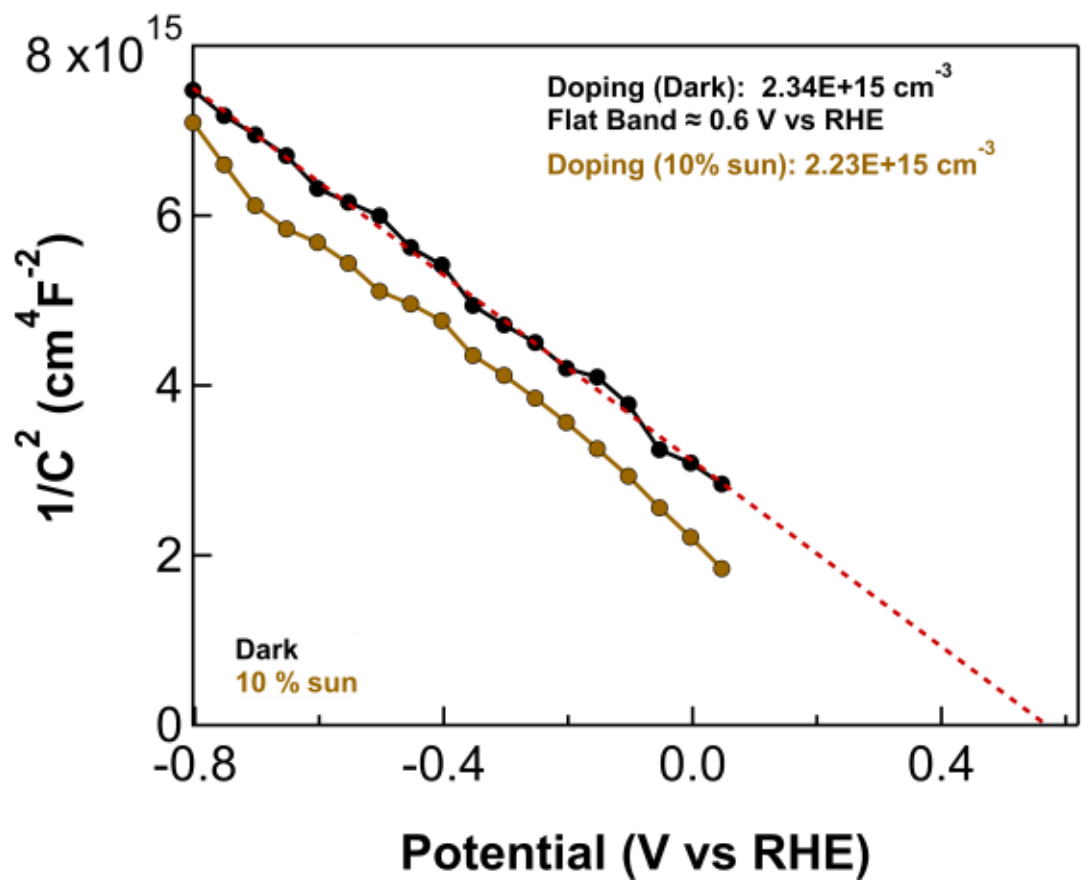

Figure S19. Using the C_{SC} for a Mott–Schottky plot and extract doping density and flat band potential for the silicon device.

Glossary

AC current (Alternating Current) is an electric current that periodically reverses direction, typically varying sinusoidally with time.

Accumulation is when majority charge carriers accumulate at the surface of a semiconductor due to an applied voltage, increasing their concentration in that region compared to the bulk of the material.

The **auxiliary (counter) electrode** completes the circuit by carrying the counter current for the reaction at the working electrode. This lets the working electrode be studied accurately because the reference electrode can measure the voltage without being affected by the current.

Bode plot is a graph that displays the frequency response of an electrochemical system by plotting the impedance magnitude and phase angle versus frequency, helping to identify resistive, capacitive, and inductive behaviors across frequencies.

Butler–Volmer equation describes the relationship between the current density and the overpotential at an electrode during an electrochemical reaction, combining both the forward (oxidation) and reverse (reduction) reaction rates to model the kinetics of electron transfer.

Capacitance is the ability of a system or component to store electric charge when a voltage is applied, typically measured as the amount of charge stored per unit voltage. The unit is farads.

Charge-transfer resistance is the resistance to electron flow across the electrode–electrolyte interface during a redox reaction, reflecting the kinetics of the electron transfer process.

Conduction band is the range of electron energy levels in a solid where electrons are free to move and conduct electricity, lying above the valence band and typically empty in insulators or partially filled in conductors and semiconductors.

CPE (Constant Phase Element) is an electrical circuit element used to model non-ideal capacitive behavior in electrochemical systems, where the phase angle of the impedance is constant but not exactly −90°, reflecting surface roughness or heterogeneous interfaces.

Cyclic voltammetry is an electrochemical technique where the voltage applied to the working electrode is swept linearly back and forth between two limits while measuring the resulting current, used to study redox reactions and analyze electrochemical properties of a system.

DC current (Direct Current) is the unidirectional flow of electric charge, meaning the current moves consistently in one direction.

Depletion (condition) refers to the removal of majority charge carriers from a region or interface by an electric field forming at the junction. It results in a zone depleted of majority charge carriers leaving behind the immobile dopant ions.

Diode resistance refers to the effective, bias-dependent resistance of a diode to current flow. It is not a fixed quantity but depends on the operating voltage of the diode. In forward bias, this resistance is

given by the dynamic (differential) resistance derived from the slope of the current–voltage characteristic, whereas in reverse bias the diode exhibits a very large resistance dominated by leakage or shunt pathways.

Doping is the intentional addition of small amounts of impurities to a semiconductor to modify its electrical properties by increasing the number of free charge carriers — electrons (n-type) or holes (p-type).

Electrochemical Impedance Spectroscopy (EIS) is a technique that measures the response of an electrochemical system to a small alternating voltage (AC) signal over a range of frequencies, providing detailed information about reaction kinetics, charge transfer, capacitance, diffusion, and other interfacial properties.

Electrolyte is a substance that produces mobile ions and thereby enables ionic conduction under an applied electric field. It may be a salt, acid, base, molten compound, or solid ionic conductor.

Electrolyte solution is a liquid medium in which an electrolyte is dissolved, resulting in freely moving ions within a solvent. Electrical conduction in the solution occurs through the migration of these ions between electrodes.

The Energy and Potential Scale provide a framework for relating electrochemical potentials to electronic energy levels, enabling redox processes and semiconductor band positions to be compared on a common energy scale expressed in electron volts (eV).

Equivalent circuit in electrochemistry is a simplified electrical model that represents the behavior of an electrochemical cell using components like resistors, capacitors, and constant phase elements to simulate charge transfer, double-layer capacitance, and diffusion processes.

Faradaic current is the electric current that results from electron transfer during oxidation or reduction reactions at an electrode.

Flat band potential is the electrode potential at which the energy bands of a semiconductor are flat, meaning there is no band bending and no space-charge region.

Forward bias is the application of a voltage to a p–n junction that lowers the potential barrier and allows significant current to flow through the device.

Helmholtz capacitance is the capacitance associated with the compact layer of ions (the Helmholtz layer) at the electrode–electrolyte interface, where a structured arrangement of solvent molecules and counterions stores charge like a parallel-plate capacitor.

Impedance, in its simplest definition, is the resistance and phase-shifted reactance of a system to an AC perturbation. It is the frequency-dependent opposition to alternating current (AC) flow in an electrochemical system, encompassing both resistive and reactive (capacitive and inductive) components that describe how the system responds to dynamic electrical signals.

***J–V* curve** (current-voltage curve) is a graph showing the relationship between the current (J) flowing through a device and the applied voltage (V), commonly used to analyze the performance of electronic and electrochemical devices like solar cells and diodes.

Mott–Schottky plot is a graph of the inverse square of the space-charge capacitance ($1/C^2$) of a semiconductor–electrolyte interface versus the applied potential, used to determine properties like flat band potential, doping density, and semiconductor type (n-type or p-type).

Non-faradaic current is the electric current that arises from processes like charging the electrical double layer or adsorption/desorption at the electrode surface, without any net electron transfer or chemical reaction.

Nyquist plot is a graphical representation of an electrochemical system's impedance, where the real part of the impedance is plotted on the x-axis and the imaginary part on the y-axis, typically used to visualize resistive and capacitive behavior over a range of frequencies.

Potential in electrochemistry is the electric potential difference between an electrode and a reference electrode, which drives the movement of electrons and determines the tendency for redox reactions to occur.

Reactance is the component of impedance that arises from capacitive or inductive elements in a circuit, causing a phase shift between voltage and current.

Reference electrode is an electrode with a known and constant potential, used in electrochemical cells to provide a fixed energy reference against which the working electrode’s potential can be measured.

Resistance is the measure of how much a material or component opposes the flow of electric current.

Reverse bias is the application of a voltage to a p–n junction that increases the potential barrier, widens the depletion region, and strongly suppresses current flow except for a small leakage current.

RHE (Reversible Hydrogen Electrode) potential is a pH-dependent reference scale for electrode potentials, where the potential shifts by approximately –59 mV/pH at 25 °C, reflecting the equilibrium between hydrogen ions and hydrogen gas at any given pH.

Series resistance is the resistance that is connected in line with other components in a circuit, causing a voltage drop that adds directly to the total resistance and limits the overall current flow.

Shunt resistance is a resistance connected in parallel with an electrical device, providing an alternate path for current and affecting the overall performance by reducing the voltage across the main component.

Space-charge capacitance is the capacitance that arises by the accumulation or depletion of charge in the space-charge region near the surface of a semiconductor or at an interface of a semiconductor with a metal or another semiconductor.

Space-charge region/ layer is the area near the surface of a semiconductor where mobile charge carriers are depleted or accumulated, creating an electric field due to immobile charged ions.

Tafel plot is a graph of the logarithm of current density versus overpotential in an electrochemical reaction, used to analyze the reaction kinetics and infer the underlying electron-transfer mechanism by examining parameters like the Tafel slope and exchange current density.

Three-electrode measurements are electrochemical experiments that use three separate electrodes—working, reference, and counter (auxiliary)—to precisely control and measure the potential and current at the working electrode.

Valence band is the highest range of electron energy levels in a solid that is fully occupied by electrons at absolute zero temperature, and from which electrons can be excited to the conduction band to enable electrical conductivity.

V_{pp} (Peak-to-Peak Voltage) is the total voltage difference between the highest (positive peak) and lowest (negative peak) points of a waveform, representing the full vertical span of the signal.

V_{rms} (Root Mean Square Voltage) is the effective value of an alternating voltage, representing the equivalent DC voltage that would deliver the same power to a load, calculated as the square root of the average of the squared instantaneous values over a cycle.

Working electrode is the electrode in an electrochemical cell where the reaction of interest occurs, and whose potential is measured against a reference electrode and controlled during experiments. The opposite current is driven over the counter electrode.

References

(1) Hauff, E. von. Impedance Spectroscopy for Emerging Photovoltaics. *J. Phys. Chem. C* **2019**, *123* (18), 11329–11346. DOI: 10.1021/acs.jpcc.9b00892.

(2) Lazanas, A. C.; Prodromidis, M. I. Electrochemical Impedance Spectroscopy-A Tutorial. *ACS Meas. Sci. Au* **2023**, *3* (3), 162–193. DOI: 10.1021/acsmeasuresciau.2c00070.

(3) Lasia, A. *Electrochemical Impedance Spectroscopy and its Applications*; Springer New York, 2014. DOI: 10.1007/978-1-4614-8933-7.

(4) Fabregat-Santiago, F.; Bisquert, J.; Garcia-Belmonte, G.; Boschloo, G.; Hagfeldt, A. Influence of electrolyte in transport and recombination in dye-sensitized solar cells studied by impedance spectroscopy. *Sol. Energy Mater. Sol. Cells* **2005**, *87* (1), 117–131. DOI: 10.1016/j.solmat.2004.07.017.

(5) Park, J.-B.; Park, S.-M. Fourier transform electrochemical impedance spectroscopic studies on platinum electrodes in an acidic medium. *J. Electroanal. Chem.* **2011**, *656* (1-2), 243–251. DOI: 10.1016/j.jelechem.2010.10.026.

(6) Service, E.; Moehl, T.; Tilley, S. D. Operation of Amorphous TiO2-Protected Photocathodes Described with the Maxwell Equivalent Circuit. *ACS Appl. Mater. Interfaces* **2024**, *16* (44), 60084–60093. DOI: 10.1021/acsami.4c07588.

(7) Buteau, S.; Dahn, D. C.; Dahn, J. R. Explicit Conversion between Different Equivalent Circuit Models for Electrochemical Impedance Analysis of Lithium-Ion Cells. *J. Electrochem. Soc.* **2018**, *165* (2), A228-A234. DOI: 10.1149/2.0841802jes.

(8) Macdonald, J. R. Impedance spectroscopy. *Ann. Biomed. Eng.* **1992**, *20* (3), 289–305. DOI: 10.1007/BF02368532.

(9) Orazem, M. E.; Tribollet, B. *Electrochemical Impedance Spectroscopy*, 2nd ed.; John Wiley & Sons, Ltd, 2017.

(10) Barsoukov, E.; Macdonald, J. R., Eds. *Impedance Spectroscopy: Theory, Experiment, and Applications*, 3rd ed.; John Wiley & Sons, 2018.

(11) Levenberg, K. A Method for the Solution of Certain Non-Linear Problems in Least Squares. *Q. Appl. Math.* **1944**, *2* (2), 164–168. DOI: 10.1090/qam/10666.

(12) Marquardt, D. W. An Algorithm for Least-Squares Estimation of Nonlinear Parameters. *J. Soc. Ind. Appl. Math.* **1963**, *11* (2), 431–441. DOI: 10.1137/0111030.

(13) Todinova, A.; Contreras-Bernal, L.; Salado, M.; Ahmad, S.; Morillo, N.; Idígoras, J.; Anta, J. A. Towards a Universal Approach for the Analysis of Impedance Spectra of Perovskite Solar Cells: Equivalent Circuits and Empirical Analysis. *ChemElectroChem* **2017**, *4* (11), 2891–2901. DOI: 10.1002/celc.201700498.

(14) Lasia, A. The Origin of the Constant Phase Element. *J. Phys. Chem. Lett.* **2022**, *13* (2), 580–589. DOI: 10.1021/acs.jpclett.1c03782.

(15) Bisquert, J.; Balaguera, E. H. Brief Guide to Transformation of Constant Phase Element Impedance to Equivalent Capacitor or Inductor. *J. Phys. Chem. Lett.* **2025**, *16* (23), 5779–5783. DOI: 10.1021/acs.jpclett.5c01131.

(16) Harrington, S. P.; Devine, T. M. Analysis of Electrodes Displaying Frequency Dispersion in Mott-Schottky Tests. *J. Electrochem. Soc.* **2008**, *155* (8), C381. DOI: 10.1149/1.2929819.

(17) Hankin, A.; Bedoya-Lora, F. E.; Alexander, J. C.; Regoutz, A.; Kelsall, G. H. Flat band potential determination: avoiding the pitfalls. *J. Mater. Chem. A* **2019**, *7* (45), 26162–26176. DOI: 10.1039/C9TA09569A.

(18) Brug, G. J.; van den Eeden, A.; Sluyters-Rehbach, M.; Sluyters, J. H. The analysis of electrode impedances complicated by the presence of a constant phase element. *J. Electroanal. Chem. Interfacial Electrochem.* **1984**, *176* (1), 275–295. DOI: 10.1016/S0022-0728(84)80324-1.

(19) Hsu, C. H.; Mansfeld, F. Technical Note: Concerning the Conversion of the Constant Phase Element Parameter Y into a Capacitance. *Corrosion* **2001**, *57* (9), 747–748. DOI: 10.5006/1.3280607.

(20) Hirschorn, B.; Orazem, M. E.; Tribollet, B.; Vivier, V.; Frateur, I.; Musiani, M. Determination of effective capacitance and film thickness from constant-phase-element parameters. *Electrochim. Acta* **2010**, *55* (21), 6218–6227. DOI: 10.1016/j.electacta.2009.10.065.

(21) Chang, B.-Y. The Effective Capacitance of a Constant Phase Element with Resistors in Series. *J. Electrochem. Sci. Technol.* **2022**, *13* (4), 479–485. DOI: 10.33961/jecst.2022.00451.

(22) Bard, A. J.; Faulkner, L. R. *Electrochemical Methods: Fundamentals and Applications*, 2nd ed.; Wiley, 2001.

(23) Guidelli, R.; Compton, R. G.; Feliu, J. M.; Gileadi, E.; Lipkowski, J.; Schmickler, W.; Trasatti, S. Defining the transfer coefficient in electrochemistry: An assessment (IUPAC Technical Report). *Pure Appl. Chem.* **2014**, *86* (2), 245–258. DOI: 10.1515/pac-2014-5026.

(24) Sawyer, D. T.; Sobkowiak, A.; Roberts, J. L., Eds. *Electrochemistry for Chemists*; John Wiley & Sons, 1995.

(25) Rheinländer, P. J.; Herranz, J.; Durst, J.; Gasteiger, H. A. Kinetics of the Hydrogen Oxidation-Evolution Reaction on Polycrystalline Platinum in Alkaline Electrolyte Reaction Order with Respect to Hydrogen Pressure. *J. Electrochem. Soc.* **2014**, *161* (14), F1448–F1457. DOI: 10.1149/2.0501414jes.

(26) Dickinson, E. J.; Wain, A. J. The Butler-Volmer equation in electrochemical theory: Origins, value, and practical application. *J. Electroanal. Chem.* **2020**, *872*, 114145. DOI: 10.1016/j.jelechem.2020.114145.

(27) Sun, A. Growth and Properties of Anodic Oxide Films on Platinum. PhD Thesis, The Pennsylvania State University, 2006.

(28) Bondarenko, A. S.; Stephens, I. E. L.; Hansen, H. A.; Pérez-Alonso, F. J.; Tripkovic, V.; Johansson, T. P.; Rossmeisl, J.; Nørskov, J. K.; Chorkendorff, I. The Pt(111)/Electrolyte Interface under Oxygen Reduction

Reaction Conditions: An Electrochemical Impedance Spectroscopy Study. *Langmuir* **2011**, *27* (5), 2058–2066. DOI: 10.1021/la1042475.

(29) Wu, W.; Lu, Q.; Li, G.; Wang, Y. How to extract kinetic information from Tafel analysis in electrocatalysis. *J. Chem. Phys.* **2023**, *159* (22), 221501. DOI: 10.1063/5.0175156.

(30) Prats, H.; Chan, K. The determination of the HOR/HER reaction mechanism from experimental kinetic data. *Phys. Chem. Chem. Phys.* **2021**, *23* (48), 27150–27158. DOI: 10.1039/D1CP04134G.

(31) Anantharaj, S.; Noda, S. iR drop correction in electrocatalysis: everything one needs to know! *J. Mater. Chem. A* **2022**, *10* (17), 9348–9354. DOI: 10.1039/D2TA01393B.

(32) Sun, A.; Franc, J.; Macdonald, D. D. Growth and Properties of Oxide Films on Platinum: I. EIS and X-Ray Photoelectron Spectroscopy Studies. *J. Electrochem. Soc.* **2006**, *153* (7), B260. DOI: 10.1149/1.2200156.

(33) Cruz-Manzo, S.; Greenwood, P. An impedance model based on a transmission line circuit and a frequency dispersion Warburg component for the study of EIS in Li-ion batteries. *J. Electroanal. Chem.* **2020**, *871*, 114305. DOI: 10.1016/j.jelechem.2020.114305.

(34) Mertens, K. *Photovoltaics*:*Fundamentals, Technology, and Practice*, 2nd ed.; John Wiley & Sons, 2018.

(35) Sivula, K. Mott–Schottky Analysis of Photoelectrodes: Sanity Checks Are Needed. *ACS Energy Lett.* **2021**, *6* (7), 2549–2551. DOI: 10.1021/acsenergylett.1c01245.

(36) Ravishankar, S.; Bisquert, J.; Kirchartz, T. Interpretation of Mott-Schottky plots of photoanodes for water splitting. *Chem. Sci.* **2022**, *13* (17), 4828–4837. DOI: 10.1039/D1SC06401K.

(37) Cui, W.; Niu, W.; Wick-Joliat, R.; Moehl, T.; Tilley, S. D. Operando deconvolution of photovoltaic and electrocatalytic performance in ALD TiO2 protected water splitting photocathodes. *Chem. Sci.* **2018**, *9* (28), 6062–6067. DOI: 10.1039/C8SC01453A.

(38) Lin, F.; Boettcher, S. W. Advanced Photoelectrochemical Characterization: Principles and Applications of Dual-Working-Electrode Photoelectrochemistry. In *Photoelectrochemical Solar Fuel Production: From Basic Principles to Advanced Devices*; Giménez, S., Bisquert, J., Eds.; Springer International Publishing, 2016; pp 323–351. DOI: 10.1007/978-3-319-29641-8_7.

(39) Yang, W.; Moehl, T.; Service, E.; Tilley, S. D. Operando Analysis of Semiconductor Junctions in Multi-Layered Photocathodes for Solar Water Splitting by Impedance Spectroscopy. *Adv. Energy Mater.* **2021**, *11* (9), 2003569. DOI: 10.1002/aenm.202003569.

(40) Moehl, T.; Cui, W.; Wick-Joliat, R.; Tilley, S. D. Resistance-based analysis of limiting interfaces in multilayer water splitting photocathodes by impedance spectroscopy. *Sustainable Energy Fuels* **2019**, *3* (8), 2067–2075. DOI: 10.1039/C9SE00248K.
